# Liposomal FRET Assay Identifies Potent Drug‐Like Inhibitors of the Ceramide Transport Protein (CERT)

**DOI:** 10.1002/chem.202003283

**Published:** 2020-11-09

**Authors:** Doaa Samaha, Housam H. Hamdo, Xiaojing Cong, Fabian Schumacher, Sebastian Banhart, Öznur Aglar, Heiko M. Möller, Dagmar Heuer, Burkhard Kleuser, Essa M. Saied, Christoph Arenz

**Affiliations:** ^1^ Insitute for Chemistry Humboldt Universität zu Berlin Brook-Taylor-Strasse 2 12489 Berlin Germany; ^2^ CNRS Institut de Chimie de Nice Université Côte d'Azur 06108 Nice France; ^3^ Department of Toxicology, Institute of Nutritional Science University of Potsdam Arthur-Scheunert-Allee 114–116 14558 Nuthetal Germany; ^4^ Department of Molecular Biology University of Duisburg-Essen Hufelandstrasse 55 45147 Essen Germany; ^5^ Unit ‘Sexually Transmitted Bacterial Infections' Department of Infectious Diseases Robert Koch Institute 13353 Berlin Germany; ^6^ Universität Potsdam Institut für Chemie Karl- Liebknecht- Strasse 24–25, Haus 25 14476 Golm Germany; ^7^ Chemistry Department Faculty of Science Suez Canal University Ismailia 41522 Egypt; ^8^ Department of Pharmaceutical Chemistry College of Pharmacy Helwan University Cairo 11795 Egypt

**Keywords:** enzyme assays, Förster resonance energy transfer (FRET), liposomes, sphingolipids, transport proteins

## Abstract

Ceramide transfer protein (CERT) mediates non‐vesicular transfer of ceramide from endoplasmic reticulum to Golgi apparatus and thus catalyzes the rate‐limiting step of sphingomyelin biosynthesis. Usually, CERT ligands are evaluated in tedious binding assays or non‐homogenous transfer assays using radiolabeled ceramides. Herein, a facile and sensitive assay for CERT, based on Förster resonance energy transfer (FRET), is presented. To this end, we mixed donor and acceptor vesicles, each containing a different fluorescent ceramide species. By CERT‐mediated transfer of fluorescent ceramide, a FRET system was established, which allows readout in 96‐well plate format, despite the high hydrophobicity of the components. Screening of a 2 000 compound library resulted in two new potent CERT inhibitors. One is approved for use in humans and one is approved for use in animals. Evaluation of cellular activity by quantitative mass spectrometry and confocal microscopy showed inhibition of ceramide trafficking and sphingomyelin biosynthesis.

## Graphical abstract

**FRET indicates lipid transfer**: A new FRET‐based lipid‐transfer assay was developed and used to screen a 2 000 member library for inhibitors of the sphingolipid transporter CERT. Hits were confirmed by different assays and used to induce sphingolipid trafficking phenotypes in living cells.

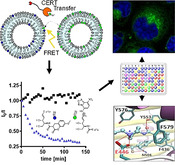

Cellular homeostasis is the result of multiple, regulated metabolic pathways that may be over‐ or under‐represented in pathological contexts. Pharmacological influence on metabolite concentrations is usually achieved by inhibition of rate‐limiting enzymes of a given pathway. However, rate‐limiting enzymes for most metabolic pathways have been identified in the past from cell lysates or with purified enzymes, at the expense of topological information. While topological considerations may be simple for pathways converting soluble substrates, they are crucial and complex for lipid substrates. By definition, lipids are not water‐soluble and transitions between different membranes require either complex vesicular transport mechanisms or specific transport proteins.^[1]^ Like many other biosynthetic pathways, the biosynthesis of sphingolipids extends throughout the membranes of different organelles, which makes specific transport processes essential components of the individual pathway.^[2]^ A growing body of evidence suggests that such transport processes are the rate‐limiting steps of the whole sphingolipid biosynthetic pathway and sphingolipid transport proteins are now considered as pharmacological target molecules.^[3]^

Sphingolipids constitute an abundant and ubiquitous class of lipids in eukaryotic organisms. Their biosynthesis starts with the condensation of serine and palmitoyl CoA in the cytosolic leaflet of the endoplasmic reticulum (ER) membranes.^[4]^ In three further steps, ceramide is formed, which serves as the membrane anchor for the more complex sphingolipids, like glycosphingolipids (GSL) or sphingomyelin (SM), a major component of eukaryotic plasma membranes. However, since synthesis of the latter takes place in different sub‐compartments of the Golgi apparatus (Golgi), ceramide must be transported from the ER membrane to the membranes of the Golgi apparatus. Recently, it has been shown that non‐vesicular transport of ceramide from ER to Golgi is mediated by the ceramide transport protein (CERT) and that this transporter represents the rate‐limiting step for the biosynthesis of sphingomyelin.^[5]^ In fact, malfunctioning of the CERT protein leads to various pathologies^[6]^ and inhibition of this transporter has shown promising results in experimental therapies for the treatment of cancer,^[7]^ hepatitis C virus (HCV)^[8]^ or chlamydia infections.^[9]^ Some time ago, the ceramide analogue HPA‐12 was developed as a potent inhibitor of CERT.^[10]^ Co‐crystal structures of the START domain of CERT together with ceramide or HPA‐12 have been published^[11]^ and numerous studies have been carried out to find an efficient synthesis for the inhibitor^[12]^ or an improvement of its structure.^[13]^ Indeed, improvements of affinity to the START domain have been achieved in cell‐free systems. In most cases, complex assays were employed, in which a fluorescence‐labeled ceramide was used for competitive binding experiments with subsequent pull‐down of the CERT protein. Binding affinity is defined by the quotient of the ceramide fluorescence from the supernatant and from the CERT‐pulldown fraction.^[14]^ Alternatively, liposomal transfer of radiolabeled ceramide between donor and acceptor liposomes is possible.^[15]^ The acceptor liposomes are pulled down by a bead‐bound lectin, since only the acceptor liposomes contain a lectin ligand, glucosylceramide. Subsequently, quantification of the transfer is calculated by radiometric measurements of the two different fractions. Recently, a fluorescence‐based assay of ceramide transfer has also been described, in which a fluorescently labeled ceramide is transferred to liposomes containing a quencher lipid.^[16]^ This assay was performed in cuvettes, but miniaturization has not been described yet, probably due to low transfer rates. The fact that ceramide is one of the most hydrophobic mammalian lipids is a primary obstacle and the use of detergents in liposome‐based applications is contraindicated. Because of these difficulties and the urgent need for more drug‐like CERT inhibitors, a virtual screening has recently been performed, taking advantage of the well‐known 3D binding mode of the HPA‐12 inside CERT's START domain. A lead identified in this process showed a K_D_ of 11 μm, which after optimization could be improved to 60 nm. The cellular activity of the final compound was comparable to HPA‐12, making it the first non‐ceramide analogue inhibitor of CERT.^[17]^ However, nothing is known about the compounds toxicity or whether it is applicable to experiments in mice or humans. Despite the great success of this approach, we reasoned that the search for compounds that mimic an already known binding mode would reveal only a fraction of the possible inhibitors. We therefore aimed at developing a screening assay that should be as realistic as possible in order to be able to detect compounds with completely new binding modes. We reasoned that in any case, a transfer assay would be more predictive than a binding assay. Being inspired by our recent success in developing assays of sphingolipid metabolizing enzymes based on Förster Resonance Energy Transfer (FRET),^[18]^ we wanted to apply this principle to a CERT‐mediated lipid transfer assay. The goal was to monitor the activity of CERT in a homogeneous assay system in multi‐well format and making use of the inherent normalization of fluorescence by dual wavelength readout.^[19]^ The idea was that perhaps a bi‐directional transfer of dye‐labelled ceramides could achieve the necessary transfer rate. For this purpose, we used fluorescent dyes, which we had proven to be effective in our previous FRET probes. Hence, a coumarin ceramide (MCC‐Cer) and an NBD ceramide (NBD‐Cer) were synthesized,^[20]^ respectively, and embedded into separate liposomes (Figure 1).

**Figure 1:**
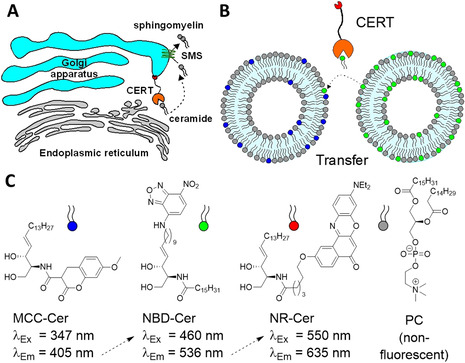
CERT‐mediated transfer assay. A) CERT mediates ER to Golgi transport of ceramide and thus biosynthesis of sphingomyelin by sphingomyelin synthase (SMS). B) Concept of transfer assay. Two types of liposomes are mixed, each containing a differently labeled ceramide. CERT mediated transfer of NBD‐ceramide establishes a FRET system consisting of either coumarin‐ceramide/NBD‐ceramide or NBD‐ceramide/Nile red ceramide. C) Structures of lipid species and maximum excitation and emission wavelengths. Dashed arrows indicate possible FRET.

When coumarin was excited (*λ*_Ex_=347 nm), the corresponding fluorescence (*λ*_Em_=405 nm) could be observed, whereas no significant fluorescence for NBD (*λ*_Em_=536 nm) could be observed. However, after addition of recombinant human CERT, an increase in NBD fluorescence occurred rapidly. The curve changed to a plateau after some time, indicating a termination of the net transfer. The final increase in NBD fluorescence was up to 4fold. Somewhat unexpectedly, the coumarin fluorescence did not change significantly, indicating that overall only a small fraction of the coumarin fluorescence energy was transferred to NBD. The specificity of the NBD fluorescence change was verified by different concentrations of CERT and the possibility of inhibition by HPA‐12 (see Supporting Information for details). Transfer of this assay into 96‐well plates showed the same results without significant loss of lipid transfer activity. To test the hypothesis of bidirectional transfer, one of the two ceramide derivatives was successively replaced by a non‐transferable lipid carrying the same dye. Replacement of coumarin ceramide showed the same results as above, while the replacement of NBD ceramide by non‐transferrable NBD‐lipid resulted in the absence of any fluorescence change (data not shown). We therefore concluded that NBD ceramide (NBD‐Cer) but not coumarin ceramide (MCC‐Cer) is transferred by CERT. Having established a feasible miniaturized CERT assay, we aimed to perform a limited screening for possible inhibitors. However, the absence of transfer the short excitation wavelength of coumarin seemed to us unfavorable, since many aromatic compounds show a significant absorption at such a short wavelength. This would interfere with our screening assay. Since we showed the transferability of NBD ceramide, we considered making this lipid the FRET donor and providing it with a suitable acceptor lipid. For this scenario, Nile red (NR) emerged as a suitable partner and therefore, a NR‐ ceramide was synthesized, accordingly.^[21]^ Fortunately, also with this ceramide FRET pair an assay in 96 well format could be successfully established. Moreover, in an experiment, similar to the one described above, we observed transfer of NR‐ceramide in presence of non‐transferrable NBD‐lipid, which was inhibited in the presence of the inhibitor HPA‐12. Finally, a library of 2 000 compounds with proven pharmacological and/or biological activity was tested at a concentration of 10 μm. Significant inhibition—defined by inhibition like 1 μm HPA‐12 or better—yielded a total of 45 primary hits. Dose‐response evaluation for these hits and sorting out of compounds whose structures did not indicate any promising further development, resulted in a total of 9 compounds that were considered promising and which were further investigated. The inhibitory effects were found to be highly reproducible, with the exact values showing some variation for different batches of the liposome preparation. For each assay, HPA‐12 was therefore used as a reference substance. The assay established by us is in principle an enzyme‐catalyzed mixing of separate lipid pools and is therefore potentially interfered by substances that stimulate vesicle fusion. Such compounds were expected to give false negative results (no inhibition), although a number of compounds with detergent‐like properties showed inhibition, contrary to this hypothesis. Nonetheless, we aimed at further investigation, using orthogonal assays devoid of liposomes in order to proof the validity of our approach. The binding assays described so far seemed to us very complex and tedious for dose‐response studies. Therefore, we considered using the environmental sensitivity of the existing dye‐labeled ceramides for a competitive assay. Nile red is known to show strongest fluorescence in a non‐polar environment, therefore we suspected that Nile red ceramide bound to a hydrophobic pocket of CERT would be much more fluorescent than in the unbound state. To test this hypothesis, a 1 μm micellar solution of Nile red ceramide was treated with increasing concentrations of CERT. In fact, an up to 20‐fold increase in fluorescence was observed, which could be reverted to approximately the initial value using HPA‐12. Notably, this effect was much less pronounced using the less active R,R diastereomer of HPA‐12 (see Figure S8). Hence, the fluorescence of Nile red ceramide is therefore a suitable tool to evaluate competitive CERT binders in a homogenous fashion. After the NR ceramide competition assay, four compounds were selected for further evaluation in cells. Three compounds, 8E8, 20D5 and 6B11 showed high activity in both, the transfer and the competition assay (Figure 2 B&C). Furthermore, we selected 17C9, which showed a weak dose‐response in the transfer assay, but a very strong competition to the binding of NR ceramide. Finally, we fluorescently labeled the recombinant START domain of CERT and monitored binding to the candidate compounds, using microscale thermophoresis (MST). For HPA‐12, a K_D_ of ≈40 nm was determined, which is in rather good agreement with K_D_ values determined in a recently developed surface plasmon resonance assay, in which the START domain was immobilized on solid phase.^[17]^ Our new solution phase MST‐based binding assay confirmed binding for all compounds except 17C9, but the affinities for 8E8 (K_D_≈5 μm), 20D5 (K_D_≈150 μm) and 6B11 (K_D_≈5 μm) were significantly lower than for HPA‐12 (Figure S6). This could reflect different binding modes to CERT, which may include components other than the START domain.

**Figure 2:**
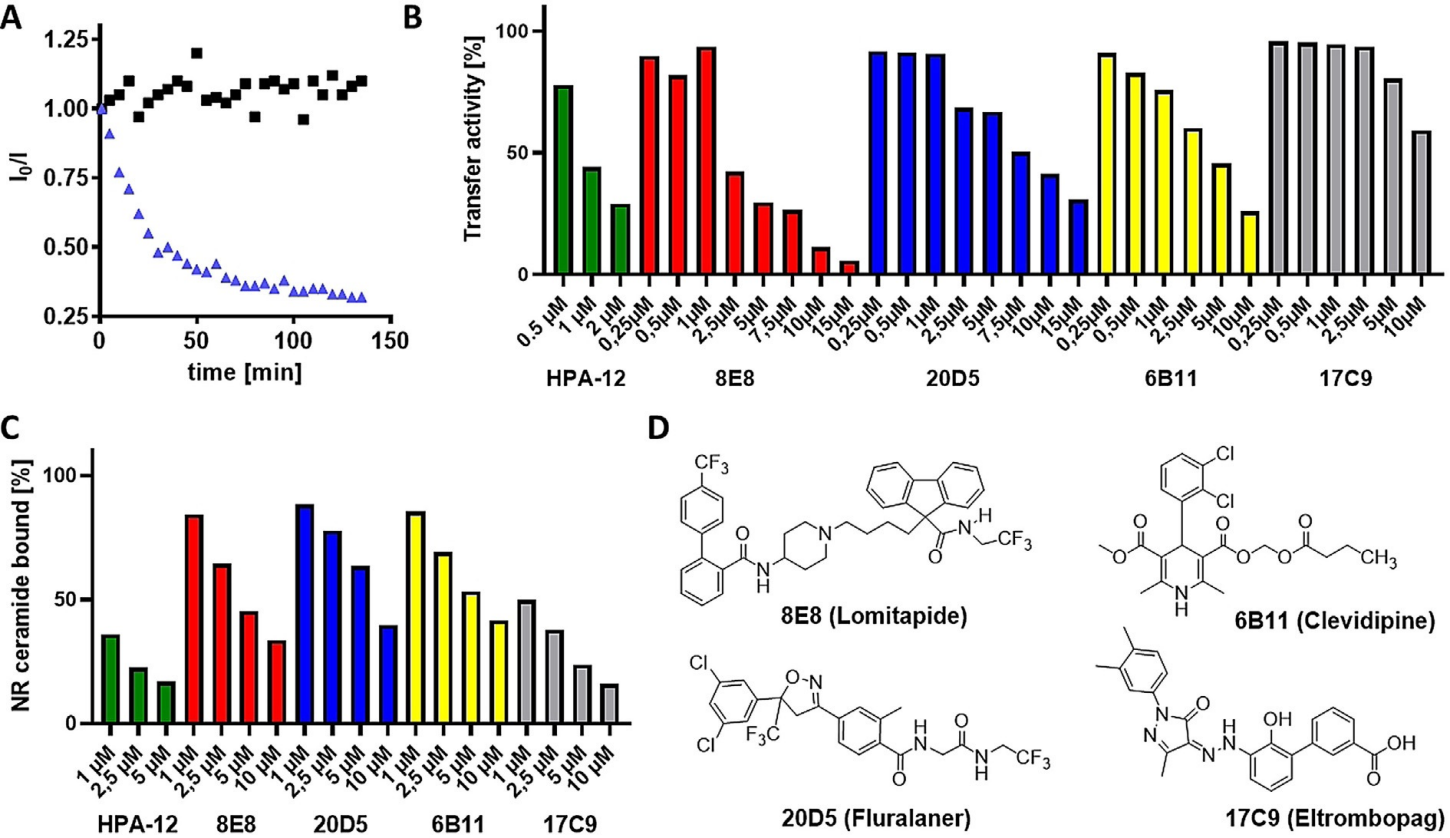
In vitro assays. A) Typical time course of the MCC‐Cer/NBD‐Cer transfer assay. Change of fluorescence intensity of NBD‐Cer (*λ*_Em_=535 nm) after excitation at 350 nm (MCC). Black squares: without CERT; blue triangles: in presence of CERT. Assay results were usually taken after 30 or 45 minutes and defined by a reduction in total fluorescence at the respective time point, compared to the reaction without inhibitor. B) Transfer assay (NBD‐Cer/NR‐Cer) results for final hits plus HPA‐12. C) Results of NR‐ceramide fluorescence competition assay. D) Structures of final compounds.

Finally, we tested the activity of these compounds in cell‐based assays (Figure 3). For this purpose, HeLa cells were incubated with the identified compounds or HPA‐12 for 48 hours in a serum‐reduced medium, and the effect on individual sphingolipid species was analyzed by quantitative mass spectrometry.^[22]^ Indeed, 8E8 and 20D5 showed a dose‐dependent reduction of total sphingomyelin, while treatment with 6B11 or 17C9 showed no significant changes in total sphingomyelin. Compared to HPA‐12, 20D5 showed a similar total sphingomyelin for all concentrations used. In contrast, 8E8 showed lower effects than HPA‐12 at lower concentrations, but a drastic reduction of sphingomyelin at 5 μm. In addition, relation of total sphingomyelin to ceramide showed much clearer dose dependencies for both 20D5 and 8E8 and an increased potency for 8E8 over all concentrations compared to HPA‐12 and similar or higher potency for 20D5. For the compounds finally investigate, the transfer assay was more predictive for the reduction in SM biosynthesis, than the NR‐ceramide competition assay. Surprisingly, 6B11 and 17C9 led to a dramatically increased SM/Cer rate, which was due to a reduction of almost all ceramide species investigated. At present we do not have a conclusive explanation for this behavior, but a future investigation of this phenomenon could be interesting.

**Figure 3:**
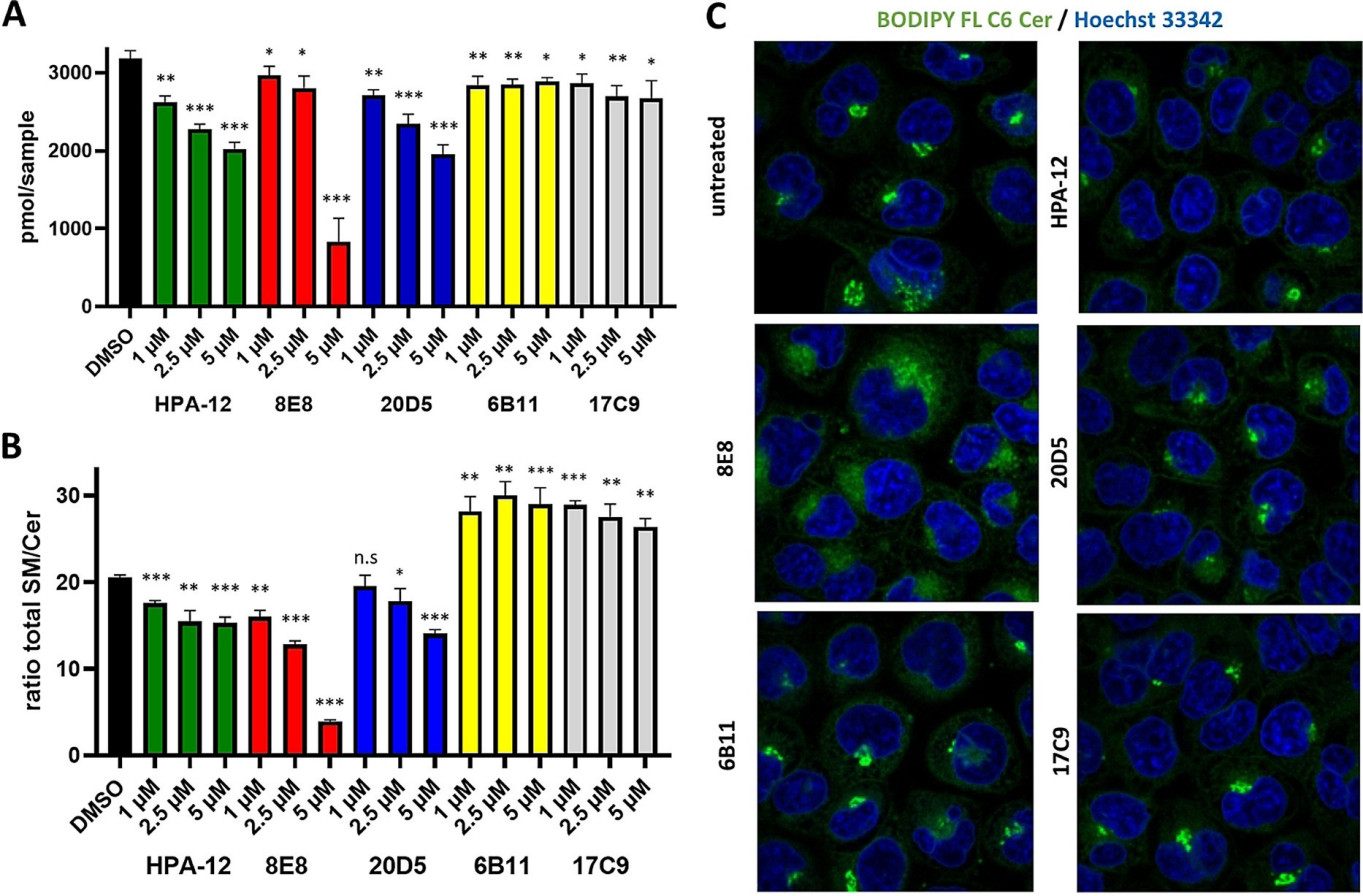
Cellular activity of hit compounds. A) Effects on cellular content of sphingomyelin (500 000 cells per sample). B) Effects on cellular ratios of total sphingomyelin/ total ceramide. C) Effect on cellular staining by BODYPY FL ceramide. Asterisks indicate significant differences between samples calculated by student's t test. [*] *p*<0.05; [**] *p*<0.01; [***] *p*<0.005.

Because all substances used were compounds with known biological activity, it cannot be excluded that the observed changes in the sphingolipidome are due to indirect effects. To test the hypothesis that the identified compounds actually inhibit transport of ceramide in living cells, we treated HeLa cells with fluorescence‐labelled ceramide (BODIPY‐Cer) in the presence of the four final compounds and examined the effects by confocal microscopy. In fact, 6B11 and 17C9 showed only minor effects and resembled the untreated controls, but compounds 8E8 and 20D5 significantly influenced the intracellular distribution of BODIPY‐Cer (Figure 3 C and Figures S10&S11). While the phenotype induced by 20D5 resembled that of HPA‐12, there was a major change in phenotype induced by 8E8. 20D5 (Fluralaner) is used in veterinary medicine and targets arthropod parasite chloride channels and has no known target in mammals.^[23]^ In contrast, 8E8 (lomitapide) is targeting the human microsomal triglyceride transfer protein (MTTP).^[24]^ This protein is located in the ER lumen and catalyzes the loading of triglycerides, cholesterol esters and phospholipids into lipoproteins.^[25]^ It has been shown that MTTP deficiency reduces plasma levels of ceramide and to a lesser extend of sphingomyelin and that the purified protein is capable of transferring radioactively labeled sphingomyelin and ceramide between liposomes.^[26]^ Based on our results that these molecules also interact with purified human CERT and inhibit CERT‐mediated ceramide transfer, we established binding models for these compounds by molecular docking and molecular dynamics (MD) simulations.

MD simulations revealed that the CERT START domain in *apo* form could close‐open at the Ω1 loop (Figure 4 A). The Ω1 loop showed high temperature factors in the crystal structure^[27]^ and might act as a gate for ligand binding. Both, 8E8 (lomitapide) and 20D5 (Fluralaner) maintained stable binding in the ceramide‐binding pocket, with some fluctuations near the highly mobile Ω1 loop. Their amide group mimicked that of the ceramide to form a hydrogen bond with residue Y553 (Figure 4 B and 4C). However, the affinity was mostly attributed to hydrophobic interactions, especially with Y576 and F579 (see Figure S7 for key interactions and binding energies). In addition, 8E8 bound ionically with E446 and had significantly lower binding free energy (−26.3±14.5 kcal mol^−1^) than 20D5 (−8.3±6.8 kcal mol^−1^), which may account for the stronger inhibitions by 8E8.

**Figure 4:**
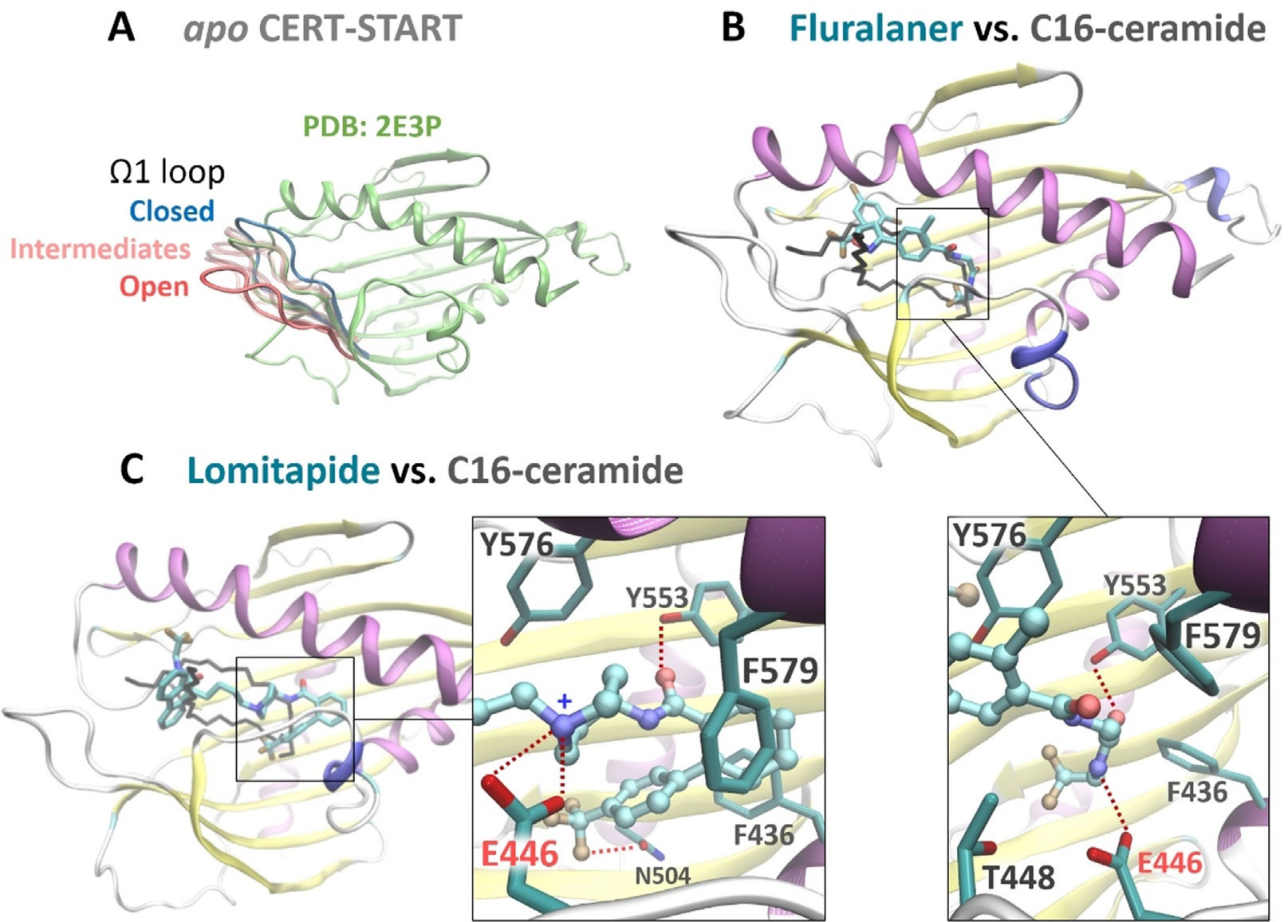
Representative conformations from the MD simulations. A) CERT START in *apo* form displayed transient (15 % of simulation time) opening at the Ω1 loop. B), C) Binding pose of 20D5 (fluralaner) and 8E8 (lomitapide) superimposed on C16‐ceramide (black line).

In summary, we have developed a new FRET‐based ceramide transfer assay to identify new CERT inhibitors. For two of these compounds, we showed effective inhibition of CERT‐mediated transfer in vitro, replacement of CERT‐bound Nile red ceramide and interaction with the fluorescently labeled START domain of CERT by MST. Finally, two compounds resulted in an increase of cellular ceramide at the expense of sphingomyelin concentrations. These two compounds were significantly more active than HPA‐12 at higher concentrations (5 μm). Moreover, confocal microscopy of treated cells revealed that the compounds altered ceramide trafficking. Noteworthy, these compounds are approved for pharmacological use in humans (Lomitapide) or animals (Fluralaner). By modelling the identified structures into the START domain of CERT, we established a conclusive binding model, which may be used for structure‐guided design of future CERT inhibitors with increased affinity and selectivity. Our results motivate to screening of larger libraries and to apply the principles developed here to further lipid transferases of biomedical interest.

## Conflict of interest

The authors declare no conflict of interest.

## Supporting information

As a service to our authors and readers, this journal provides supporting information supplied by the authors. Such materials are peer reviewed and may be re‐organized for online delivery, but are not copy‐edited or typeset. Technical support issues arising from supporting information (other than missing files) should be addressed to the authors.

Supplementary
