# Supplementary material for: Liposomal FRET Assay Identifies Potent Drug‐Like Inhibitors of the Ceramide Transport Protein (CERT)
Source: Chemistry. 2020 Nov 9;26(70):16616–21. doi: 10.1002/chem.202003283 (PMC7756341; doi:10.1002/chem.202003283)
Supplement: Supplementary file 1 — Supplementary [file CHEM-26-16616-s001.pdf]

Chemistry–A European Journal

Supporting Information

Liposomal FRET Assay Identifies Potent Drug-Like Inhibitors of the Ceramide Transport Protein (CERT)

Doaa Samaha,[a, h] Housam H. Hamdo,[a] Xiaojing Cong,[b] Fabian Schumacher,[c, d] Sebastian Banhart,[e] Öznur Aglar,[f] Heiko M. Möller,[f] Dagmar Heuer,[e] Burkhard Kleuser,[c] Essa M. Saied,*[a, g] and Christoph Arenz*[a]

Contents	Page
1. Protein expression	S2
2. Assessment of CERT-transfer Activity	S4
3. Assessment of CERT-competition Activity	S10
4. Microscale Thermophoresis (MST) assays	S14
5. Cellular experiments	S16
6. Details for experimental lipidomics	S18
7. Docking, molecular dynamics and binding free energy calculations	S20
8. General methodology for organic syntheses	S22
9. Synthetic procedures and analytical data	S24
10. References	S39
11. NMR spectra for synthesized compounds	S40

1. Protein expression

1.1 Expression and purification of CERT protein from *E. coli* cells

His6-tagged recombinant human CERT was purified as described previously [1]. Briefly, BL21 (DE3) *E. coli* cells transfected with human CERT cDNA subcloned into pET-28a(+) (Novagen) were cultured in 500 ml of Luria-Bertani broth containing kanamycin (25 µg/ml) at 37°C to OD 0.6 at 600 nm. The culture was briefly chilled on ice. Then, after addition of isopropyl β-D-thiogalactoside to a final concentration of 250 µM, the cells were cultured for 16 h at 25°C. Hereafter, all steps were done at 4°C or on ice if not otherwise noted. The cells were harvested by centrifugation (4000 rpm, 20 min) and suspended in 25 ml of 25 mM Tris-HCl buffer (pH 7.4) containing 1% Triton X-100, 270 mM sucrose, 2.5 mM DTT and protease- and phosphatase inhibitor (Halt™ inhibitor cocktail, EDTA-free, Thermo Fisher Scientific). After breaking the cells using a French Press, lysates were cleared by centrifugation at 45.000 rpm (84.000 g), for 30minutes at 4°C. The supernatant was adjusted to Ni-nitrilotriacetic acid (NTA) column chromatography by treatment buffer A containing imidazole (50 mM sodium phosphate, pH 7.4, containing 300 mM NaCl and 500 mM imidazole) and with 15fold concentrated phosphate buffer (50 mM, pH 7.4 containing 4.42 M NaCl). The final lysate now contained 20 mM imidazole and 300 mM NaCl. Purification was taken out on a fast protein liquid chromatography (FPLC) equipped with a HisTrap HP column (GE Healthcare), according to manufacturer's protocols. After elution (one-step gradient with 150mM imidazole), the fractions containing the protein were desalted using a HiTrap desalting column (GE Healthcare), using Tris-HCl buffer (10 mM, pH 7.4 containing 250 mM sucrose). The desalted fraction containing the purified CERT was divided into different aliquots and stored at –80°C until use. Protein concentrations were determined using the bicinchoninic acid protein assay kit (Sigma) with bovine serum albumin as the standard.

1.2 Expression and purification of the START domain of human CERT

The START domain of hCERT was expressed has a His6-SUMO fusion protein [2]. The pQE-based plasmid harboring the genetic information of His6-SUMO-START was transformed into E. coli BL21(DE3) by following the standard heat shock method. Colonies were grown on carbenicillin (50 mg/ml) and 1% glucose supplemented Luria–Bertani (LB) agar plates. A single colony was used to inoculate 20 ml of pre-culture 1 % of which was then used to inoculate 500 ml of LB medium (50 mg/ml carbenicillin, 10 mM glucose, 37 °C, 160 rpm). Upon reaching OD600 = 0.8, the culture was induced with 500 μM IPTG (isopropyl-β-d-thiogalactoside) and cells were incubated for 16 h with continuous shaking at 160 rpm. The cells were harvested by centrifugation at 10000 g, 4 °C for 20 min.

For purification of the protein, the cell pellet was re-suspended in lysis buffer (25 mM Tris/HCl, pH 7.4, 270 mM Sucrose, 1% Triton X-100, 2.5 mM dithiothreitol (DTT), Protease/Phosphatase inhibitor) on ice. Cells were disrupted using an EmulsiFlex-C3 instrument (Avestin Inc.; CANADA). The lysate was cleared by centrifugation, and the supernatant was loaded onto a Ni–NTA column (Qiagen). The protein was washed with loading buffer pH 7.4 (50 mM Na_2HPO_4, 50 mM NaH_2PO_4, 150 mM NaCl, 2,5 mM DTT) containing 20 mM imidazole and eluted with elution buffer pH 7.4 (50 mM Na_2HPO_4, 50 mM NaH_2PO_4, 150 mM NaCl, 2.5 mM DTT) containing 150 mM imidazole. Fractions containing the protein of interest according to SDS-PAGE were pooled and digested with Ubiquitin-like-protein specific protease 1 (ULP1). The by-products (His6-SUMO and ULP1) were removed in a second IMAC step, and the target protein was subjected to a final purification by gel filtration on a superdex 200 16/600 prep grade column equilibrated with loading buffer pH 7.4 (50 mM Na_2HPO_4, 50 mM NaH_2PO_4, 150 mM NaCl, 2.5 mM DTT). The monomeric form of the START domain of hCERT was collected. The final concentration of the protein was determined via UV-Visible spectrophotometry (Varian Cary 50) at 280 nm using a calculated extinction coefficient of 50420 (M^{-1} cm^{-1}).

2. Assessment of CERT-transfer Activity

2.1 Reagents and materials

Egg phosphatidylcholine (PC) and egg phosphatidylethanolamine (PE) were purchased from Sigma. Fluorescence (nitrobenzoxadiazole (NBD), 7-Methoxycumarin-3-carboxylat (MCC), 9-Diethylamino-5-oxo-5H-benzo[a]phenoxazin-2-yl (NR)) labeled-ceramide, were chemically synthesized (section 5). (1*R*,3*S*) and (1*R*, 3*R*) -N-(3Hydroxy-1-hydroxymethyl-3-phenylpropyl)-dodecamide (HPA-12) were synthesized as previously described [3].

2.2 Liposome preparation

Two donor and acceptor liposomes were prepared one acts as donor liposome (NBD-Cer or MCC-Cer) and the other as acceptor liposome (NR-Cer or NBD-Cer). The donor and acceptor liposomes were mixed in proper percentage for establishing two fluorescence resonance energy transfer (FRET) systems (MCC/NBD-ceramide system, or NBD/ NR-ceramide system). The stock solutions of the phospholipids and the fluorescently-labeled ceramide were prepared by dissolving in a mixture of methanol:chloroform (1:1). The liposomes were prepared by mixing 1333 µmol phosphatidylcholine, 333 µmol phosphatidylethanolamine, and 333 µmol fluorescently-labeled ceramide (4:1:1 molar ratio) in siliconized Eppendorf tubes. The mixture was dried under argon stream to yield a waxy lipid film. The obtained waxy residue was hydrated with Tris.HCl buffer (1mL, 2 mM, pH 7.4) to a final concentration of 2 mM and the resulting mixture was vortexed for 1 min. The lipid mixture was subjected to 6 freeze-thawing cycles with liquid nitrogen and shaking incubator at 37ºC. Afterwards, the lipid suspension was sonicated with a Cup-Horn sonicator for 20 sec, followed by vigorous shaking on vortex for 1 min. Finally, the lipid mixture was extruded with a mini extruder (Avanti polar lipids) by forcing the suspension through two polycarbonate filters with 100 nm pore size for 13 times to afford a solution of liposome type.

2.3 Assessment of CERT transfer assay (cuvette format)

Two FRET-based CERT-transfer systems were designed and examined in cuvette format, the first one was (MCC-Cer:NBD-Cer 1:9) where MCC-ceramide is the FRET donor and NBD-ceramide is the FRET acceptor, while the other one was (NBD-Cer:NR-Cer 1:4) where NBD-ceramide is the FRET donor and NR-ceramide is the FRET acceptor (**Fig S1, S2** and **S4**).

The purified recombinant hCERT in 10mM Tris.HCl buffer (pH 7.4, 250 mM sucrose) was diluted with 10mM Tris.HCl buffer (pH 7.4, 300mM NaCl) to a final concentration of 0.225 µg/µL. The liposomes were freshly prepared directly before use to avoid fusion of the vesicles. 2700µL of 10mM Tris.HCl buffer (pH 7.4, 300 mM NaCl) were transferred into 4 quartz cuvettes (1x1 cm, 3 mL) and blank scan spectra were recorded. All spectra were recorded at 37°C temperature with magnetic stirring with excitation and emission slit widths of 5 nm, and the PMT voltage was 700 V. The liposomes mixture was then added into the cuvettes (with a final concentration of 2µM) and the initial scan spectra were recorded (Ex/Em in nm: 347/(500-800) for MCC-/NBD-ceramide system or Ex/Em in nm: 466/(500-800) for NBD-/NR-ceramide system). hCERT aliquot was subsequently added to the cuvettes (with final concentration of 0.2 µg/µL), which were tightly closed by the stoppers and mixed with caution, then placed in a multi-cell holder. Controls were liposomes mixture in 10mM Tris.HCl buffer and CERT aliquot in 10mM Tris.HCl buffer. After 2min of pre-incubation, kinetics data points (347/405 + 347/536) for MCC-/NBD-ceramide system or (466/536 + 466/600) for NBD-/NR-ceramide system were recorded in time intervals for 18h at the same conditions. After the measurement was completed, the fluorescence data were normalized and plotted. The transfer efficiency was calculated using the formula: $I_x = I_0(\grave{A}_1\text{-}X)/ I(\grave{A}_1\text{-}Y)$, where $\grave{A}_1$ is the of the florescence of the control of liposome mixture in Tris.HCl buffer, X is the of the fluorescence of the buffer alone, $\grave{A}_2$ is the fluorescence of the assay mixture, Y is the fluorescence of the CERT aliquot in Tris.HCl buffer.

In order to examine the assay efficiency, the experiment was performed again at the same conditions using HPA12 (DMSO, with a final concentration of 2.5µM) as a CERT-inhibitor. The inhibition efficiency was calculated using the formula: $I_x = I_0(\grave{A}_1\text{-}X)/ I(\grave{A}_2\text{-}Y)$, where $\grave{A}_1$ is the of the florescence of the control of liposome mixture in Tris.HCl buffer, X is the of the fluorescence of the buffer alone, $\grave{A}_2$ is the fluorescence of the assay mixture with HPA12, Y is the fluorescence of the CERT aliquot in Tris.HCl buffer (**Fig S2** and **Fig S3**).

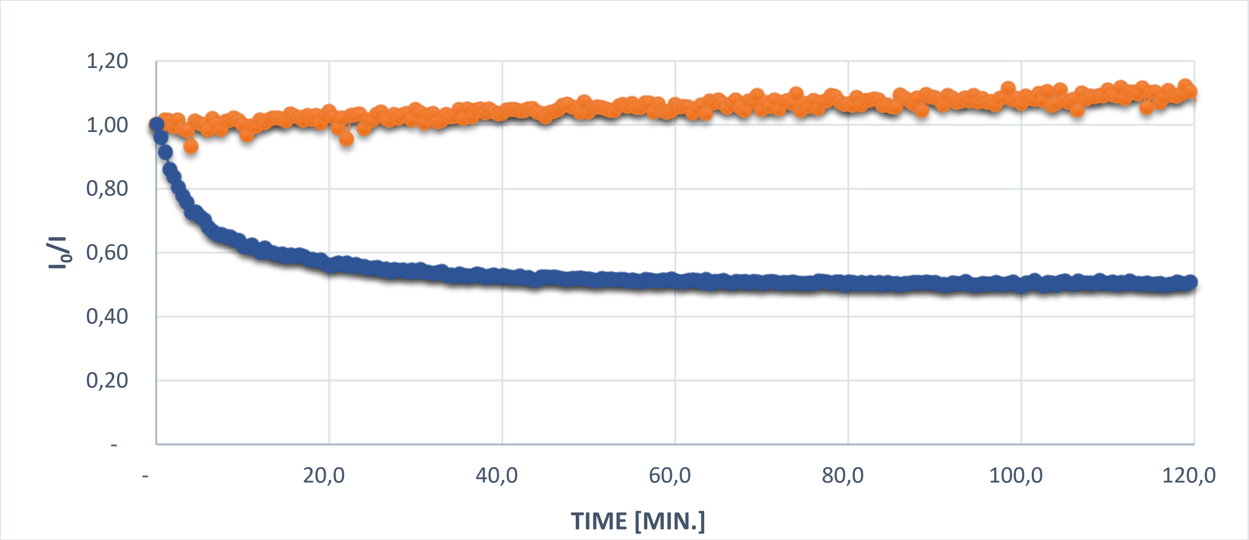

Figure S1. Kinetic study for CERT-mediated transfer of fluorescently-labeled ceramides between donor and acceptor liposomes (1:9 MCC-Cer/NBD-Cer). Normalized intensity of NBD-ceramide emission at 536 nm with (blue) or without (orange) CERT (0.225 µg/µL).

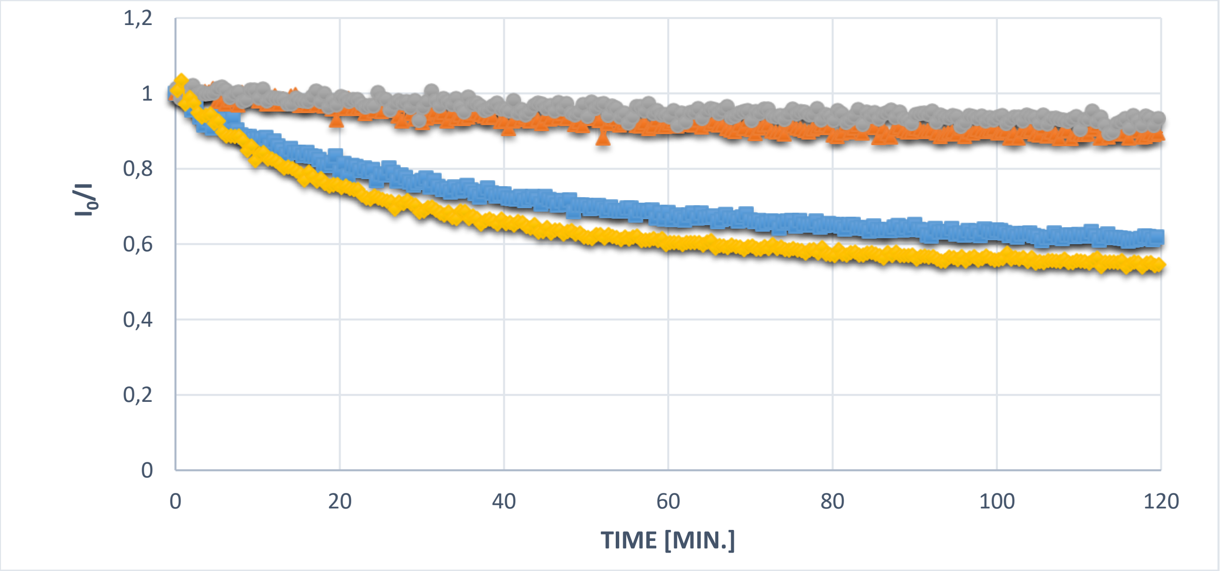

Figure S2. Inhibition of CERT-mediated ceramide transfer by HPA12 stereoisomers. Normalized intensity of NBD-ceramide emission of liposome mixture (1:9 MCC-Cer/NBD-Cer) at 536nm without (grey) or with (yellow) CERT (0.225 µg/µL), and the effect of 2.5µM HPA12 (1*R*, 3*S*) (orange) and 2.5µM HPA12 (1*R*, 3*R*) (blue) in presence of CERT.

In order to determine the transferability of individual lipids by CERT, NBD-ceramide was replaced by a nontransferable NBD-labeled lipid and the assay was performed under the same

conditions. As shown in **Fig S3**, both types of liposome mixtures gave rise to a similar FRET-signal, allowing the conclusion that NR-ceramide is a CERT-transferable lipid. The same experiment (not shown) in which NBD-Cer was replaced by the nontransferable lipid and mixed with liposomes containing MCC-Cer resulted in no change of fluorescence, which led us to the concusion that MCC-Cer is not transferred by CERT.

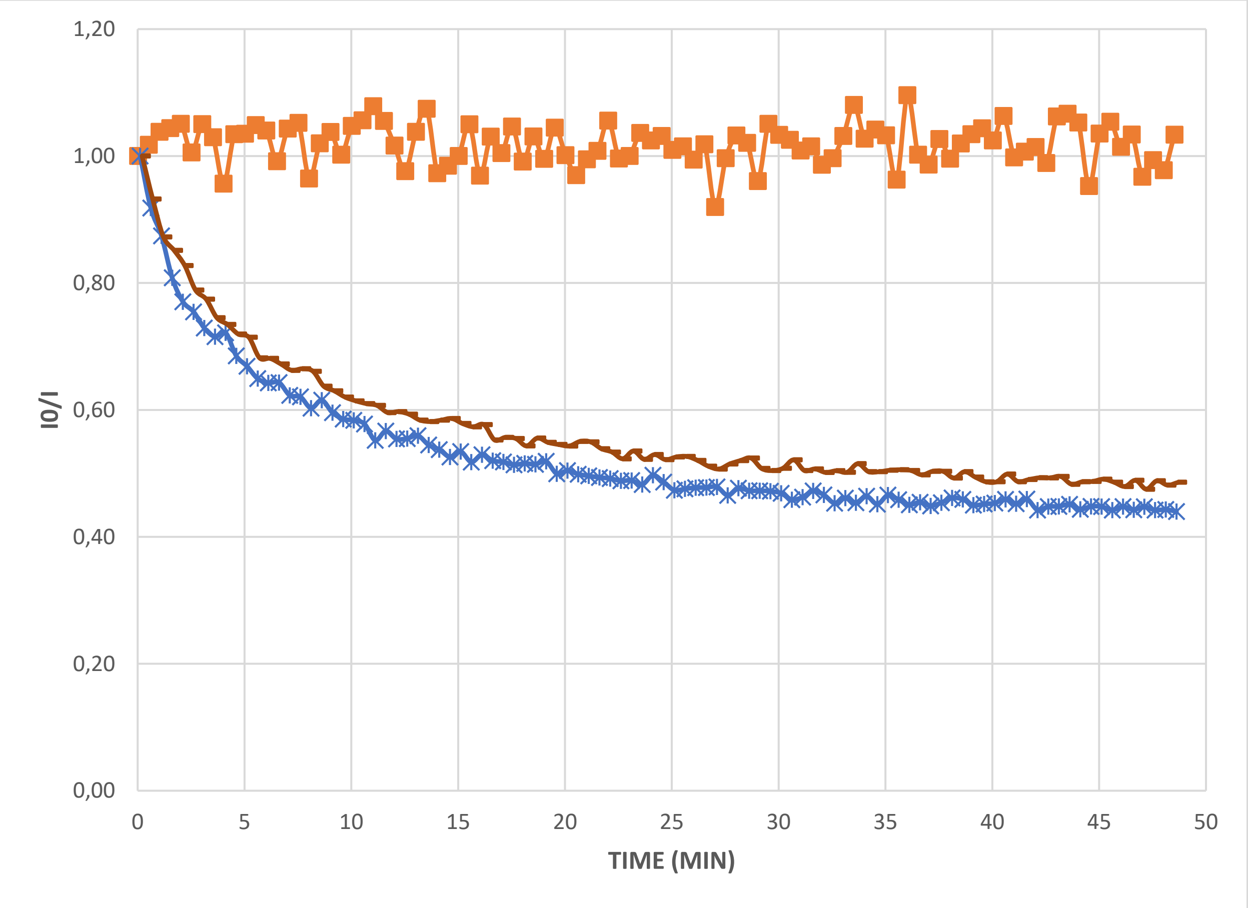

Figure S3. CERT-mediated transfer of NR-labeled (NR-Cer) between liposomes in a mixture of 1:4 NBD-labeled non transferrable lipid/NR-Cer (dark red) or 1:4 NBD-Cer/NR-Cer (blue). Normalized intensity of NR-ceramide emission at 640 nm without (orange) and or with (blue and red) CERT (0.225 µg/µL).

2.4 Development of high throughput screening CERT-assay (96-well plate format)

The assay was performed in non-binding clear-bottom 96-well microplate (Greiner Bio-One). The donor and acceptor liposomes were mixed directly before performing the assay. To each well was sequentially added 10 µL liposome mixture, 1µL HPA12 (at final concentration 0.5µM, 1µM and 2µM) or DMSO, and 89 µL of the CERT aliquot (20µg/well). Controls were triplicates of 10mM Tris.HCl buffer alone, CERT aliquot (20µg/well) with 1µL DMSO, and liposome mixture in 10mM Tris.HCl buffer (pH 7.4, 250 mM sucrose). The plate was then covered using transparent Thermowell seal tape. The plate was placed in the microplate reader (LABTECH) at 30°C with shaking. The fluorescence was measured from the bottom over 1h (number of cycles 35, delay 70 sec) with excitation filter 485 nm and emission filter 640 nm (for NBD-Cer/NR-ceramide FRET system) or excitation filter 347 nm and emission filter 536 nm (for Coumarin (MMC-Cer), NBD-ceramide (NBD-Cer) FRET system). The transfer efficiency was calculated using the formula: $I_x = I_0(\grave{A}_1\text{-}X)/I(\grave{A}_2\text{-}Y)$, where $\grave{A}_1$ is the average of the florescence of the control of liposome mixture in Tris.HCl buffer, X is the average of the fluorescence of the buffer alone, $\grave{A}_2$ is the fluorescence average of the assay mixture, Y is the fluorescence average of the CERT aliquot with inhibitor/DMSO (**Fig S4**).

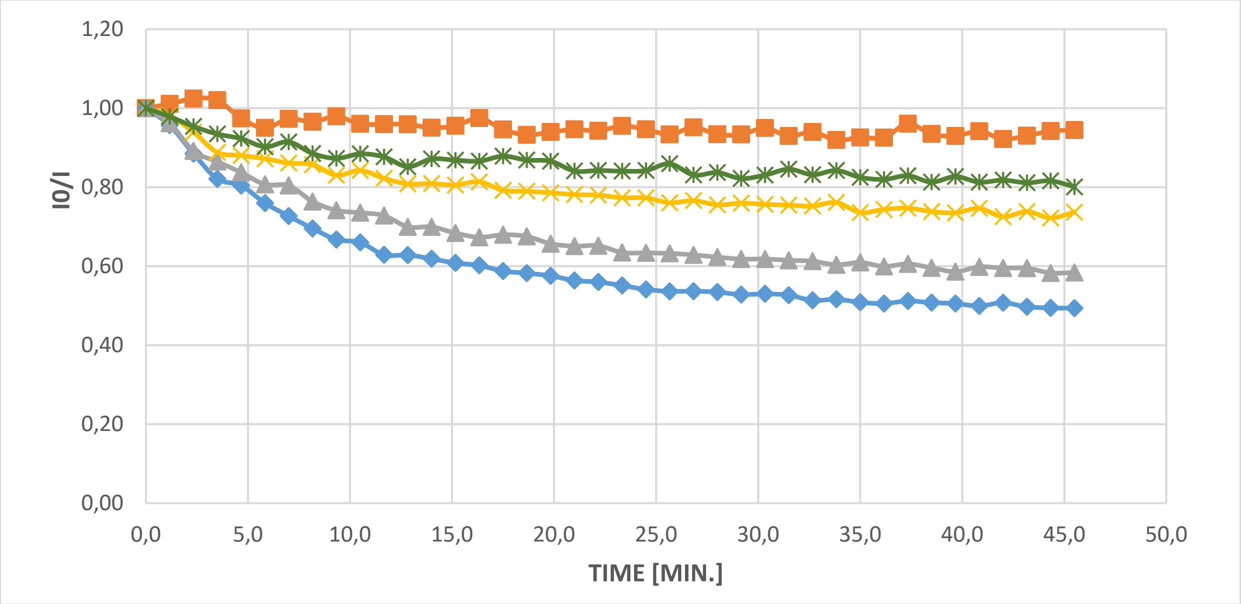

Figure S4. CERT-mediated transfer of fluorescently-labeled ceramide between donor and acceptor liposomes (1:4 NBD-Cer/NR-Cer). Normalized intensity of NR-ceramide emission at 640 nm without (orange) or with (blue) CERT (0.225 µg/µL), and effects of (1*R*, 3*S*) HPA12 at different concentrations 0.5 µM (gray), 1µM (yellow), 2 µM (green) in presence of CERT.

2.5 Library of small molecules

A library of 2000 compounds were selected and obtained from MedChem Express (Monmouth Junction, NJ, USA). A 70% of the selected compounds was FDA approved drugs and 30% was pharmacologically active compounds (LOPAC®). The library was supplied in 10 mM concentration dissolved in 100% dimethyl sulfoxide (DMSO) (10 µL solution) in 96-well plates sealed with Thermowell seal tape. The library was diluted with DMSO to a 1 mM final concentration and was stored in dark at -80°C.

2.6 High throughput screening of small molecules library

A library of 2000 compounds were screened for their inhibitory activity for CERT- transfer activity using the established high throughput assay. The assay was performed as mentioned in the previous sub-section **2.5** using NR-Cer/NBD-Cer system. Briefly, 10µL liposome mixture (4:1 NR-Cer: NBD-Cer), 1 µL tested compound or DMSO for control, and 89µL CERT stock solution were sequentially added into a non-binding 96-well microplate. Controls per plate were triplicates of 10mM Tris.HCl buffer (pH 7.4, 250 mM sucrose) with 1µL DMSO, CERT aliquot (20µg/well) with 1µL DMSO, liposome mixture in 10mM Tris.HCl buffer (pH 7.4, 250 mM sucrose) with 1µL DMSO, and HPA12 as a reference inhibitor (final concentration of 0.5µM, 1µM, and 2µM). The plate was sealed with transparent Thermowell seal tape and the assay was allowed to proceed as described above using excitation filter 485 nm and emission filter 640 nm. It should be noted that the final concentration/well was 2 µM of fluorescent lipid (1.6µM NR-ceramide: 0.4µM NBD-ceramide), 10 µM of the tested compound (with DMSO 1%), and 0.2 µg/µL of CERT. The transfer efficiency was calculated using the formula: $I_x = I_0$ ($\grave{A}_1$-X)/I($\grave{A}_2$-Y), where $\grave{A}_1$ is the average of the florescence of the control of liposome mixture in Tris.HCl buffer, X is the average of the fluorescence of the buffer alone, $\grave{A}_2$ is the average of the fluorescence of assay mixture treated with tested compound or DMSO, Y is the average of the fluorescence of CERT aliquot with inhibitor/ DMSO (**Fig S5**). The library was grouped as ‘non-hit group’ if no CERT-transfer inhibition was detected or was ≤50%, or ‘hit group’ if detected CERT-transfer inhibition was ≥50%. The activity of ‘hit group’ was confirmed by repeating the experiment again in triplicated at concentration ranging from 0.25µM to 10µM. Accordingly, the ‘hit group’ was grouped again as ‘False-hit group’ if the result was not reproducible or activity was not dose-dependent, or ‘confirmed-hit group’ if the result was reproducible and in dose-dependent behavior.

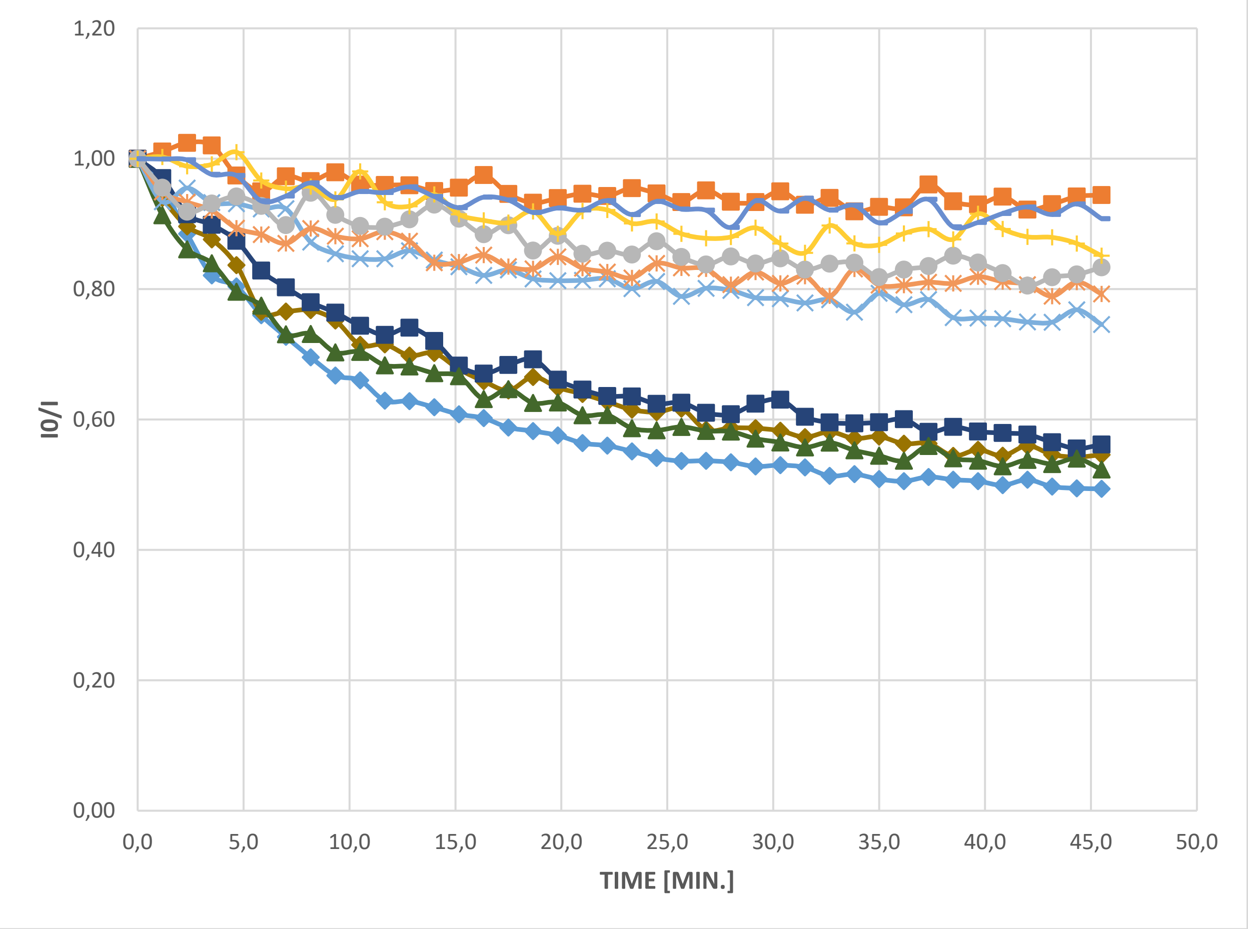

Figure S5. Inhibition of CERT-mediated transfer of fluorescently-labeled ceramides (1:4 NBD-Cer/NR-CEr) by 8E8. Normalized intensity of NR-ceramide emission at 640 nm without (orange) or with (blue) CERT (0.225 µg/µL), and effects of 8E8 at different concentrations (0.25 µM (brown), 0.5 µM (dark blue), 1µM (dark green), 2.5 µM (green), 5 µM (red), 7.5 µM (grey), 10 µM (yellow), 15 µM (blue) in presence of CERT.

3. Assessment of CERT-competition Activity

3.1 Development of a NileRed Ceramide competition assay

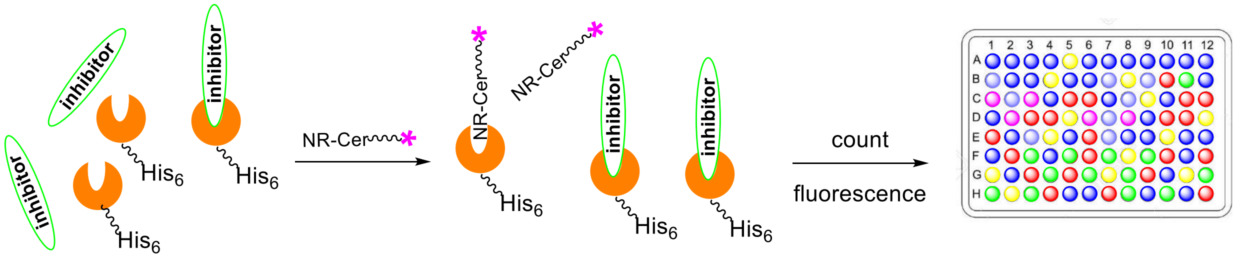

Figure S6. Illustration of competition assay based on binding of NR-ceramide.

Having the identified hits from CERT-transfer assay, we were interested in further understanding the relationship between the CERT-transfer inhibitory activity and CERT-binding affinity. With this goal in mind, we have developed a homogenous competitive assay based on the binding of fluorescently-labeled ceramide to CERT-protein (**Fig S6**). It's well known that Nile Red is hydrophobic in nature and its fluorescence is significantly enhanced in hydrophobic environment than in hydrophilic one. Based on this knowledge, we have designed a fluorescently-labeled probe in which the Nile Red-tag was incorporated into the ceramide fatty acid moiety. The NR-ceramide probe displayed high fluorescence in the bound and low fluorescence in non-bound form with excellent signal-to-noise ratio (28-fold, **Fig S7.A**). Equilibration takes ~1h of incubation at 30°C. Furthermore, this assay is a simple mix-and read format and uses only low amounts of fluorophore (1µM) and CERT-protein (12.5µg/assay).

The assay was performed in non-binding black-bottom 96-well microplate (Greiner Bio-One). To each well was added 1µL HPA12 (final concentration 2.5µM) or DMSO, followed by 98 µL of the CERT aliquot (12.5µg/well). After the mixture was incubated for 40 min at 30°C, 1µL NR-ceramide (final concentration 1µM) was added. Controls were triplicates of 10mM Tris.HCl buffer (pH 7.4, 250 mM sucrose) with 2µL DMSO, CERT aliquot (12.5µg/well) with 2µL DMSO, and 1µL NR-ceramide (final concentration 1µM) in 10mM Tris.HCl buffer (pH 7.4, 250 mM sucrose) with 1µL DMSO. The plate was then sealed with transparent Thermowell seal tape and placed in the microplate reader (LABTECH) at 30°C with shaking. The fluorescence was measured from the top over 80min (number of cycles 35, delay 70 sec) with excitation filter 540 nm and emission filter 640 nm. The competitive CERT-binding activity was calculated using the formula: $I_x = I(\grave{A}_2\text{-}Y)/I_0(\grave{A}_1\text{-}X)$, where $\grave{A}_1$ is the average of the florescence of the control of NR-ceramide in Tris.HCl buffer, X is the average of the fluorescence of the buffer alone, $\grave{A}_2$ is the fluorescence average of the assay mixture, Y is the fluorescence average of the CERT aliquot with inhibitor/DMSO (**Fig S7.B**).

The ability to differentiate between HPA-12 (R,S) and (R,R) diastereomers is shown in **Fig S8A**. It is however lower than the differentiation in the transfer assay (**Fig S8B**).

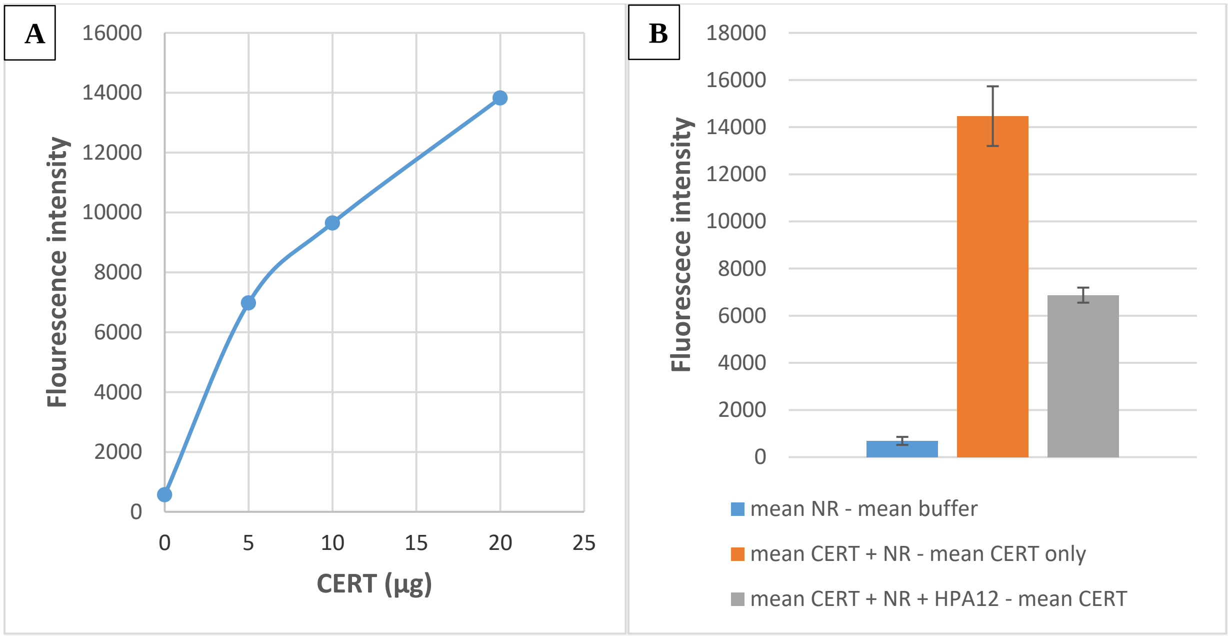

Figure S7. CERT competition assay based on Nile Red-labelled ceramide. **A.** Binding activity of NR-ceramide (1µM) to different concentration of CERT protein. **B.** Inhibition of NR-ceramide binding in presence of HPA12 (2.5µM).

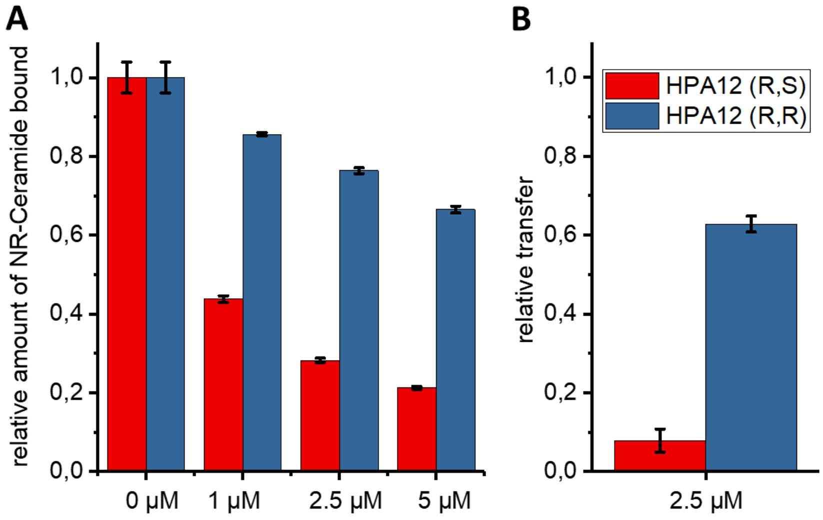

Figure S8. Differentiation between HPA-12 (R,S) (red columns) and HPA-12 (R,R) (blue columns) in different in vitro assays. **A** Binding in competition to NR-Cer **B** Transfer assay activity in comparison to untreated probe.

3.2 High throughput screening for CERT-activity and Data analysis

In order to validate the performance of developed assay in high-throughput screening format, the hits from ‘confirmed-hit group’ (45 hits) were screened for their competitive CERT-binding activity. The assay was performed as mentioned in the previous sub-section **3.1**. Briefly, 1µL tested compound (final concentration 1µM, 2.5µM, 5µM, 10µM) or DMSO for control, and 98 µL CERT stock solution were sequentially added into a non-binding 96-well microplate. The plate was sealed and incubated at 30°C for 40 min, before 1µL NR-ceramide (final concentration 1µM) was added. Controls per plate were triplicates of 10mM Tris.HCl buffer (pH 7.4, 250 mM sucrose) with 2µL DMSO, CERT aliquot (12.5µg/well) with 2µL DMSO, 1µL NR-ceramide (final concentration 1µM) in 10mM Tris.HCl buffer (pH 7.4, 250 mM sucrose), and HPA12 as a reference inhibitor (final concentration of 1µM, and 2.5µM, 5µM). The plate was sealed with transparent Thermowell seal tape and the assay was allowed to proceed as described above using excitation filter 540 nm and emission filter 640 nm. The competitive CERT-binding activity was calculated using the formula: $I_x = I(\grave{A}_2\text{-}Y)/I_0(\grave{A}_1\text{-}X)$, where $\grave{A}_1$ is the average of the florescence of the control 1µL NR-ceramide in Tris.HCl buffer, X is the average of the fluorescence of the buffer alone, $\grave{A}_2$ is the average of the fluorescence of assay mixture treated with tested compound or DMSO, Y is the average of the fluorescence of CERT aliquot with inhibitor/DMSO.

4. Microscale Thermophoresis (MST) assays

4.1 Labeling of the START domain with a fluorophore dye

In order to perform MST assays, the START domain was coupled with a commercially available fluorophore dye (NT647 (NanoTemper Technologies, Munich, Germany)) following standard procedures provided in the manufacturer's protocol. Briefly, 10 µM of the START domain was labeled with 30 µM of the fluorophore in NHS-LB buffer kept in the dark for 30min. Any excess of the dye was removed via size exclusion chromatography on a Sephadex G25 column (GE Healthcare). The concentration of the labeled protein was determined by UV-VIS spectrophotometry at 280 nm (Varian Cary 50).

4.2 Preparation of the dilution series and MST assay

Dilution series of small-molecule ligands were prepared from stock solutions in DMSO. In case of 6B11 and 17C9, ligands were then diluted into MST buffer (50 mM Na-phosphate, 150 mM NaCl, 2.5 mM DTT, 0.05% Tween-20) to a concentration 2-fold higher than the maximum concentration used in the assay. The concentration of DMSO was kept constant in all dilutions. 16 dilutions of the ligands were prepared in micro reaction tubes by successive 1:1 dilutions with MST buffer. In case of HPA12, 8E8, and 20D5, a pre-dilution approach was used to circumvent solubility problems. To this end, first, a dilution series in pure DMSO was generated. The individual dilutions were then further diluted 20-fold with MST buffer to a ligand concentration 2-fold higher than its final concentration. As a final step, 10 µL of the fluorescently labeled START domain was added to all reaction tubes containing 10 µL of ligand solution. The final concentration of START domain was 50 nM. After mixing, 2-3 µL of the reaction mixture were filled into standard capillaries (Nanotemper Technologies).

4.3 MST measurements

MST measurements were performed with a Monolith NT.115 instrument (Nanotemper Technologies). All ligands were measured as triplicates. 40% MST excitation power was set and, depending on the labeled protein concentration used in the assay, between 5% to 20% of LED power was used to obtain an optimized MST signal. By plotting the normalized fluorescence (ΔFnorm) against the ligand concentrations after 7 s or in some cases 5 s of thermophoresis the

binding curves for quantification of the KD values were obtained for each experiment. Data processing was performed within MO Affinity Analysis software (NanoTemper Technologies). Data points were fitted to a one-site binding model according to the following expression:

$$F_{norm} = \left(1 - \left(C_P + C_L + K_D - \sqrt{\frac{(C_P + C_L + K_D)^2 - 2C_P * C_L}{2C_P}}\right)\right) * F_{norm,notbound} + \left(C_P + C_L + K_D - \sqrt{\frac{(C_P + C_L + K_D)^2 - 2C_P * C_L}{2C_P}}\right) * F_{norm,bound}$$

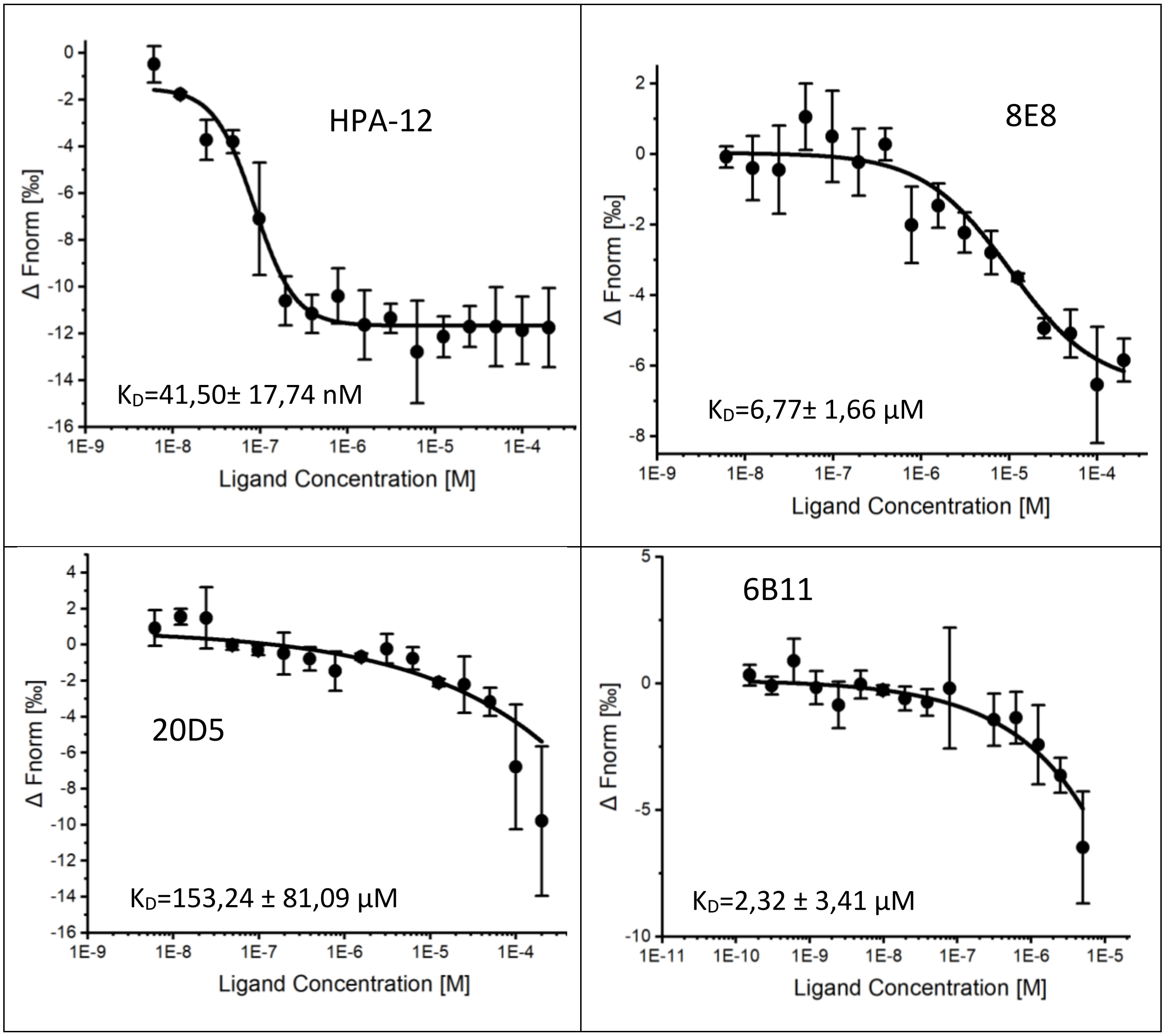

Figure S9. Binding curves to the START domain derived from MST experiments.

5. Cellular experiments

5.1 Cellular Localization of BODIPY-Ceramide

HeLa cells were seeded in 8 well chamber slides (ibidi µ-Slide 8 Well) in RPMI 1640 medium supplemented with 10% (v/v) heat-inactivated FCS, 1 mM sodium pyruvate and 2 mM L-glutamine at 37 °C and 5% (v/v) CO_2 in a humidified incubator. The day after, cells were incubated with inhibitors for 2 h at a concentration of 10 µM or left untreated by replacing the medium with fresh medium supplemented with the respective inhibitors. After 2 h of incubation, BODIPY FL C_5-ceramide (Thermo Fisher) was added to a final concentration of 0,25 µM and imaging was immediately done using an LSM 780 laser scanning confocal microscope (Carl Zeiss). No additional wash steps were performed. After 1 h of incubation, DNA was counterstained using Hoechst 33342. For quantification of BODIPY-Ceramide trafficking, cells were transfected with pECFP-GalT the day before inhibitor incubation for the expression of an eCFP fusion of the human galactosyltransferase (GalT; amino acids 1-60), which localizes at the Golgi apparatus. After incubation with the respective inhibitors (see above), cells were imaged at 30 min post addition of BODIPY FL C_5-ceramide. Mean BODIPY intensities of regions of interest surrounding eCFP-GalT-positive Golgi stacks were measured (40 cells per biological replicate, 4 biological replicates in total) using ImageJ software (Rasband, W.S., ImageJ, U. S. National Institutes of Health, Bethesda, Maryland, USA, https://imagej.nih.gov/ij/, 1997-2018) and divided by the total BODIPY intensity detected in the field of view. Quantification (**Fig 10B**) is expressed as normalized intensity ratio (GA/total).

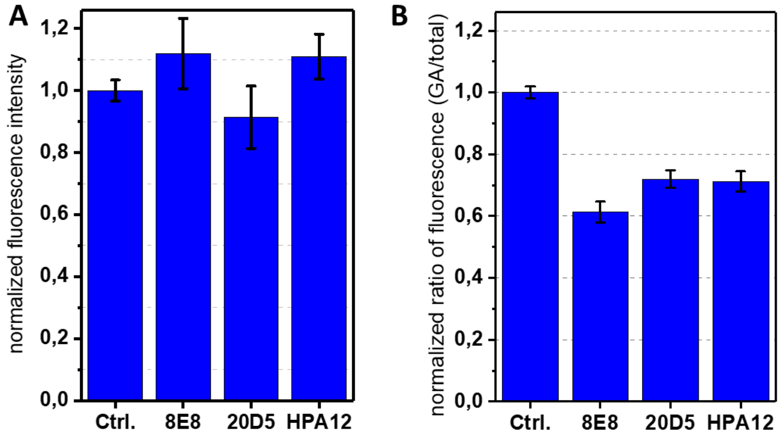

Figure S10. **A** Uptake of BODIPY FL C_5-ceramide to HeLa cells assessed by normalized fluorescence intensity **B** Distribution of BODIPY FL C_5-ceramide by normalized fluorescence (Golgi Apparatus/total).

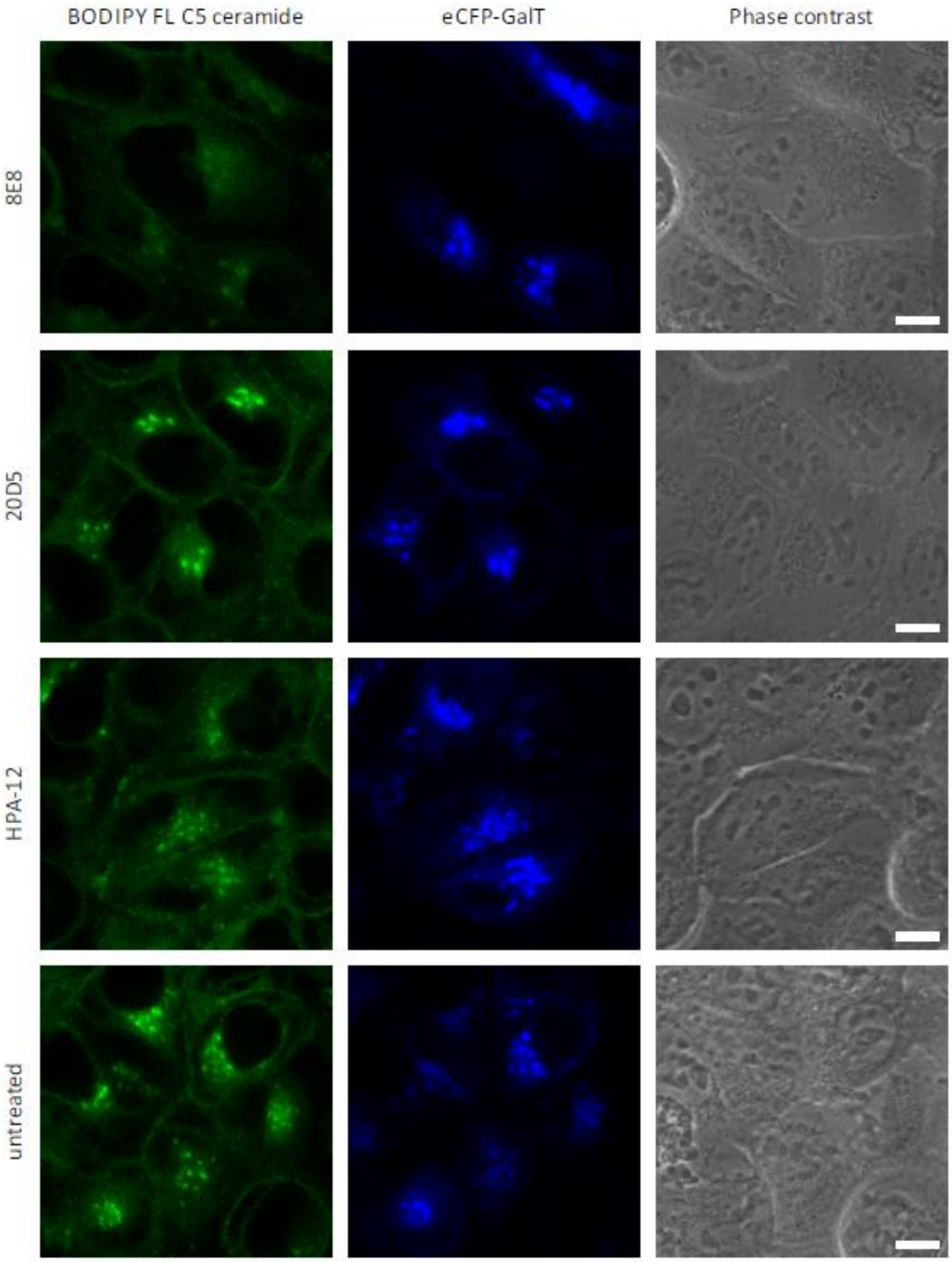

Figure S11. BODIPY-Ceramide trafficking. Confocal images showing distribution of BODIPY FL C_5-ceramide (green) and Golgi apparatus costaining (eCFP-GalT; blue) in HeLa cells pretreated with 10 µM of respective inhibitors or left untreated. Figures show representative images (n = 4 biological replicates with 40 cells per biological replicate); scale bars, 10 µm.

5.2 Cellular experiment for lipidomic analysis

Hela cells were cultured in six well plates (177000 cells per well) under standard growth conditions (DMEM with 4mM L-glutamine, 10% FBS, 1% Penicillin-Streptomycin; CO_2 incubator with 37°C, 5% CO_2). After incubation for 28h, the cells were treated with inhibitor (HPA12, Lomitapide, Fluralaner, Clevidipine butyrate and Eltrombopag) or 0.5% DMSO (control) and were incubated for additional 42h. The experiment was performed in triplicates with three different concentrations from the inhibitors (final concentrations: 1μM, 2.5μM and 5μM). After incubation, the adherent cells were washed twice with ice-cold PBS and harvested by treatment with trypsin. Cells were counted using a Countess II cell counter (Invitrogen) and adjusted to 500.000 cells per sample. Each sample was then treated as described below.

6. Details for experimental lipidomics

Cells were subjected to lipid extraction using 1.5 mL methanol/chloroform (2:1, v:v) as described [4]. The extraction solvent contained C17-ceramide (Cer17) and C16-d_{31}-sphingomyelin (d_{31}-SM16) (both Avanti Polar Lipids, Alabaster, USA) as internal standards. Chromatographic separations were achieved on a 1260 Infinity HPLC (Agilent Technologies, Waldbronn, Germany) equipped with a Poroshell 120 EC-C8 column (3.0 × 150 mm, 2.7 µm; Agilent Technologies). A mobile phase system consisting of water (solvent A) and acetonitrile/methanol (1:1, v:v; solvent B), both acidified with 0.1% formic acid, was used for gradient elution at an initial composition of 40:60 (A:B, v:v) and a flow rate of 0.5 mL/min. MS/MS analyses were carried out using a 6490 triple-quadrupole mass spectrometer (Agilent Technologies) operating in the positive electrospray ionization mode (ESI+). The following ion source parameters were set: sheath gas temperature, 375 °C; sheath gas flow, 12 L/min of nitrogen; nebulizer pressure, 30 psi; drying gas temperature, 200 °C; drying gas flow, 15 L/min of nitrogen; capillary voltage, 4000 V; nozzle voltage, 1500 V; iFunnel high pressure RF voltage, 150 V and iFunnel low pressure RF voltage, 60 V. The following mass transitions were recorded (collision energies of 25 eV for all transitions): ceramides: *m/z* 520.5 → 264.3 for Cer16, *m/z* 534.5 → 264.3 for Cer17, *m/z* 548.5 → 264.3 for Cer18, *m/z* 576.6 → 264.3 for Cer20, *m/z* 604.6 → 264.3 for Cer22, *m/z* 630.6 → 264.3 for Cer24:1 and *m/z* 632.6 → 264.3 for Cer24; sphingomyelins: *m/z* 703.6 → 184.1 for SM16, *m/z* 731.6 → 184.1 for SM18, *m/z* 734.8 → 184.1 for d_{31}-SM16, *m/z* 759.6 → 184.1 for SM20, *m/z* 787.7 → 184.1 for SM22, *m/z* 813.7 → 184.1 for SM24:1 and *m/z* 815.7 → 184.1 for SM24. Quantification was performed

with MassHunter Software (Agilent Technologies). Significance of differences between two samples was calculated by student's t-test. Asterisks indicate significant differences between treated and untreated samples: * $p<0.05$; ** $p<0.01$; *** $p<0.005$.

Table S1. Sphingolipid content of treated cells.

			pmol/sample																
	**HU Berlin ID**		***S1P***	***Sph***	***Cer16***	***Cer18***	***Cer20***	***Cer22***	***Cer24***	***Cer24:1***	***Cer total***	***SM16***	***SM18***	***SM20***	***SM22***	***SM24***	***SM24:1***	***SM total***	*ratio total SM/Cer*
1			1,34	4,45	44,57	3,14	0,86	8,03	27,02	65,69	149,31	1834,16	53,71	18,84	123,93	200,35	875,29	3106,27	20,8
2	DMSO		1,13	5,06	47,12	3,26	0,90	8,41	26,99	68,06	154,74	1870,93	53,08	18,82	121,65	193,75	874,35	3132,58	20,2
3			1,01	4,99	48,14	3,95	1,98	9,95	29,01	67,07	160,11	1960,33	55,21	18,79	126,15	205,74	933,37	3299,59	20,6
4			1,34	3,57	45,94	3,38	1,18	8,53	25,58	68,53	153,13	1695,91	47,55	15,20	91,42	147,79	708,14	2706,02	17,7
5		1 µM	1,27	3,87	48,20	3,35	1,15	8,47	25,47	64,01	150,65	1668,53	44,39	14,34	86,76	134,40	660,22	2608,64	17,3
6			1,52	3,94	44,92	2,85	0,88	7,59	26,70	60,79	143,74	1609,69	46,43	14,30	85,97	140,17	659,25	2555,82	17,8
7			1,60	2,13	45,72	2,82	0,99	8,93	29,39	64,09	151,94	1489,44	53,85	20,27	75,95	129,06	581,92	2350,50	15,5
8	HPA-12	2.5 µM	1,73	3,27	45,34	3,05	0,90	7,27	22,20	55,48	134,23	1440,97	43,84	13,88	76,48	114,60	555,40	2245,18	16,7
9			1,58	2,40	45,94	2,77	0,94	8,93	31,70	66,75	157,03	1448,69	45,37	14,55	75,20	121,10	524,32	2229,23	14,2
10			1,43	3,33	43,83	2,50	0,74	6,88	20,93	53,37	128,26	1306,31	41,78	12,76	68,87	101,53	471,40	2002,66	15,6
11		5 µM	1,97	3,81	46,26	2,62	0,81	6,54	21,64	55,90	133,78	1249,58	39,06	12,48	67,07	101,48	487,46	1957,13	14,6
12			1,45	3,47	43,34	2,51	0,73	6,85	23,60	57,30	134,34	1356,77	41,13	12,54	70,68	113,95	521,22	2116,29	15,8
13			1,12	3,81	57,09	6,95	2,84	13,35	30,90	68,22	179,34	1825,61	59,37	20,60	115,26	173,05	817,85	3011,74	16,8
14		1 µM	1,00	3,57	66,02	7,66	2,25	14,47	34,96	73,09	198,45	1823,48	58,79	20,35	124,65	183,46	842,40	3053,13	15,4
15			0,85	3,42	60,15	6,67	1,77	13,05	30,93	65,48	178,06	1739,12	52,57	17,81	105,99	158,17	760,17	2833,83	15,9
16			0,75	3,63	74,51	8,90	2,09	14,83	32,62	74,15	207,10	1630,84	53,11	19,44	103,91	157,53	731,59	2696,43	13,0
17	8E8	2.5 µM	1,06	3,39	82,98	10,02	2,26	17,07	36,00	79,98	228,30	1823,72	57,73	21,11	113,22	168,83	797,90	2982,51	13,1
18			1,09	3,50	79,41	9,50	2,11	15,69	36,33	77,75	220,79	1666,50	55,13	18,70	105,74	156,81	722,25	2725,14	12,3
19			0,37	3,46	89,79	7,26	1,69	12,62	22,56	66,19	200,11	450,01	25,94	8,48	36,80	50,34	200,60	772,16	3,9
20		5 µM	0,42	3,80	129,48	11,79	2,84	18,55	31,76	87,40	281,82	691,68	35,37	11,39	52,48	70,92	298,06	1159,90	4,1
21			0,53	3,91	69,03	5,12	1,31	9,43	18,74	47,19	150,81	345,52	18,65	5,85	26,91	33,38	137,99	568,29	3,8
22			1,21	3,07	40,19	3,84	1,12	9,12	37,27	40,44	131,97	1625,68	54,98	18,10	110,98	197,09	621,75	2628,58	19,9
23		1 µM	1,34	3,74	45,48	6,03	2,67	11,46	40,67	46,05	152,37	1717,57	59,04	18,45	111,56	206,21	662,66	2775,49	18,2
24			1,10	3,46	39,73	4,12	1,37	9,24	36,50	40,74	131,71	1686,01	56,68	17,14	111,04	199,98	645,68	2716,53	20,6
25			1,21	3,04	40,44	4,61	1,33	9,74	35,83	37,47	129,42	1311,55	48,19	16,11	98,76	179,29	553,18	2207,08	17,1
26	20D5	2.5 µM	1,29	3,17	43,49	4,98	1,40	10,99	40,22	43,87	144,94	1447,18	50,02	17,46	109,23	202,61	603,33	2429,82	16,8
27			1,29	2,91	36,97	3,95	1,09	8,69	34,92	37,54	123,16	1450,27	51,79	16,39	102,97	193,22	587,13	2401,78	19,5
28			1,32	3,25	48,26	5,41	1,35	9,51	33,23	35,26	133,01	1128,33	43,19	13,45	74,74	132,70	419,73	1812,15	13,6
29		5 µM	1,22	3,29	50,29	5,92	1,41	10,83	39,15	38,24	145,85	1262,96	47,39	15,19	85,38	158,23	481,61	2050,77	14,1
30			1,19	4,27	47,46	5,34	1,29	9,59	38,78	35,25	137,71	1233,63	44,90	13,75	82,05	155,99	461,72	1992,04	14,5
31			1,30	3,14	30,33	2,31	0,75	5,21	18,20	35,42	92,23	1664,57	49,35	16,44	97,77	148,40	734,55	2711,09	29,4
32		1 µM	1,12	2,93	35,47	3,21	0,82	6,64	21,95	41,58	109,68	1753,19	48,17	17,01	109,23	164,36	781,78	2873,72	26,2
33			1,30	2,69	32,28	3,62	1,87	6,96	20,24	36,60	101,56	1827,88	50,13	16,12	104,57	157,65	776,18	2932,53	28,9
34			1,11	3,54	31,11	2,90	1,10	6,09	19,85	35,16	96,21	1754,78	52,01	16,63	104,73	164,67	752,78	2845,61	29,6
35	6B11	2.5 µM	1,11	2,83	34,56	3,36	1,04	6,54	19,17	36,98	101,65	1844,30	48,63	16,52	101,64	154,96	753,00	2919,05	28,7
36			1,10	2,99	32,33	2,45	0,67	4,24	13,13	34,53	87,36	1802,54	47,35	14,33	87,68	126,92	701,62	2780,44	31,8
37			1,11	2,83	29,66	2,65	0,74	5,38	17,46	36,92	92,81	1762,30	53,21	17,74	109,54	166,35	783,66	2892,80	31,2
38		5 µM	1,25	2,13	34,26	3,21	0,90	6,67	20,37	40,53	105,93	1782,59	52,89	18,28	111,84	172,87	796,71	2935,19	27,7
39			1,08	3,03	31,66	2,71	0,79	6,09	20,18	39,54	100,96	1722,69	50,26	16,92	106,91	164,46	771,15	2832,39	28,1
40			1,15	2,70	29,98	2,62	0,80	6,14	19,49	38,79	97,82	1737,34	50,83	17,12	108,33	172,22	788,48	2874,32	29,4
41		1 µM	1,32	3,11	34,40	3,09	0,85	6,25	18,45	39,87	102,91	1866,45	50,55	16,70	106,97	163,43	772,38	2976,47	28,9
42			1,00	4,01	31,42	3,68	3,45	5,55	16,72	35,52	96,34	1725,36	45,45	15,18	93,72	147,49	712,32	2739,52	28,4
43			1,23	4,57	32,36	3,43	2,29	6,89	19,23	39,53	103,73	1702,42	47,65	15,77	97,64	148,54	706,74	2718,76	26,2
44	17C9	2.5 µM	1,05	3,32	33,19	3,27	1,23	6,59	20,44	38,37	103,08	1763,99	51,14	16,69	104,36	158,37	726,62	2821,17	27,4
45			1,02	3,82	28,45	2,51	0,90	5,14	16,88	33,82	87,70	1597,48	44,81	14,30	90,89	140,98	659,70	2548,15	29,1
46			0,97	3,92	29,49	2,38	0,79	4,97	18,36	34,90	90,90	1510,97	45,08	14,34	86,79	143,47	606,54	2407,20	26,5
47		5 µM	0,93	3,79	35,89	3,05	0,94	6,52	21,85	40,79	109,04	1730,88	52,99	17,35	107,23	171,51	703,57	2783,54	25,5
48			0,94	3,72	33,40	2,58	0,73	6,08	21,93	38,77	103,49	1785,73	49,27	16,52	102,55	164,74	702,64	2821,45	27,3

7. Docking, molecular dynamics and binding free energy calculations

Docking. The X-ray crystal structure of CERT START bound with C16 ceramide (PDB: 2E3P) was used for docking [2]. After removal of the ceramide, Autodock Vina [5] was used to dock the ligands to the ceramide-binding pocket. The ceramide-binding residues were set flexible during docking. Three top-ranked binding poses of each ligand were subject to molecular dynamics (MD) simulations to assess their stability.

Molecular dynamics. Each CERT-ligand complex was solvated in a periodic box of explicit water and neutralized with 0.15 M of Na^+ and Cl^- ions. Effective point charges of the ligands were obtained by RESP fitting [6] of the electrostatic potentials calculated with the HF/6-31G* basis set using Gaussian 09 [7]. The Amber 99SB-ildn [8] and GAFF [9] force fields were used for the protein and the ligands, respectively. The TIP3P [10] and the Joung-Cheatham models [11] were used for the water and the ions, respectively. After energy minimization, all-atom MD simulations were carried out using Gromacs 2018 [12] patched with the PLUMED 2.3 plugin [13]. Each system was gradually heated to 310 K and pre-equilibrated during 10 ns of brute-force MD in the *NPT*-ensemble. The replica exchange with solute scaling (REST2) [14] technique was then employed to enhance the sampling with 48 replicas in the *NVT* ensemble. The protein and the ligands were considered as "solute" in the REST2 scheme–force constants of their van der Waals, electrostatic and dihedral terms were subject to scaling. The effective temperatures used for generating the REST2 scaling factors ranged from 310 K to 700 K, following a distribution calculated with the Patriksson-van der Spoel approach [15]. Exchange between replicas was attempted every 1000 simulation steps. This setup resulted in an average exchange probability of ~40% during 40 ns (× 48 replicas) of simulations. The first 5 ns were discarded for equilibration. Only the original unscaled replica (at 310 K effective temperature) was collected and analyzed.

Binding free energy calculations. The ligand binding free energies were calculated with MMPBSA.py [16] using the simulation trajectories. The interaction enthalpy and per-residue decomposition were calculated with the generalized Born model, whereas the entropic contribution was estimated by normal mode analysis.

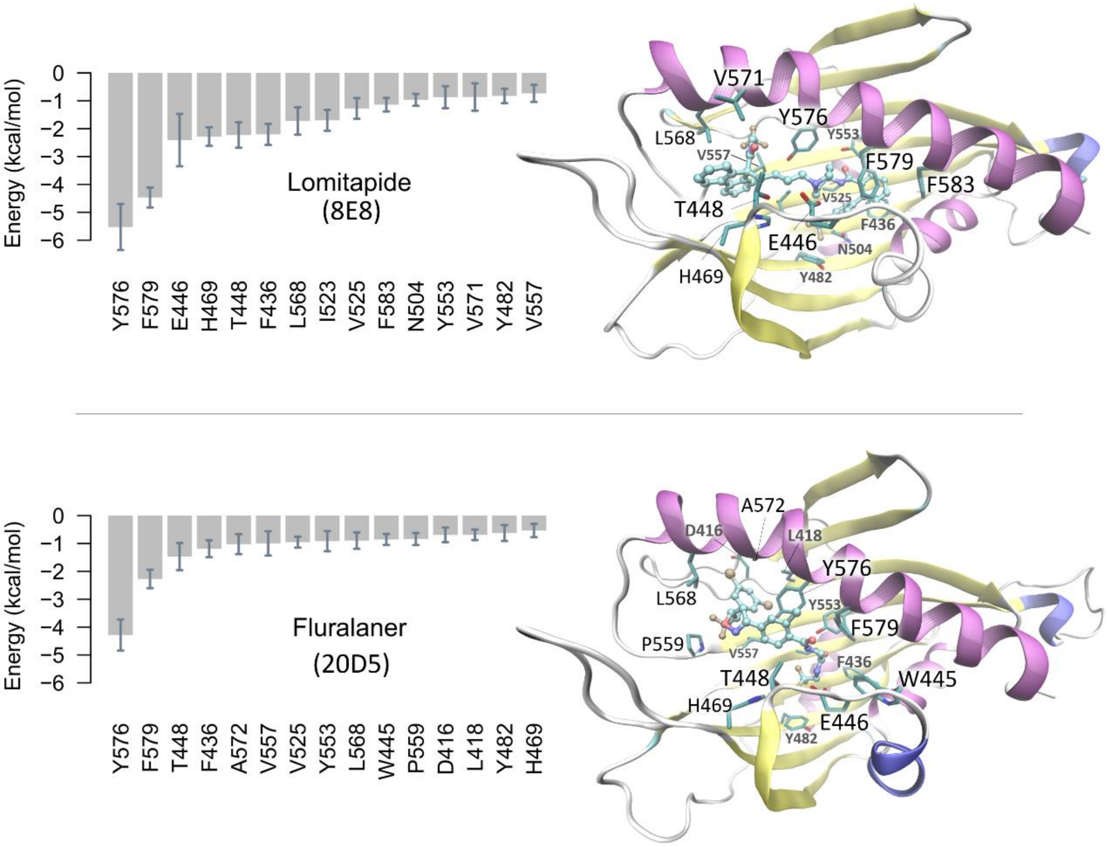

Figure S12. Key interactions and per-residue binding energy (enthalpy) decomposition. Error bars represent the standard deviations.

8. General methodology for organic syntheses

8.1 General considerations

Unless otherwise specified, all reactions were carried out in oven-dried (>120°C) glassware equipped with a magnetic stir bar and a rubber septum under a positive pressure of argon. Air- or moisture-sensitive reagents were transferred to the reaction vessel under positive pressure of argon via syringe. Air and/or moisture sensitive reactions were carried out in well dried glassware under an argon atmosphere with dry, freshly distilled solvents using standard syringe-cannula/septa techniques. Reactions were run at room temperature (20-25°C) unless otherwise noted in the experimental procedure, and reported reaction temperatures refer to the external temperatures measured for the bath in which the reaction vessel was immersed. Heating was obtained through the use of a silicone oil bath. For reactions run below room temperature, the term "-78°C" refers to a bath of acetone and dry ice, "-20°C" refers to a slurry of sodium chloride and ice-water bath, and "0°C" refers to an ice-water bath. Removal of residual solvents was accomplished by evacuation of the container for a period of 12-20 hours using a high vacuum line.

8.2 Reagents and solvents

All the commercially available reagents were purchased from Sigma-Aldrich, TCI, Fluka or Acros and used without further purification, unless otherwise specified. All the solvents were used after distillation by standard methods. The petroleum ether used throughout this study had a boiling range of 40–60°C.

8.3 Chromatography

The thin layer chromatography studies were performed on pre-coated silica gel 60-F_{254} on aluminum sheets (Merck KGaA) and spots were detected by UV illumination (254 nm), and/or spraying with 1.3% ninhydrin solution, ceric ammonium molybdate (Seebach reagent) solution (25 g $MoO_3 \cdot H_3PO_4 \cdot H_2O$, 10 g $Ce(SO_4)_2 \cdot 4H_2O$, 60 ml H_2SO_4 and 905 ml of H_2O) or $KMnO_4$ solution (1.5 g $KMnO_4$, 10 g K_2CO_3 and 1.25 ml 10% NaOH in 200 ml water) followed by heating. Preparative flash column chromatography was performed manually using glass columns of different size packed with Silica Gel 60M (0.04-0.063 mm) as stationary phase with indicated eluent systems in parenthesis following the description of purification. Solvent ratios for chromatography and R_f values are reported in v/v% ratios.

8.4 Spectroscopic Data

The structure of all synthesized compounds was confirmed with ^{1}H NMR, ^{13}C NMR, DEPT, ^{31}P NMR and MS analysis. ^{1}H, ^{13}C and ^{31}P NMR spectra were recorded on Bruker AVANCE II 300, AVANCED PX 300, AVANCE 400 and Bruker ADVANCE III 500 spectrometers (^{1}H at 300, 400 or 500 MHz, ^{13}C at 75.4, 101.2 or 125.7 MHz and ^{31}P at 161.9 or 202.4 Hz) as solutions in $CDCl_3$, CD_3OD or mixtures of those at 25 °C. Chemical shifts (δ) are reported in parts per million (ppm): multiplicities are indicated as s (singlet), d (doublet), t (triplet), q (quartet), m (multiplet) and br (broad). Chemical shifts are given in ppm with respect to TMS as an external standard, (^{1}H, APT, ^{13}C, δ = 0.00) with calibration against the residual solvent signal or 85% H_3PO_4 (^{31}P, δ= 0.00) as external standard. The coupling constants *J* are given in Hz.

8.5 Mass spectrometry

Mass spectrometry (MS) experiments were recorded on an AGILENT 6120 UPLC–MS system consisting of an SQD (single quadrupole detector) mass spectrometer equipped with an electrospray ionization interface (ESI) in the positive and negative ion detection modes. The samples were separated on a Zorbax Eclipse Plus C18 column (particle size 1.8 μ m, 2.1 × 50 mm) using a UPLC pump at a flow rate of 0.8 ml per min with a ternary solvent system of MeOH-H_2O-HCOOH, methanol (99.9% MeOH: 0.1% HCOOH, v/v). The column was first equilibrated using a mixture of 95% mobile phase A and 5% mobile phase B, and then 10 μ l of the sample was injected. This was followed by a ramp gradient over 2 min to 95% phase B and 5% phase A, which remained until 7 min, followed by a ramp gradient back down to 95% solvent A and 5% solvent B for 1 min, and column equilibration with the same mixture for 1 min. The detection was performed in full scan mode and the major observable molecular ion and selected fragments and clusters have been reported.

9. Synthetic procedures and analytic data of compounds

9.1 Synthesis of 7-nitro-N-(undec-10-en-1-yl)benzo[*c*][1,2,5]oxadiazol-4-amine

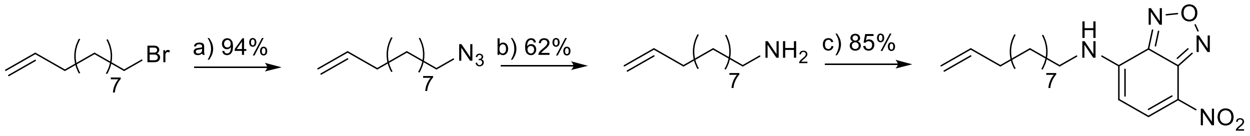

Scheme 1: synthesis of 7-nitro-N-(undec-10-en-1-yl)benzo[*c*][1,2,5]oxadiazol-4-amine (**3**). Reagents and conditions: a) NaN_3, DMF, rt,14h; b) $LiAlH_4$, THF, 0°C-rt, 1h; c) NBD-Cl, DIPEA, MeOH, 0°C-rt, 16h.

Synthesis of 11-azidoundec-1-ene (1)

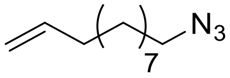

To a stirred solution of 11-Bromoundec-1-ene (1.4 g, 6.0 mmol, 1eq) in dry DMF (12 mL, 0.5 M) under an argon atmosphere was added (0.39 g, 6.3 mmol, 1.1 eq) of sodium azide (NaN_3). The solution was stirred for 14 h at room temperature. The obtained reaction mixture was added into 20 mL water and extracted with ethyl acetate (2 x 50 mL). The collected organic layer was washed with brine solution and then dried over anhydrous Na_2SO_4. Solvent was evaporated under reduced pressure. Purification of the obtained residue was done by column chromatography on silica gel using ethyl acetate and cyclohexane as eluents (5-15% ethyl acetate in cyclohexane) to furnish the desired product **1** as a colorless oil [17].

Yield: 1.10 g (94%). $R_{f.}$ 0.67 (ethyl acetate:cyclohexane 5:95, visualized with $KMnO_4$).

^{1}H NMR ($CDCl_3$, 500 MHz, ppm): δ 5.81 (ddt, *J*= 6.7, 10.2, 16.9 Hz, 1H, =CH), 4.91-5.01 (m, 2H, =CH2), 3.25 (t, *J*= 7.0 Hz, 2H, CH_2N3), 2.04 (m, 2H, CH_2CH=), 1.56-1.63 (m, 2H, CH_2), 1.27-1.40 (m, 12H, 6×CH_2).

^{13}C NMR ($CDCl_3$, 125 MHz, ppm): δ 139.18 (=CH), 114.14(=CH_2), 51.50 (CH_2N), 33.79 (CH_2-CH=), 29.42 (CH_2), 29.37 (CH_2), 29.14 (CH_2), 29.08 (CH_2), 28.91 (CH_2), 28.84 (CH_2), 26.72 (CH_2).

Synthesis of undec-10-en-1-amine (2)

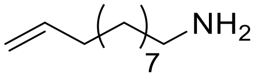

To an ice cooled solution of **1** (1.55 g, 7.94 mmol, 1eq) in 80 mL THF (0.1M), Lithiumaluminumhydride solution 2.4 M in THF (4 mL, 9.5 mmol, 1.2 eq) was added dropwise

over 10 min. After The reaction mixture was stirred for 1h at 0°C, 200 ml ice-cooled 1M HCl solution was added dropwise to quench the reaction. The obtained mixture was extracted with ethyl acetate (2 x 100 mL). The collected organic layers were washed with saturated $NaHCO_3$ solution, brine solution, and dried over anhydrous Na_2SO_4. Afterwards, the solvent was evaporated under reduced pressure to obtain the desired product **2** as a colourless oil. The product was used in the next step without any further purification [17].

Yield: 0.84 g (62 %). R_f: 0.12 (1:9 Ethyl acetate:Cyclohexane, visualized with 1.3% ninhydrin).
^{1}H-NMR ($CDCl_3$, 500 MHz, ppm): δ = 5.79 (ddt, *J*= 6.7, 10.2, 16.9 Hz, 1H, =CH), 4.84-5.01 (m, 2H, =CH_2), 4.02 (br. M, 2H, NH_2) 2.54-2.77 (br. m, 2H, C*H*$_2$$NH_2$), 1.92-2.09 (m, 2H, C*H*$_2$CH=), 1.47-159 (m, 2H, CH_2), 1.15-1.43 (m, 12H, 12×CH_2).
^{13}C-NMR ($CDCl_3$, 126 MHz, ppm): δ 26.80 (CH_2), 28.92 (CH_2), 29.10 (CH_2), 29,32 (CH_2), 29.42 (CH_2), 29.62 (CH_2), 33.79 (CH_2CH=), 41.32 (CH_2NH_2), 114.12 (=CH_2), 139.18 (=CH).
ESI-MS: *m/z* calcd for $C_{11}H_{24}N$ $(M+H)^+$ 170.19; observed 170.20.

Synthesis of 7-nitro-N-(undec-10-enyl)benzo[c][1,2,5]oxadiazol-4-amine (3)

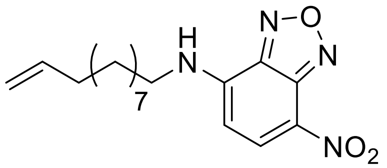

To an ice cooled stirred solution of 7-nitro-N-(undec-10-enyl)benzo[c][1,2,5]oxadiazol-4-amine (NBD-Cl) (1.4 g, 7 mmol, 1eq) in 70 mL methanol was added *N*,*N*-diisopropylethylamine (DIPEA) (6.5 mL, 5.5 eq), followed by a dropwise addition of a solution of compound **(2)** (1.2 gm, 7 mmol, 1eq) in 70 mL methanol. The reaction mixture was stirred at room temperature overnight under light exclusion. The solvent was evaporated under reduced pressure and the obtained residue was purified on silica gel by column chromatography using cyclohexane and ethyl acetate as eluents (15-25% ethyl acetate in cyclohexane) to give the desired product **3** as red solid [17].

Yield: 2.0 g (85%). R_f 0.55 (cyclohexane/EtOAc 7:3, visualized with UV illumination at 365 nm).
^{1}H NMR ($CDCl_3$, 300 MHz, ppm): δ 8.50 (d, *J*= 8.6 Hz, 1H, CH_{ar}), 6.22 (br. s, 1H, NH), 6.17 (d, *J*= 8.7 Hz, 1H, CH_{ar}), 5.81 (ddt, *J*= 6.7, 10.2, 16.9 Hz, 1H, =CH), 4.87-5.06 (m, 2H, =CH_2), 3.49

(dd, *J*= 7.1, 13.0 Hz, 2H, C*H*NH), 1.97-2.11 (m, 2H, CH_2CH=), 1.81 (dt,*J*=7.3, 14,8 Hz, 2H, CH_2), 1.25-1.52 (m, 12H, 12×CH_2).

^{13}C NMR ($CDCl_3$, 75 MHz, ppm): δ 148.97 (Car), 139.89 (C_{ar}), 136.11 (=CH, CH_{ar}), 131.15 (C_{ar}), 120.29 (C_{ar}), 114.22 (=CH_2), 101.09 (CH_{ar}) 44.13 (CHNH), 33.90 (CH_2CH=), 29.53 (CH_2), 29.49 (CH_2), 29.33 (CH_2), 29.18 (CH_2), 29.00 (CH_2), 28.68 (CH_2), 27.07 (CH_2).

ESI-MS: *m/z* calcd for $C_{17}H_{24}N_4O_3$ $(M+H)^+$ 333.19; observed 333.20.

9.2 Synthesis of NBD-Ceramide-C16 (12)

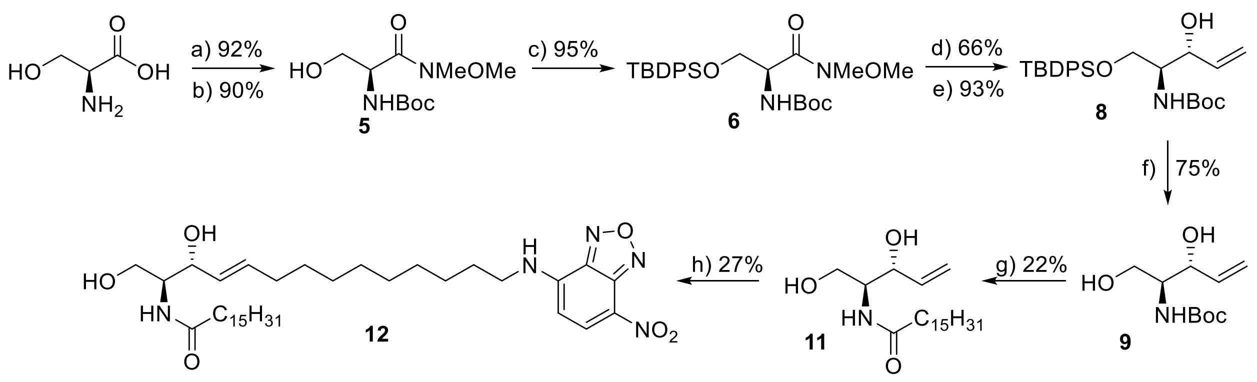

Scheme 2: NBD-Ceramide-C16. Reagents and conditions: a) Boc_2O, 1M NaOH, dioxane, -5°C to r.t., 4h; b) Me(MeO)NH.HCl, EDCI.HCl, NMM, DCM, -15°C, 2h; c) TBDPSCl, imidazole, DMF, 16h; d) vinylmagnesium bromide (1M in THF), THF, -20°C-r.t., 4h; e) TBLAH, EtOH, -78°C, 2h; f) TBAF,THF, reflux, 3h; g) (i) TFA, DCM, 0°C-rt, 3h, (ii) $C_{15}H_{31}COCl$, DIPEA, DCM, 0°C-rt, 2h; h) **3**, Hoveyda-Grubbs catalyst 2nd Generation, rt, 16h.

tert-butyl ((2*S*,3*R*)-1,3-dihydroxypent-4-en-2-yl)carbamate (9)

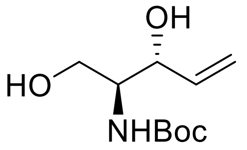

Compound (**8**) was prepared according to a reported method [17]. To a stirred solution of compound **(8)** (5 g, 10.97 mmol, 1 eq) in dry THF (60 mL) under an argon atmosphere, a solution of n-tetrabutylammonium fluoride (TBAF) (1M solution in THF 16.45 mL, 16.46 mmol, 1.5 eq) was added dropwise over 15 min at room temperature. Afterwards, the reaction mixture was refluxed for 3h (checked by TLC analysis for complete conversion of the starting material). After removing the solvent under reduced pressure, the residue was dissolved in dichloromethane (150ml), then washed with water (150 mL). the layers were separated, and the aqueous layer was

extracted with dichloromethane (3×100 mL). The collected organic layers were washed with saturated $NaHCO_3$ solution (200 mL), brine (200 mL), dried over anhydrous Na_2SO_4, filtered and concentrated under reduced pressure. The obtained residue was purified by column chromatography on silica gel using methanol and dichloromethane as eluents (from 5-7% methanol in dichloromethane) to give compound **(9)** as a colourless oil [17].

Yield: 1.78 g (75%). R_f: 0.36 (methanol:dichloromethan 5:95, visualized with KMnO4).

^{1}H NMR ($CDCl_3$, 500 MHz, ppm): δ 5.93 (ddd, J = 5.3, 10.6, 17.2 Hz, 1H, =CH), 5.39 (dt, J = 1.5, 17.2 Hz, =C*H*H), 5.18-5.33 (dt J= 1.5, 17.2, 2H, =CH*H*+N*H*), 4.38 (br. m, 1H, C*H*OH), 3.95 (ddd, J =3.6, 11.4, 24.5 Hz, 1H, C*H*HO), 3.71(dd, *J* = 3.7, 11.3 Hz, 1H, CH*H*O), 3.64 (m, 1H, C*H*NH), 2.99 (br. s, 1H, OH) ,2.71(br. s, 1H, OH), 1.45 (s, 9H, $(CH_3)_3CO$).

^{13}C NMR ($CDCl_3$, 126 MHz, ppm): δ 156.25 (NCOO), 137.29 (=CH), 116.69 (=CH_2), 75.93 (Me_3CO), 74.60 (CHOH), 62.68 (CH_2O), 55.01(CHNH), 27.48 ($(CH_3)_3CO$).

ESI-MS *m/z* calcd for $C_{10}H_{20}NO_4$ $[M+H]^+$: 218,13, found: 218.13

m/z calcd for $C_{10}H_{19}NO_4Na$ $[M+Na]^+$: 240.12 , found: 240.20

(2*S*,3*R*)-2-aminopent-4-ene-1,3-diol (10)

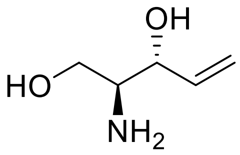

To a stirred solution of compound **(9)** (0.30 g, 1.38 mmol) in dichloromethane (7 mL, 0.1 M) was added 1.2 mL of trifluoroacetic acid (TFA) dropwise at 0°C. The reaction mixture was stirred for 30 min at the same temperature, then warmed gradually to room temperature and stirring was continued for an additional 2 h. TLC analysis showed complete deprotection of the starting material to a highly polar product which visualized as a brown spot with ninhydrin staining. The solvent was evaporated under reduced pressure and was dried under high vacuum for 2 days to get rid of all excess TFA. The crude product was used directly in the next step without any further purification [17].

***N*-((2*S*,3*R*)-1,3-dihydroxypent-4-en-2-yl)palmitamide (11)**

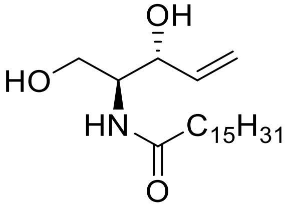

To a stirred solution mixture of compound (**10**) (300 mg, 2.56 mmol, 1eq) in dry dichloromethane (25 mL, 0.1M) and N,N-diisopropylethylamine (DIPEA) (1.34 mL, 7.68 mmol, 3 eq) at 0°C under an argon atmosphere was added a solution of palmitoyl chloride (855µL, 2.82mmol, 1.1 eq) in dichloromethane (27 mL, 0.1 M) dropwise over 10 minutes. Afterwards, the resulting reaction mixture was stirred for 20 min at the same temperature, then gradually warmed to the room temperature and stirred for an additional 2 h (as judged by TLC analysis for complete acylation of the starting material). Then, the reaction mixture was cooled to 0°C and quenched by addition of 1 M HCl dropwise until acidic pH. The obtained mixture was extracted with dichloromethane (4×50mL) and the collected organic layers were washed with $NaHCO_3$ solution, brine, dried over anhydrous Na_2SO_4, filtered and concentrated under reduced pressure. The crude product was purified by column chromatography on silica gel using ethyl acetate and cyclohexane as eluents (ethyl acetate:cyclohexane 7:3) to give the product (**11**) as white solid [17].

Yield: 200 mg (22%). R_f: 0.33 (Ethyl acetate 100%, visualized with $KMnO_4$).
^{1}H NMR (D_2O, 500 MHz, ppm): δ 5.88 (ddd, *J* = 16.9, 10.5, 6.4 Hz, 1H, =CH), 5.28 (dt, *J* = 17.1, 1.6 Hz, 1H, =C*H*H), 5.14 (dt, *J* = 10.5, 1.6 Hz, 1H, =CH*H*), 4.12 (tt, *J* = 6.5, 1.3 Hz, 1H, C*H*OH), 3.89 (dt, *J* = 7.0, 5.1 Hz, 1H, C*H*NH), 3.68 (td, *J* = 4.9, 1.6 Hz, 2H, CH_2OH), 2.21 (q, *J* = 7.5 Hz, 2H, CH_2CO), 1.60 (dt, *J* = 14.4, 5.3 Hz, 2H, CH_2), 1.34 – 1.27 (m, 24H, 12×CH_2), 0.89 (t, *J* = 6.8 Hz, 3H, CH_3).
^{13}C NMR (D_2O, 126 MHz, ppm): δ 169.45 (CONH), 139.22 (=CH), 116.41 (=CH_2), 73.47 (CHOH), 61.67 (CH_2OH), 56.21 (CHNH), 36.89(CH_2CONH), 32.73 (CH_2), 30.45 (CH_2), 30.41 (CH_2), 30.41(CH_2), 30.38 (CH_2), 30.37 (CH_2), 30.29 (CH_2), 30.15 (CH_2), 30.13 (CH_2), 29.99 (CH_2), 27.36 (CH_2), 26.69 (CH_2), 23.39 (CH_2), 14.09 (CH_3).
ESI-MS *m/z* calcd for $C_{21}H_{42}NO_3$ $[M+H]^+$: 356,3, observed: 356.3
m/z calcd for $C_{21}H_{41}NO_3Na$ $[M+Na]^+$: 378.2 , observed: 378.3

***N*-((2*S*,3*R*,*E*)-1,3-dihydroxy-14-((7-nitrobenzo[*c*][1,2,5]oxadiazol-4-yl)amino)tetradec-4-en-2-yl)palmitamide (12)**

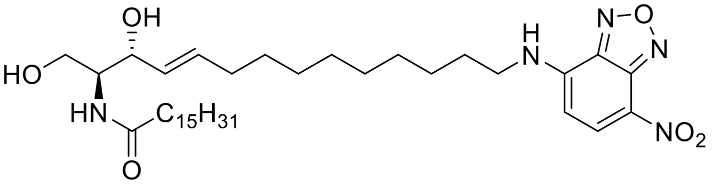

To a stirred solution of compound **(11)** (25 mg, 70.4 nmol, 1eq) in 2.5 mL dry dichloromethane (the starting material was dissolved in DCM by warming) at room temperature under an argon atmosphere was added compound **(3)** (81.8 mg, 0.25 mmol, 3.5 eq), followed by addition of Hoveyda-Grubbs catalyst 2nd Generation (3 mg, 7 mol%). After the reaction mixture was stirred overnight with the exclusion of light (as checked by TLC and UPLC analysis which indicated the complete conversion), the solvent was evaporated under reduced pressure. The purification of the obtained residue was done by column chromatography on silica gel using ethyl acetate as eluents (100% ethyl acetate) to give the desired compound **(12)** as a red-orange solid [17].

Yield: 12.5 mg (27%). R_f: 0.45 (ethyl acetate 100%, visualized with $KMnO_4$ and UV illumination at 366 nm).

^{1}H NMR ($CDCl_3$, 500 MHz, ppm): δ 8.50 (d, J = 8.6 Hz, 1H, H_{ar}), 6.49 (br.s, 1H, CH_2N*H*), 6.29 (d, J =7.6 Hz, 1 H, CH_2O*H*), 6.17 (d, J = 8.7 Hz, 1H, H_{ar}), 5.77 (m, 1H, =C*H*CH_2), 5.54 (ddt, J = 1.4, 6.3, 15.4 Hz, 1H, =C*H*CHOH), 4.33 (m, 1H, C*H*OH), 3.95-3.99 (m, 1H, C*H*HOH), 3.92 (dq, J = 3.6, 7.4 Hz,2H, CH*H*OH), 3.69-3.74 (m, 1H, CHNH), 3.49 (q, J= 6.5, 2H, CH2NH), 2.72 (br s, 1H, OH), 2.24 (t, J= 7.6, 2H, CH_2CO), 2.06 (q, J= 7.1, 2H, =CHC*HH*), 1.81 (p, J= 7.3, 2H, CH_2),1.64 (p, J=7.6, 2H, CH_2), 1.46 (p, J= 6.9, 2H, CH_2), 1.25-1.39 (m, 34H, CH_2), 0.87 (t, J = 6.9 Hz, 3H, CH_3).

^{13}C-NMR (CD_3OD, 126 MHz, ppm): δ = 177.51 (CO), 143.85 (C_{ar}), 137.21 (C_{ar}), 134.08 (=CH), 134.02 (Car), 129.28 (=CH), 94.95 (Car), 73.94(CHOH), 62.09(CH_2OH), 54.91 (CHNH), 49.39 (CH_2NH), 36.99 (*C*H_2CO), 36.94 (*C*H2CH=), 32.49(CH_2), 32.21(CH_2), 29.98(CH_2), 29.96(CH_2), 29.94(CH_2), 29.81(CH_2), 29.67(CH_2), 29.64(CH_2), 29.61(4×CH_2), 29.59(3×CH_2), 29.53(CH_2), 29.43(CH_2), 29.38 (CH_2), 29.29 (CH_2), 27.22(CH_2), 26.07(CH_2), 22.97 (CH_2), 14.36 (CH_3).

ESI-MS *m/z* calcd for $C_{36}H_{63}N_5O_6$ $[M+H]^+$: m/z calc.: 660.4, found: 660.4

$C_{36}H_{62}N_5O_6Na$ $(M+Na)^+$: m/z calc.: 682.3 , found: 682.4

9.3 Synthesis of Ceramide-coumarine

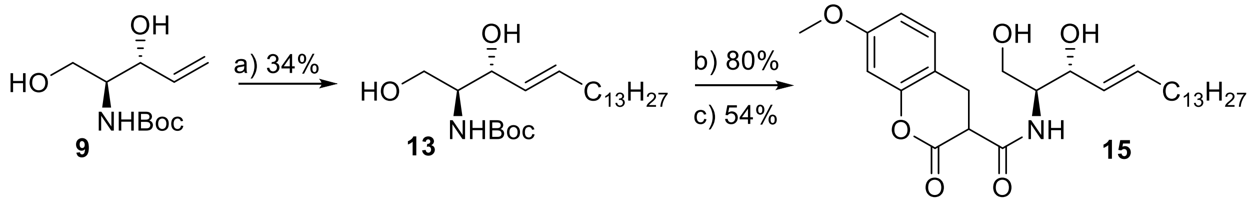

Scheme 3: Ceramide-CUM (15). Reagents and conditions: a) 1-pentadecene, Grubbs 2nd generation catalyst, reflux, 16h; b) TFA, DCM, 0°C-rt, 3h; c) MCSE, DIPEA, DCM, rt, 24h.

Synthesis of tert-butyl ((2S,3R,E)-1,3-dihydroxyoctadec-4-en-2-yl)carbamate (13)

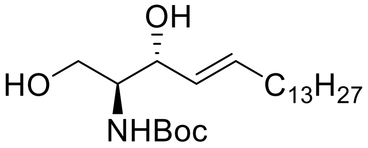

To a stirred solution of compound (**9**) (300mg, 1.38 mmol, 1eq) in dry dichloromethane (7 mL, 0.2 mol) under an argon atmosphere was added 1-pentadecene (1.5 mL, 5.5 mmol, 4 eq). After the reaction mixture was heated under reflux for 1 h at 40ºC, Grubbs 2nd generation catalyst (60 mg, 7 mol%) was added, and the heating was continued overnight at the same temperature. Afterwards, the solvent was removed under reduced pressure, and the crude product purified by column chromatography on silica gel using ethyl acetate and cyclohexane as eluents (45-50% ethyl acetate in cyclohexane) to give the product (**13**) as a brown oil which solidified upon standing for a long time.

Yield: 190 mg (34%). R*f*: 0.45 (ethyl acetate:cyclohexane 7:3, visualized with $KMnO_4$)

^{1}H NMR ($CDCl_3$, 500 MHz, ppm): δ 5.78 (dtd, *J* = 15.0, 6.8, 1.2 Hz, 1H, =C*H*CH2), 5.52 (ddt, *J* = 15.4, 6.5, 1.4 Hz, 1H, =C*H*CH), 5.30 (br, s, 1H, NH), 4.28-4.37 (m, 1H, C*H*OH), 3.98 – 3.93 (m, 1H, C*H*NH), 3.86-3.92 (m, 1H, C*H*HOH), 3.70 (ddd, *J* = 11.1, 6.9, 3.7 Hz, 1H, C*H*HOH), 3.60 (s, 1H, OH), 2.60 (m, 1H, OH), 2.05 (q, *J* = 7.2 Hz, 2H, CH_2CH=), 1.45 (s, 9H, (($CH_3)_3$CO), 1.24-1.38 (m, 22H, 11× CH_2), 0.87 (t, J=7.1, 3H, CH_3).

^{13}C-NMR (CD_3OD, 126 MHz, ppm): δ 156.78 (COO), 134.36 (=*CH*CH), 129.03 (=*CH*CH_2), 75.04 (CHOH, C_{ar}), 62.82 (CH_2OH), 62.46 (CHNH), 32.43 (CH_2), 32.06 (CH_2), 29.83 (CH_2), 29.82 (CH_2), 29.79 (CH_2), 29.75 (CH_2), 29.63 (CH_2), 29.50 (CH_2), 29.34 (CH_2), 29.24(CH_2), 28.51 (CH_2), 28.45(($CH_3)_3$CO), 22.83 (CH_2), 14.24(CH_3).

ESI-MS *m/z* calcd for $C_{23}H_{46}NO_4$ $[M+H]^+$: m/z calc.: 400.3, found: 400.4

$C_{23}H_{45}NO_4Na$ (M+Na)$^+$: m/z calc.: 422.3 , found: 422.4

(2*S*,3*R*,*E*)-2-aminooctadec-4-ene-1,3-diol (14)

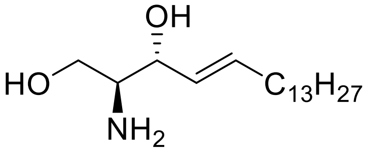

To a stirred solution of compound **(13)** (80 mg, 0.2 mmol) in dichloromethane (1.5mL, 0.14 M) was added 150 µL of trifluoroacetic acid (TFA) dropwise at room temperature. After the reaction mixture was stirred for 3 h, a Complete deprotection of the starting material checked by TLC analysis which visualized by ninhydrin staining. Then, the solvent was evaporated under reduced pressure and was kept under high vacuum for 2 days to get rid of the excess TFA. The obtained product **14** was used in the next step directly without any further purification.

Yield: 48 mg (80%). R*f*: 0.13 (100% ethyl acetate, visualized with ninhydrin).

***N*-((2*S*,3*R*,*E*)-1,3-dihydroxyhept-4-en-2-yl)-2-(7-methoxy-2-oxo-2H-chromen-3-yl) acetamide (15)**

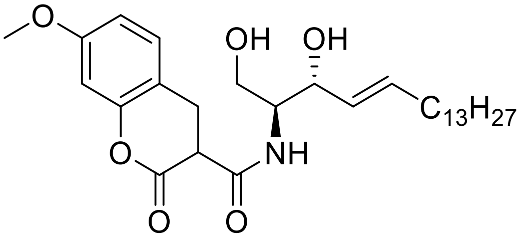

To a stirred solution of compound (**14**) (50 mg, 0.17 mmol, 1eq) in dry dichloromethane (1.7 mL, 0.1 M) under an argon atmosphere at 0ºC was added N,N-diisopropylethylamine (DIPEA) (233µL, 1.34 mmol, 8eq). *N*-Succinimidyl 7-methoxycoumarin-3-carboxylate (MCSU) (69 mg, 0.22 mmol, 1.3 eq) was dissolved in dry dichloromethane (2 mL, 0.1 M) and added dropwise on the reaction mixture at the same temperature. The resulting reaction mixture was stirred for 24 h at room temperature (TLC and UPLC analysis indicated the complete conversion to the product, visualized with ninhydrin). 2 mL methanol was added to the reaction mixture to quench and the reaction continued to stir for additional 1h. Dichloromethane (80mL) was added to the reaction mixture which subsequently washed twice with 40 mL of 0.1 M HCl. The organic layers were separated, collected, dried over anhydrous Na_2SO_4, and the solvent was evaporated under reduced pressure. The crude product purified by column chromatography on silica gel using methanol and

dichloromethane as eluents (2.5-5% Methanol in dichloromethane) to give the product (**15**) as pale yellow solid.

Yield: 45 mg (54%). R_f: 0.42 (5:95 methanol:dichloromethane, visualized with ninhydrin).
^{1}H NMR ($CDCl_3$, 500 MHz, ppm) δ 9.31 (d, *J* = 6.9 Hz, 1H, NH), 8.81 (s, 1H, CH_{ar}), 7.57 (d, *J* = 8.6 Hz, 1H, CH_{ar}), 6.93 (dd, *J* = 8.7, 2.4 Hz, 1H, CH_{ar}), 6.86 (d, *J* = 2.3 Hz, 1H, CH_{ar}), 5.83 (dt, *J* = 14.0, 6.7 Hz, 1H, =C*H*CH_2), 5.57 (dd, *J* = 15.4, 6.5 Hz, 1H, =C*H*CHOH), 4.43 (t, *J* = 5.4 Hz, 1H, C*H*OH), 4.12-4.15 (m, 1H, C*H*NH), 3.99 (tt, *J* = 6.0, 3.6 Hz, 1H, CH*H*OH), 3.91 (s, 3H, CH_3O), 3.85 (d, *J* = 10.0 Hz, 1H, C*H*HOH), 2.32 (br. S, 1H, OH), 2.05 (q, *J* = 7.3, 2H, CH_2 CH=), 1.27-1.38 (m, 4H, 2×CH_2), 1.22-1.27 (m, 12H, 6×CH_2), 1.20-1.22 (m, 6H, 3×CH_2), 0.87 (t, *J* = 6.9 Hz, 3H, CH_3).
^{13}C-NMR ($CDCl_3$, 126 MHz, ppm): δ 164.88 (CO), 161.49 (COO, C_{ar}), 156.66 (C_{ar}), 148.38 (CH), 134.66 (CH_{ar}), 130.88 (=CH), 128.48(CH_{ar}, =CH), 114.02 (CH_{ar}), 112.20 (C_{ar}), 100.18 (CH_{ar}), 74.00 (CHOH), 62.66 (CH_2OH), 55.93 (CHNH, CH_3O), 32.19 (CH_2), 31.81 (CH_2), 29.58 (CH_2), 29.56 (CH_2), 29.54 (CH_2), 29.49 (2×CH_2), 29.39 (CH_2), 29.25(CH_2), 29.08(CH_2), 28.98(CH_2), 22.58 (CH_2),14.01 (CH_3).
ESI-MS *m/z* calcd for $C_{29}H_{44}NO_6$ $[M+H]^+$: m/z calc.: 502.3, found: 502.3
$C_{29}H_{43}NO_6Na$ $[M+Na]^+$: m/z calc.: 524.3 , found: 524.3

9.4 Synthesis of Ceramide-C6-NileRed

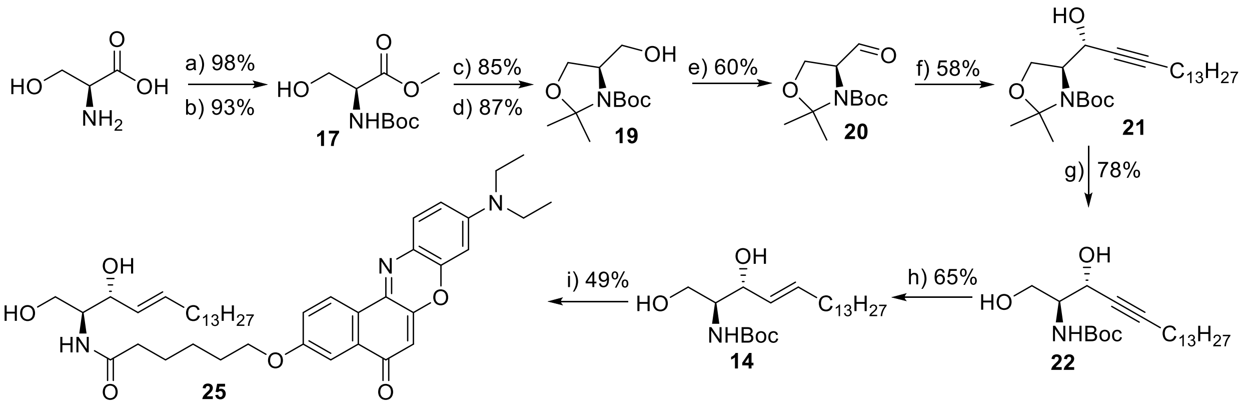

Scheme 4: Ceramide-C$_6$-NR (25). Reagents and conditions: a) $SOCl_2$, MeOH, -10°C-rt, 7h; b) Boc_2O, Et_3N, DCM, 0°C-rt, 18h; c) DMP, BF_3□Et_2O (cat.), acetone, rt, 4h; d) $LiAlH_4$, THF, -10°C-rt, 1h, ; e) $(COCl)_2$, DMSO, DIPEA, DCM, -78°C; f) (i) *t*-BuLi, pentadecyne, HMPA, THF, -78 °C, 2h, (ii) **20**, HMPA, THF, -78 °C, 3h; g) *p*-TsOH, MeOH, 45°C, 2h; h) Red-Al, Et_2O, -20°C-rt, 18h; i) (i) AcCl, MeOH, 0°C-rt, 2h, (ii) **24**, EDCI, HOBt, DCM, 0°C-rt, 16h.

Synthesis of (S)-4-((R)-1-hydroxyhexadec-2-yn-1-yl)-2,2-dimethyloxazolidine-3-carboxylate (21)

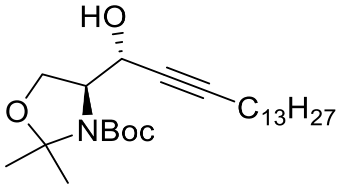

Compound **20** was obtained as previously prepared by Saied et al [3]. To a stirred solution of 1-pentadecyne (8.6 ml, 32.6 mmol, 2.2 eq) in dry THF (0.2 M) at -78 °C under argon atmosphere was added dropwise a solution of *tert*-butyllithium (16.6 ml, 28.2 mmol, 1.9 eq, 1.7M in pentane). After the resulting reaction mixture was stirred for 1.5h at the same conditions, a solution of hexamethylphosphoramide (5.2 ml, 29.7 mmol, 2.0 eq) was slowly added, followed by addition of a solution of Garner aldehyde **20** (3.4 g, 14.8 mmol, 1.0 eq, 1 M in dry THF). The resulting reaction mixture was allowed to stir at the same conditions for 2h (as indicated by TLC analysis, visualized with 1.3 % ninhydrin), before it was carefully quenched by slow addition of a saturated ammonium chloride solution (150 mL). The resulting mixture was extracted with ethyl acetate (3x100 mL), and the combined organic layers were washed with brine solution, dried over anhydrous Na_2SO_4, filtered and concentrated under reduced pressure. The obtained yellow oil was purified by flash column chromatography over silica gel using petroleum ether and ethyl acetate

as eluents (0-3% EA to collect pentadecyne, 8-12% EA to collect the product) to afford compound **21** as a pale yellow oil, which was identical to the compound described by Herold et al. [18].

Yield: 3.8 g (58%). R*f*: 0.61 (CH/EA 4:1, visualized with 1.3% ninhydrin).

^{1}H-NMR (500 MHz, $CDCl_3$, ppm): δ = 4.64 – 4.37 (m, 1H), 4.15 – 3.93 (m, 1H), 3.91 – 3.79 (m, 1H), 3.75 – 3.52 (m, 1H), 2.16 – 2.05 (m, 2H), 1.60 – 1.13 (m, 37H), 0.81 (t, *J* = 6.9 Hz, 3H).

^{13}C-NMR (126 MHz, $CDCl_3$, ppm): δ = 155.2, 99.5, 95.0, 86.8, 81.3, 65.2, 65.0, 64.3, 63.0, 32.0, 31.0, 29.8, 29.8, 29.6, 29.5, 29.2, 29.0, 29.0, 28.7, 28.7, 28.5, 28.4, 27.0, 25.9, 22.8, 18.9, 14.2.

ESI-MS *m/z* calcd for $C_{26}H_{47}NO_4Na^+$ $[M+Na]^+$ 460.4; observed 460.4.

Synthesis of Synthesis of *tert-butyl* ((2S,3R)-1,3-dihydroxyoctadec-4-yn-2-yl) carbamate (22)

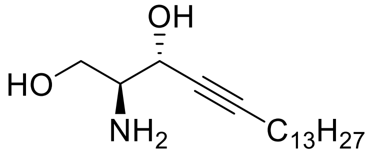

A solution of compound **21** (3.8 g, 8.6 mmol, 1.0 eq) in methanol (85 mL, 0.1M) was treated with a catalytic amount of *p*TsOH (10 mol%). After being stirred at 45°C for 2h (as indicated by TLC analysis), the solvent was removed under reduced pressure. The residue was partitioned between ethyl acetate and saturated $NaHCO_3$, and the layers were separated. The aqueous layer was extracted with ethyl acetate (3x 100mL), and the combined layers was washed with brine, dried over anhydrous Na_2SO_4, filtered and concentrated. The obtained crude mixture was purified by flash column chromatography over silica gel using cyclohexane and ethyl acetate as eluents (12-25% EA adduct eluted, and at 35% EA the product eluted) to afford compound **22** as a pale yellow-oil [18].

Yield: 2.7 g (78%). R*f*: 0.48 (CH/EA 3:2, visualized with 1.3% ninhydrin)

^{1}H-NMR (500 MHz, $CDCl_3$, ppm): δ = 5.33 (br, 1H), 4.64 – 4.57 (m, 1H), 4.13 – 4.05 (m, 1H), 3.81 – 3.70 (m, 2H), 2.21 (td, *J* = 7.2, 2.0 Hz, 2H), 1.54 – 1.46 (m, 2H), 1.45 (s, 9H), 1.39 – 1.31 (m, 2H), 1.25 (s, 18H), 0.87 (t, *J* = 7.0 Hz, 3H).

^{13}C-NMR (126 MHz, $CDCl_3$, ppm): δ = 156.3, 88.4, 78.0, 65.0, 63.1, 55.8, 32.1, 29.8, 29.8, 30.0, 29.5, 29.3, 29.0, 29.0, 28.5, 27.1, 22.8, 18.8, 14.3.

ESI-MS *m/z* calcd for $C_{23}H_{43}NO_4Na^+$ $[M+Na]^+$ 420.3; observed 420.3.

Synthesis of *tert-butyl* ((2S,3R,E)-1,3-dihydroxyoctadec-4-en-2-yl)carbamate (13)

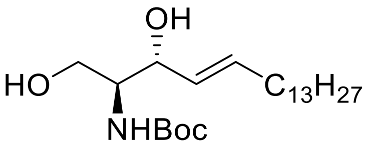

To a stirred solution of Red-Al (6.6 ml, 33.7 mmol, 5.0 eq) in dry Et_2O (3 M) at -20 °C under an argon atmosphere was added dropwise a solution of compound **22** (2.7 g, 6.7 mmol, 1.0 eq) in dry Et_2O (0.1 M). The reaction mixture was stirred for 30 min at the same conditions, then allowed to warm up gradually to ambient temperature and stirred for additional 16 hours (as indicated by TLC analysis, visualized with 1.3 % ninhydrin). The reaction was cooled again to -20°C and subsequently quenched by addition of MeOH (10 mL), followed by dropwise addition of 1M HCl (100 mL). The resulting mixture was extracted with EA (3x100 mL). The organic layers were combined, washed with brine solution, dried over anhydrous Na_2SO_4, filtered and concentrated. Purification of the obtained yellow oil by flash column chromatography over silica gel using cyclohexane and ethyl acetate as eluents (from 35%- 40% ethyl acetate in cyclohexane) afforded the desired product **13** as white solid [18].

Yield: 1,75 g (65%). R*f*: 0.46 (CH/EA 3:2, visualized with 1.3% ninhydrin).

^{1}H-NMR (400 MHz, $CDCl_3$, ppm): δ = 5.75 (dtd, *J* = 14.9, 6.7, 1.2 Hz, 1H), 5.50 (ddt, *J* = 15.4, 6.5, 1.4 Hz, 1H), 5.28 (br, 1H), 4.32 – 4.25 (m, 1H), 4.09 (q, *J* = 7.1 Hz, 1H), 3.90 (dd, *J* = 11.4, 3.8 Hz, 1H), 3.68 (dd, *J* = 11.3, 3.7 Hz, 1H), 3.57 (br, 1H), 2.06 – 1.99 (m, 2H), 1.43 (s, 9H), 1.38 – 1.29 (m, 2H), 1.31 – 1.16 (m, 20H), 0.85 (t, *J* = 7.1 Hz, 3H).

^{13}C-NMR (101 MHz, $CDCl_3$, ppm): δ = 156.4, 134.3, 129.1, 79.9, 75.0, 62.8, 60.6, 55.6, 32.4, 32.1, 29.8, 29.8, 29.8, 29.6, 29.5, 29.4, 29.3, 28.5, 22.8, 14.3.

ESI-MS *m/z* calcd for $C_{23}H_{46}NO_4^+$ $[M+H]^+$ 400.3; observed 400.3.

Synthesis of *tert*-butyl 6-((9-(diethylamino)-5-oxo-5H-benzo[a]phenoxazin-2-yl)oxy)hexanoate (23)

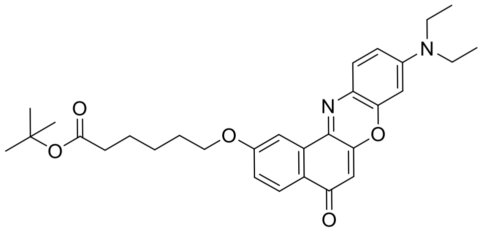

To a stirred solution of NR-OH (obtained in 2 steps from 1,6-dihydroxynaphthalene [19,20], 0.42 g, 1.25 mmol) and *tert*-butyl-6-bromo-hexanoate (0.375 g, 1.5 mmol) in dry DMF (6 mL) was added Cs_2CO_3 (1.22 g, 3.75 mmol). The resulting reaction mixture was allowed to heat at 80°C for 12h (as indicated by TLC analysis), before it was carefully quenched with 1M HCl solution (100 mL). The resulting mixture was diluted with ethyl acetate and the layers were separated. The aqueous layer was extracted with ethyl acetate (2x100 mL) and the combined extracts was washed with brine, dried over anhydrous Na_2SO_4, filtered and concentrated under reduced pressure. The resultant crude product was purified by flash column chromatography over silica gel using ethyl acetate and cyclohexane as eluents (from 35%- 40% ethyl acetate in cyclohexane) to afford the desired product **23** as deep red solid.

Yield: 360 mg (64%). R_f: 0.46 (CH/EA 4:1, visualized with UV lamp)

^{1}H-NMR (500 MHz, $CDCl_3$, ppm): δ 8.20 (d, *J* = 8.7 Hz, 1H), 8.02 (d, *J* = 2.6 Hz, 1H), 7.58 (d, *J* = 9.1 Hz, 1H), 7.14 (dd, *J* = 8.7, 2.6 Hz, 1H), 6.63 (dd, *J* = 9.1, 2.7 Hz, 1H), 6.43 (d, *J* = 2.7 Hz, 1H), 6.28 (s, 1H), 4.16 (t, *J* = 6.4 Hz, 2H), 3.45 (q, *J* = 7.1 Hz, 4H), 2.28 (t, *J* = 7.3 Hz, 2H), 1.93 – 1.83 (m, 2H), 1.70 (dt, *J* = 14.4, 7.2 Hz, 2H), 1.61 – 1.51 (m, 2H), 1.45 (s, 9H), 1.25 (t, *J* = 7.1 Hz, 6H).

^{13}C-NMR (120 MHz, $CDCl_3$, ppm): δ 183.42, 173.19, 161.90, 152.16, 150.81, 146.96, 140.17, 134.18, 131.18, 127.83, 125.68, 124.80, 118.42, 109.58, 106.65, 105.41, 96.40, 80.24, 68.19, 45.19, 35.61, 29.08, 28.26, 25.74, 24.98, 12.75.

ESI-MS *m/z* calcd for $C_{30}H_{37}N_2O_5$ $[M+H]^+$ 505.27; observed 505.3.

Synthesis of 6-((9-(diethylamino)-5-oxo-5H-benzo[a]phenoxazin-2-yl)oxy)hexanoic acid (24)

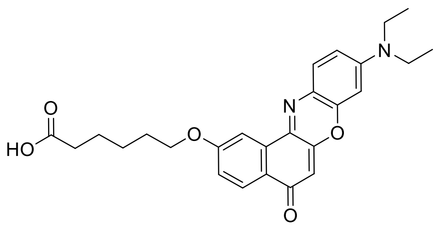

A solution of compound **23** (200 mg, 0.4 mmol) in CH_2Cl_2 (5 mL) at 0°C was treated dropwisely with a solution of triflouroacetic acid in dichloromethane (TFA-DCM) (3:1; 5 mL). After being stirred at the same conditions for 30min, and for additional 1h at ambient temperature (as monitored by TLC analysis for complete deprotection of *tert*-butyl ester), the reaction mixture was diluted with CH_2Cl_2 (80 mL) and subsequently quenched with a saturated $NaHCO_3$ solution (70

mL). The layers were separated and the aqueous layer was extracted with CH_2Cl_2 (3x 50 mL). The combined organic layer were sequentially washed with water (100 mL), brine (100 mL), dried over anhydrous Na_2SO_4, filtered and concentrated under reduced pressure using a high vacuum line to afford compound **24** as violet solid. The compound was obtained in a pure form (as determined by NMR, ESI-MS analysis), and was used directly in the next step without any further purification. Yield: 166 mg (93%). R_f: 0.28 (DCM/MeOH 8:2, visualized with UV lamp)

^{1}H-NMR (500 MHz, DMSO, ppm): δ 8.01 (d, *J* = 8.7 Hz, 1H), 7.90 (d, *J* = 2.3 Hz, 1H), 7.60 (d, *J* = 9.1 Hz, 1H), 7.22 (dd, *J* = 8.7, 2.3 Hz, 1H), 6.79 (dd, *J* = 9.2, 2.3 Hz, 1H), 6.62 (d, *J* = 2.3 Hz, 1H), 6.16 (s, 1H), 4.14 (t, *J* = 6.3 Hz, 2H), 3.49 (dd, *J* = 14.0, 6.9 Hz, 4H), 2.26 (t, *J* = 7.1 Hz, 2H), 1.85 – 1.73 (m, 2H), 1.59 (dd, *J* = 14.5, 7.2 Hz, 2H), 1.53 – 1.44 (m, 2H), 1.16 (t, *J* = 6.9 Hz, 6H).

^{13}C-NMR (1201 MHz, DMSO, ppm): δ 181.39, 174.45, 161.27, 151.70, 150.82, 146.45, 139.15, 138.27, 133.55, 130.96, 127.22, 124.84, 123.93, 110.06, 106.24, 104.05, 95.98, 67.86, 44.45, 33.64, 28.37, 25.11, 24.28, 12.46, 12.46.

ESI-MS *m/z* calcd for $C_{26}H_{29}N_2O_5$ $[M+H]^+$ 449.21; observed 449.2.

Synthesis of 6-((9-(diethylamino)-5-oxo-5H-benzo[a]phenoxazin-3-yl)oxy)-*N*-((2*S*,3*R*,*E*)-1,3-dihydroxy-octadec-4-en-2-yl)hexanamide (25)

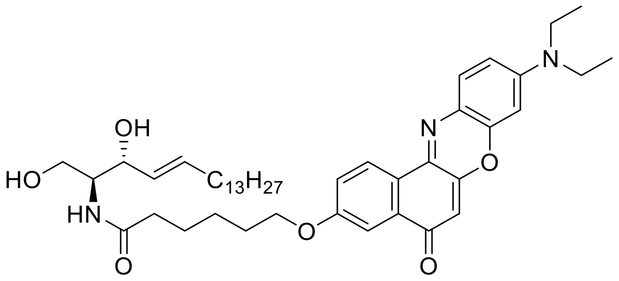

To a stirred solution mixture of sphingosine (**14**) (25 mg, 83.61 nmol, 1 eq) dissolved in dichloromethane (836 µL, 0.1 M) and DIPEA (36.4 µL, 209 nmol, 2.5 eq) at 0º C were added sequentially a solution of compound (**24**) in DCM (120 µL, 0.1 M), N-(3-Dimethylaminopropyl)-N′-ethylcarbodiimide hydrochloride EDC.HCl (13.95 mg, 91.97 nmol, 1.1 eq), and HOBt (14 mg, 91.97 nmol, 1.1 eq). The reaction mixture was warmed gradually to room temperature and continued to stir overnight with the exclusion of light (UPLC and TLC analysis indicated the completion of the reaction). After the evaporation of the solvent under reduced pressure, The crude product immediately purified by flash column chromatography on silica gel using cyclohexane,

ethyl acetate, methanol as eluents (CH:EA:MeOH= 1:2:0→0:98:2) to afford the product (**25**) as dark-violet solid.

Yield: 30 mg (49%). R_f: 0.62 (100% ethyl acetate, visualized with UV lamp).

^{1}H NMR ($CDCl_3$, 500 MHz, ppm) δ 8.18 (d, *J* = 8.7 Hz, 1H, CH_{ar}), 8.00 (d, *J* = 2.5 Hz, 1H, CH_{ar}), 7.60 (d, *J* = 9.0 Hz, 1H, CH_{ar}), 7.12 (dd, *J* = 8.7, 2.6 Hz, 1H, CH_{ar}), 6.67 (dd, *J* = 9.1, 2.7 Hz, 1H, CH_{ar}), 6.52 – 6.40 (m, 2H, CH_{ar}, OH), 6.31 (s, 1H, CH_{ar}), 5.83 – 5.73 (m, 1H, =C*H*CH_2), 5.54 (ddt, *J* = 15.4, 6.5, 1.6 Hz, 1H, =C*H*CHOH), 4.38 – 4.31 (m, 1H, C*H*OH), 4.10 – 4.18 (m, 2H, CH_2O), 3.98 (dd, *J* = 11.2, 3.7 Hz, 1H, C*H*HOH), 3.94 (dq, *J* = 7.4, 3.5 Hz, 1H, C*H*-NH), 3.73 (dd, 1H, CH*H*OH), 3.51 – 3.44 (m, 4H, 2× NCH_2Me), 2.32 (t, *J* = 7.4 Hz, 2H, CH_2CON), 2.07 – 1.99 (m, 2H, CH_2CH=), 1.89 (p, *J* = 6.5 Hz, 2H, C$H_2$$CH_2$O), 1.78 (p, *J* = 7.4 Hz, 2H, C$H_2$$CH_2$CO), 1.59 (tt, *J* = 6.3, 9.7 Hz, 2H,CH_2), 1.21-1.36 (m, 28H, 2×11 CH_2, 2×NCH_2CH_3), 0.88 (t, 3H, *J* = 7.0 Hz, CH_3).

^{13}C-NMR ($CDCl_3$, 126 MHz, ppm): δ =183.49 (CO), 173.65 (CONH), 161.92 (C_{ar}O), 152.27 (C_{ar}), 150.93 (C_{ar}), 147.03 (2×C_{ar}), 140.10 (=CH), 134.28 (CH_{ar}), 131.20(C_{ar}), 129.00 (=CH), 127.91(CH_{ar}), 125.68 (C_{ar}), 124.84(CH_{ar}), 118.28 (CH_{ar}), 109.72 (CH_{ar}), 106.91 (CH_{ar}), 105.40 (CH_{ar}), 96.45 (CH_{ar}), 74.92 (CHOH), 68.24(CH_2O), 62.62 (CH_2OH), 54.54 (CHNH), 45.23 (2 ×NCH_2), 36.79 (CH_2CO), 32.43 (CH_2), 32.07 (CH_2), 29.83 (CH_2), 29.80 (CH_2), 29.77 (CH_2), 29.64(CH_2), 29.50 (CH_2), 29.37 (CH_2), 29.26 (2×CH_2), 29.07 (CH_2), 29.02(CH_2), 25.88(CH_2), 25.58 (CH_2), 22.83 (*C*H_2 CH_3),14.26 (2×CH_3), 12.77 (CH_3).As reported [21].

ESI-MS *m/z* calcd for $C_{44}H_{64}N_3O_6$ $[M+H]^+$: 730.4, observed: 730.3

$C_{44}H_{63}N_3O_6Na$ $(M+Na)^+$: 752.4 , found: 752.3

10. References

[1] K. Hanada, K. Kumagai, S. Yasuda, Y. Miura, M. Kawano, M. Fukasawa, M. Nishijima, *Nature* **2003**, *426*, 803–809.

[2] N. Kudo, K. Kumagai, N. Tomishige, T. Yamaji, S. Wakatsuki, M. Nishijima, K. Hanada, R. Kato, *Proceedings of the National Academy of Sciences of the United States of America* **2008**, *105*, 488–493.

[3] E. M. Saied, S. Diederich, C. Arenz, *Chemistry – An Asian Journal* **2014**, *9*, 2092–2094.

[4] A. Gulbins, F. Schumacher, K. A. Becker, B. Wilker, M. Soddemann, F. Boldrin, C. P. Müller, M. J. Edwards, M. Goodman, C. C. Caldwell, et al., *Mol. Psychiatry* **2018**, *23*, 2324–2346.

[5] O. Trott, A. J. Olson, *Journal of Computational Chemistry* **2009**, *31*, NA-NA.

[6] J. Wang, P. Cieplak, P. A. Kollman, *Journal of Computational Chemistry* **2000**, *21*, 1049–1074.

[7] "Gaussian 09, Revision, A. 1 – ScienceOpen," can be found under https://www.scienceopen.com/document?vid=7625a2b3-85a4-4746-8a93-fb3335021944, **n.d.**

[8] K. Lindorff-Larsen, S. Piana, K. Palmo, P. Maragakis, J. L. Klepeis, R. O. Dror, D. E. Shaw, *Proteins: Structure, Function, and Bioinformatics* **2010**, *78*, NA-NA.

[9] J. Wang, R. M. Wolf, J. W. Caldwell, P. A. Kollman, D. A. Case, *Journal of Computational Chemistry* **2004**, *25*, 1157–1174.

[10] W. L. Jorgensen, J. Chandrasekhar, J. D. Madura, R. W. Impey, M. L. Klein, *The Journal of Chemical Physics* **1983**, *79*, 926–935.

[11] I. S. Joung, T. E. Cheatham, *Journal of Physical Chemistry B* **2008**, *112*, 9020–9041.

[12] D. Van Der Spoel, E. Lindahl, B. Hess, G. Groenhof, A. E. Mark, H. J. C. Berendsen, *Journal of Computational Chemistry* **2005**, *26*, 1701–1718.

[13] G. A. Tribello, M. Bonomi, D. Branduardi, C. Camilloni, G. Bussi, *Computer Physics Communications* **2014**, *185*, 604–613.

[14] L. Wang, R. A. Friesner, B. J. Berne, *Journal of Physical Chemistry B* **2011**, *115*, 9431–9438.

[15] A. Patriksson, D. Van Der Spoel, *Physical Chemistry Chemical Physics* **2008**, *10*, 2073–2077.

[16] B. R. Miller, T. D. McGee, J. M. Swails, N. Homeyer, H. Gohlke, A. E. Roitberg, *Journal of Chemical Theory and Computation* **2012**, *8*, 3314–3321.

[17] E. M. Saied, S. Banhart, S. E. Bürkle, D. Heuer, C. Arenz, *Future Medicinal Chemistry* **2015**, *7*, 1971–1980.

[18] P. Herold, *Helvetica Chimica Acta* **1988**, *71*, 354–362.

[19] M. Börgardts, K. Verlinden, M. Neidhardt, T. Wöhrle, A. Herbst, S. Laschat, C. Janiak, T. J. J. Müller, *RSC Adv.* **2016**, *6*, 6209–6222.

[20] M. S. J. Briggs, I. Bruce, J. N. Miller, C. J. Moody, A. C. Simmonds, E. Swann, *J. Chem. Soc., Perkin Trans. 1* **1997**, 1051–1058.

[21] T. Pinkert, D. Furkert, T. Korte, A. Herrmann, C. Arenz, *Angewandte Chemie International Edition* **2017**, *56*, 2790–2794.

11. NMR Spectra for Synthesized compounds

^{1}H-NMR spectrum for 21

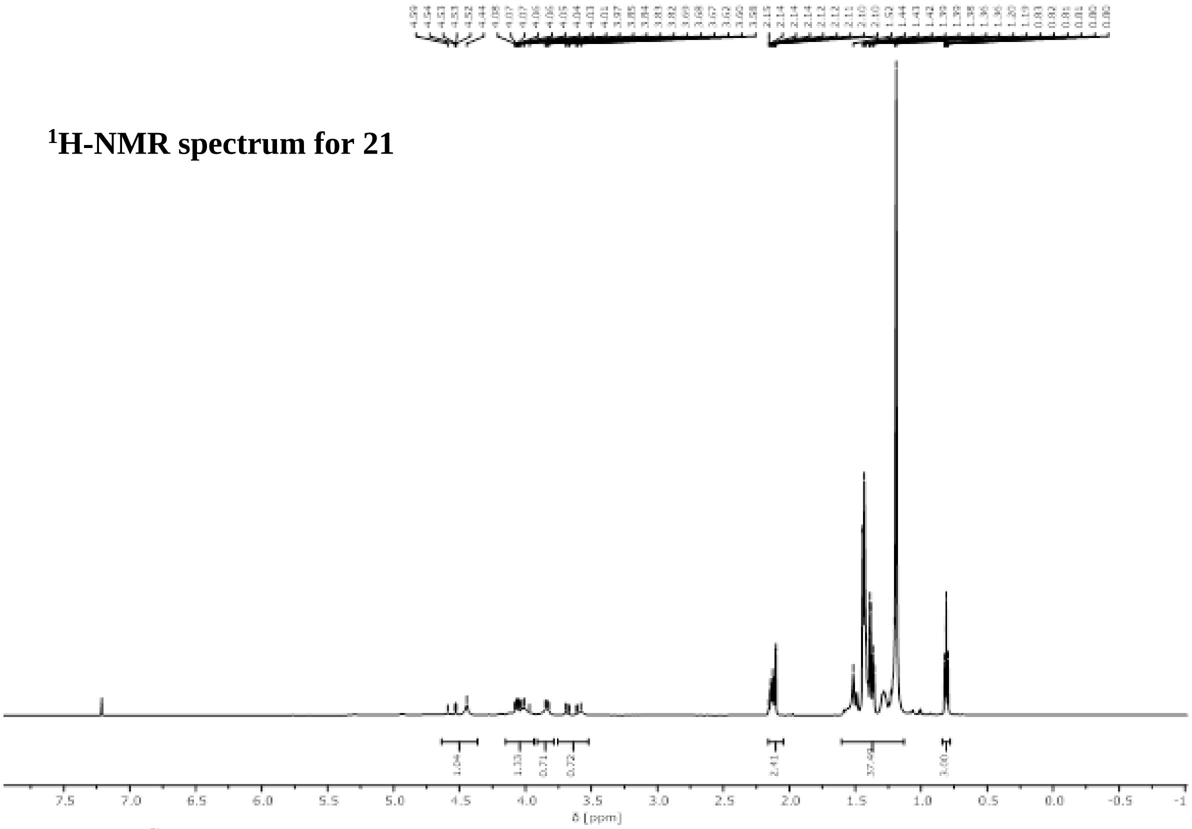

^{13}C-NMR spectrum for 21

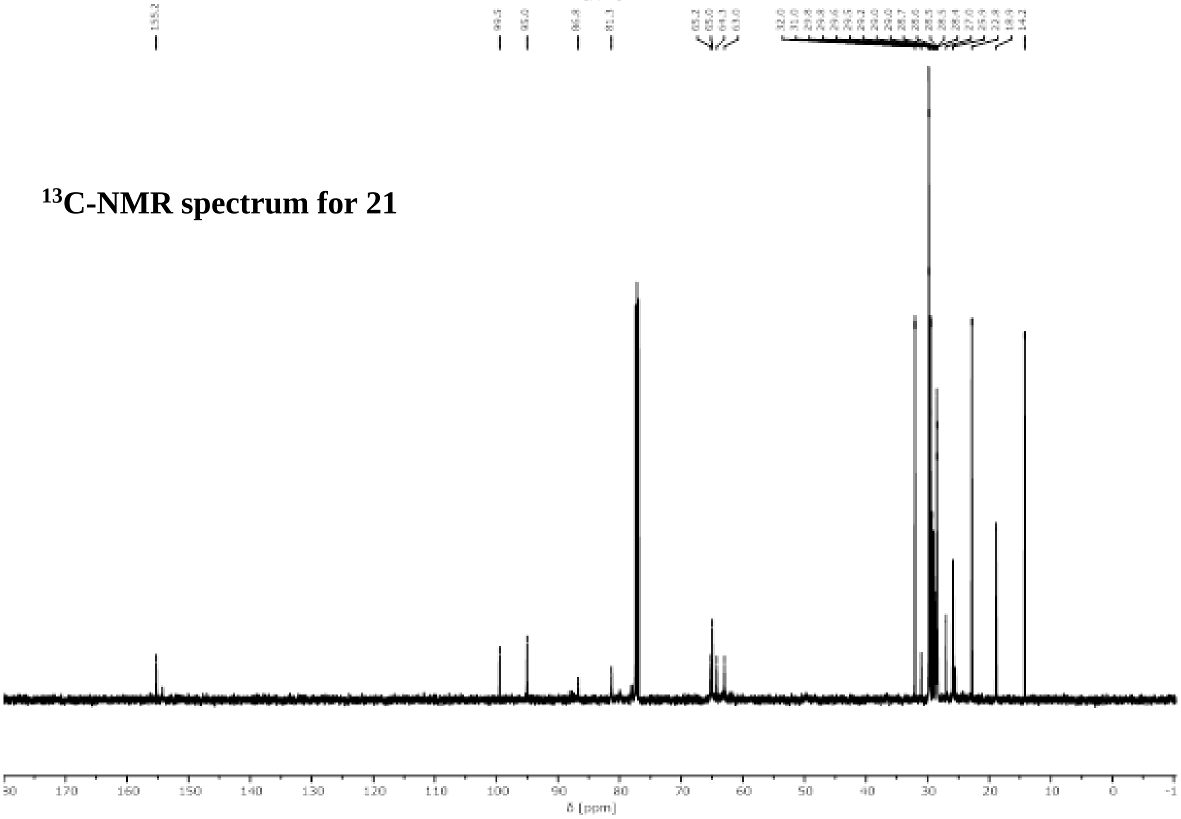

^{1}H-NMR spectrum for 22

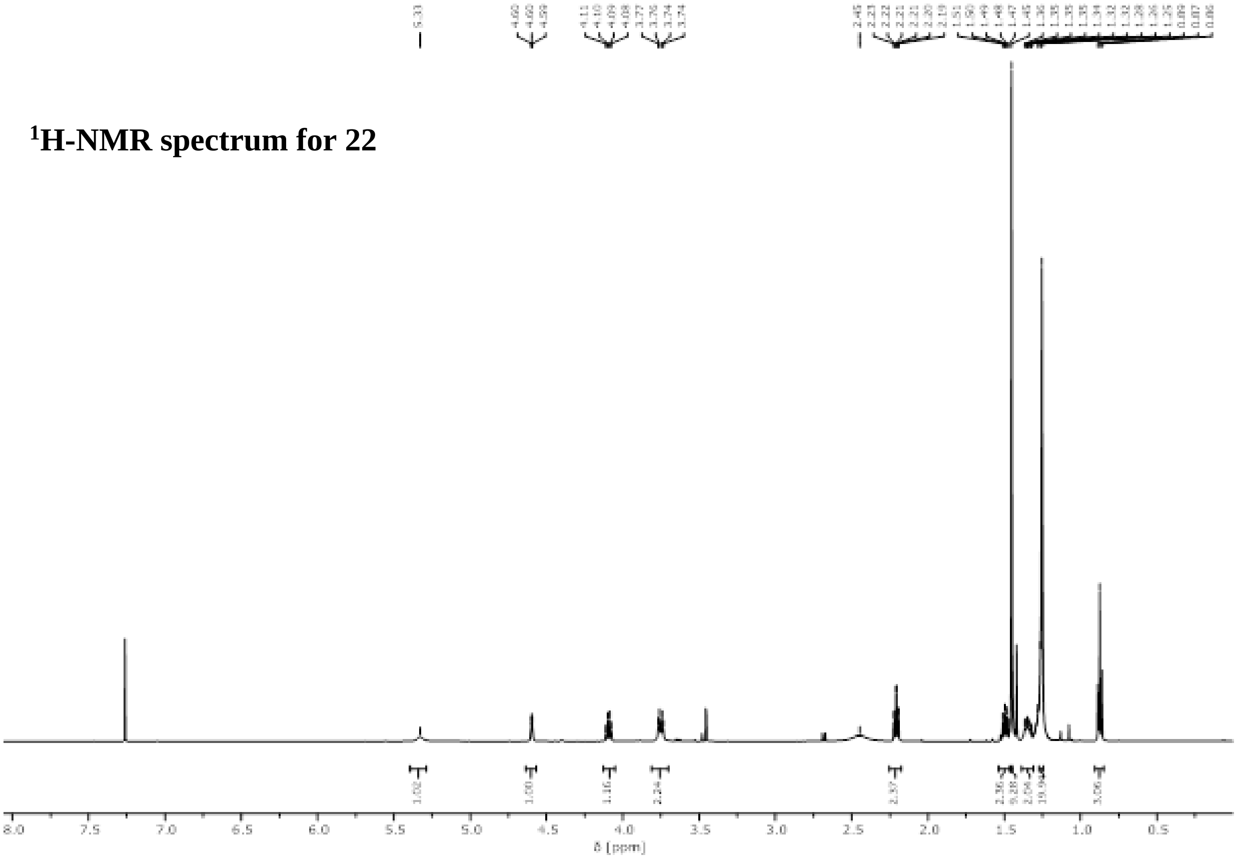

^{13}C-NMR spectrum for 22

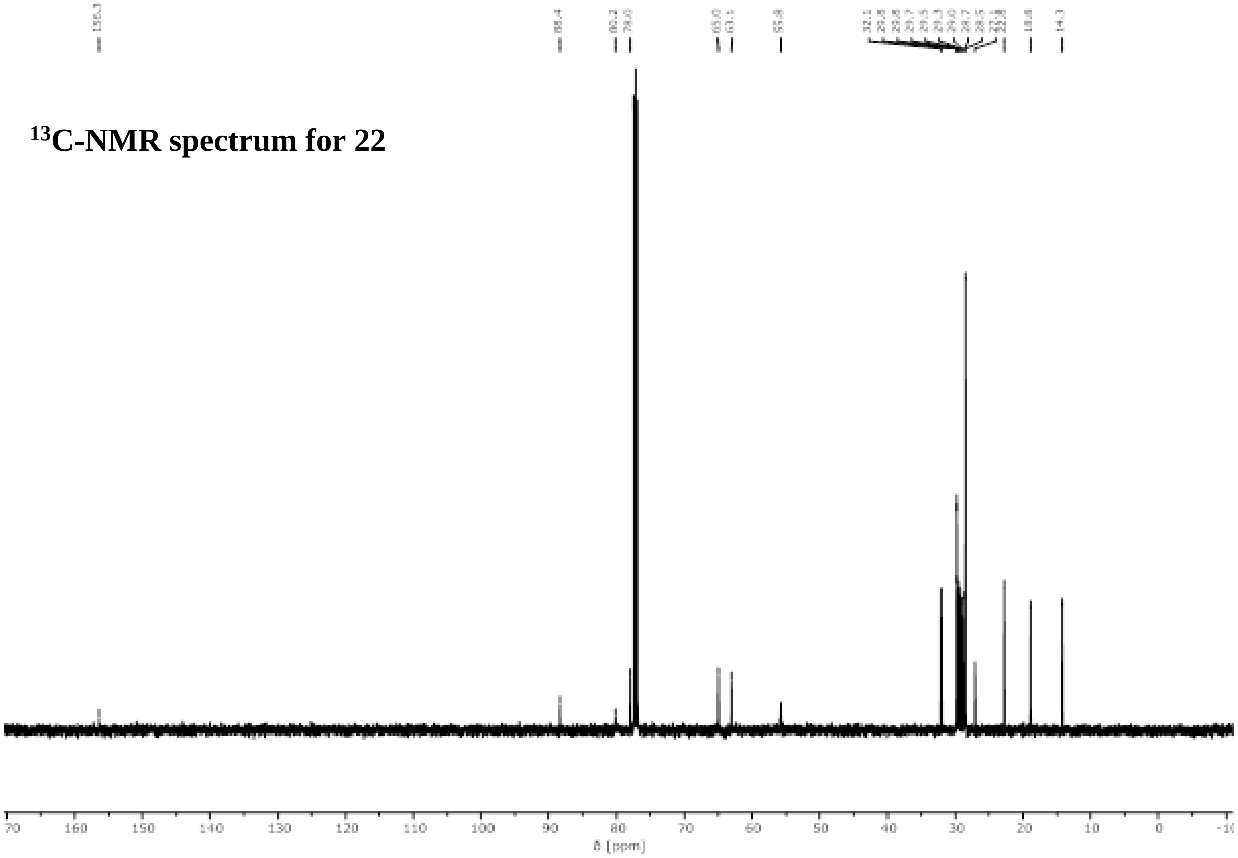

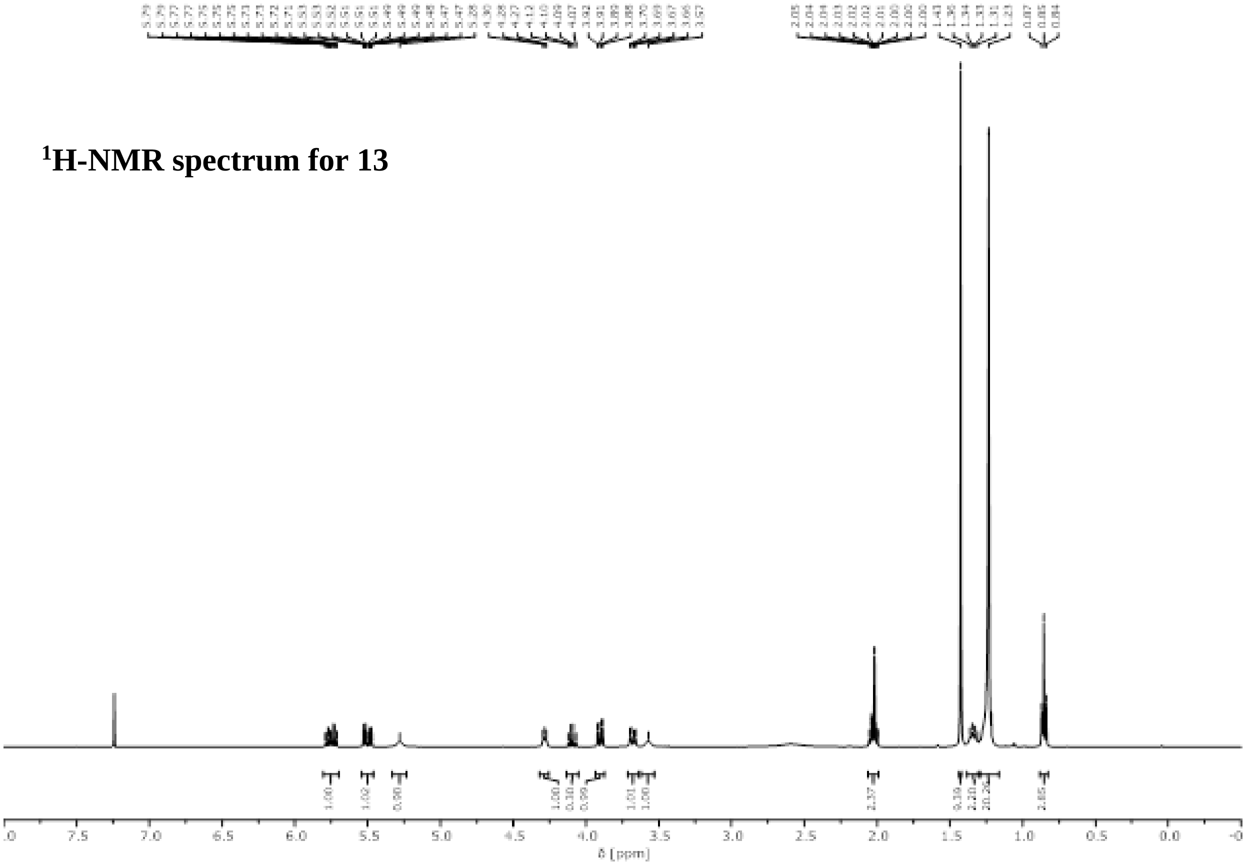

^{1}H-NMR spectrum for 13

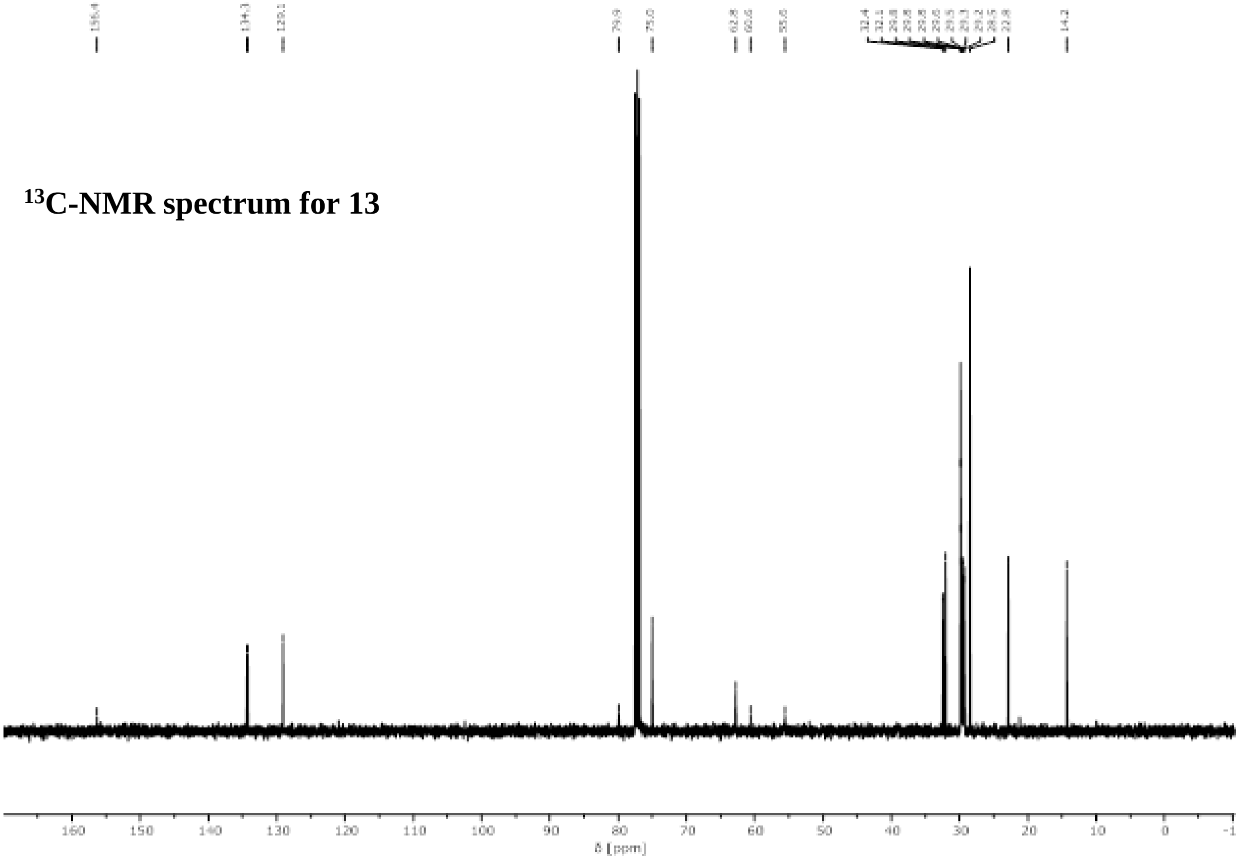

^{13}C-NMR spectrum for 13

^{1}H-NMR spectrum for 23

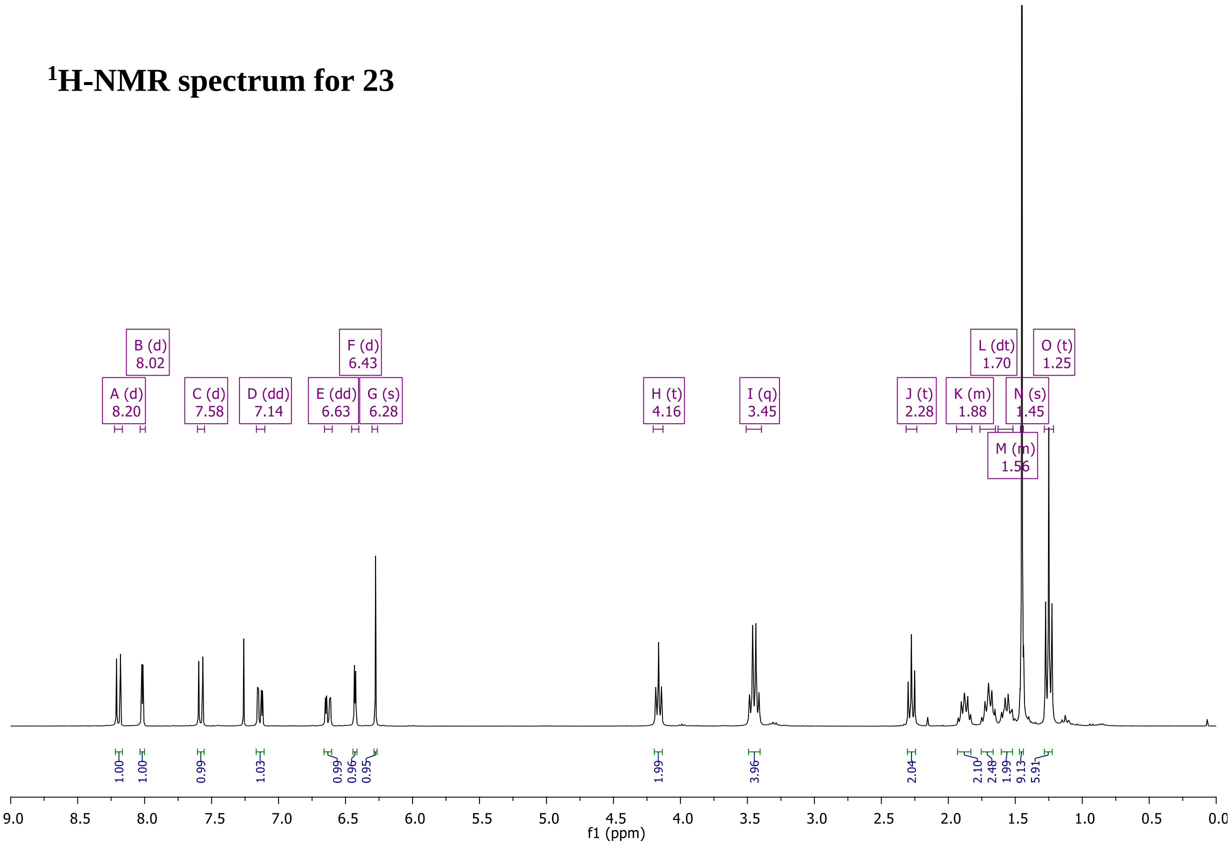

^{13}C-NMR spectrum for 23

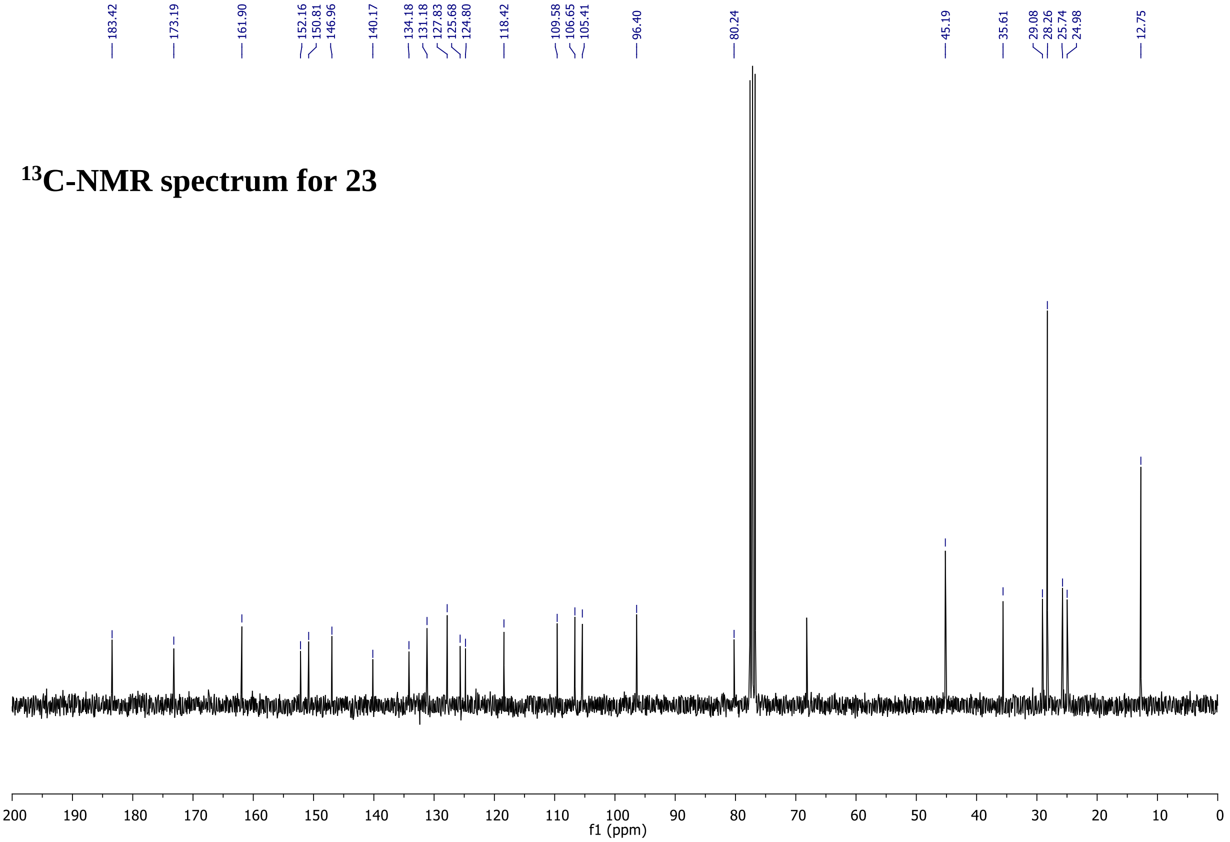

^{1}H-NMR spectrum for 24

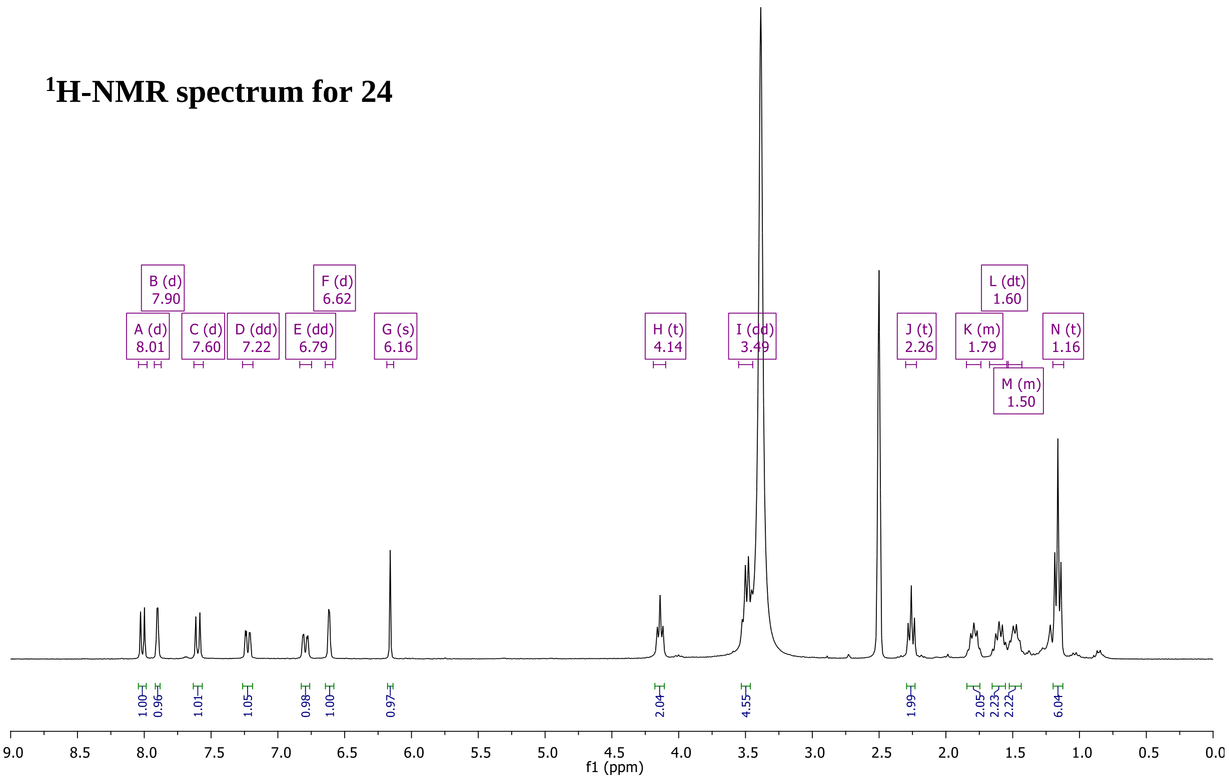

^{13}C-NMR spectrum for 24

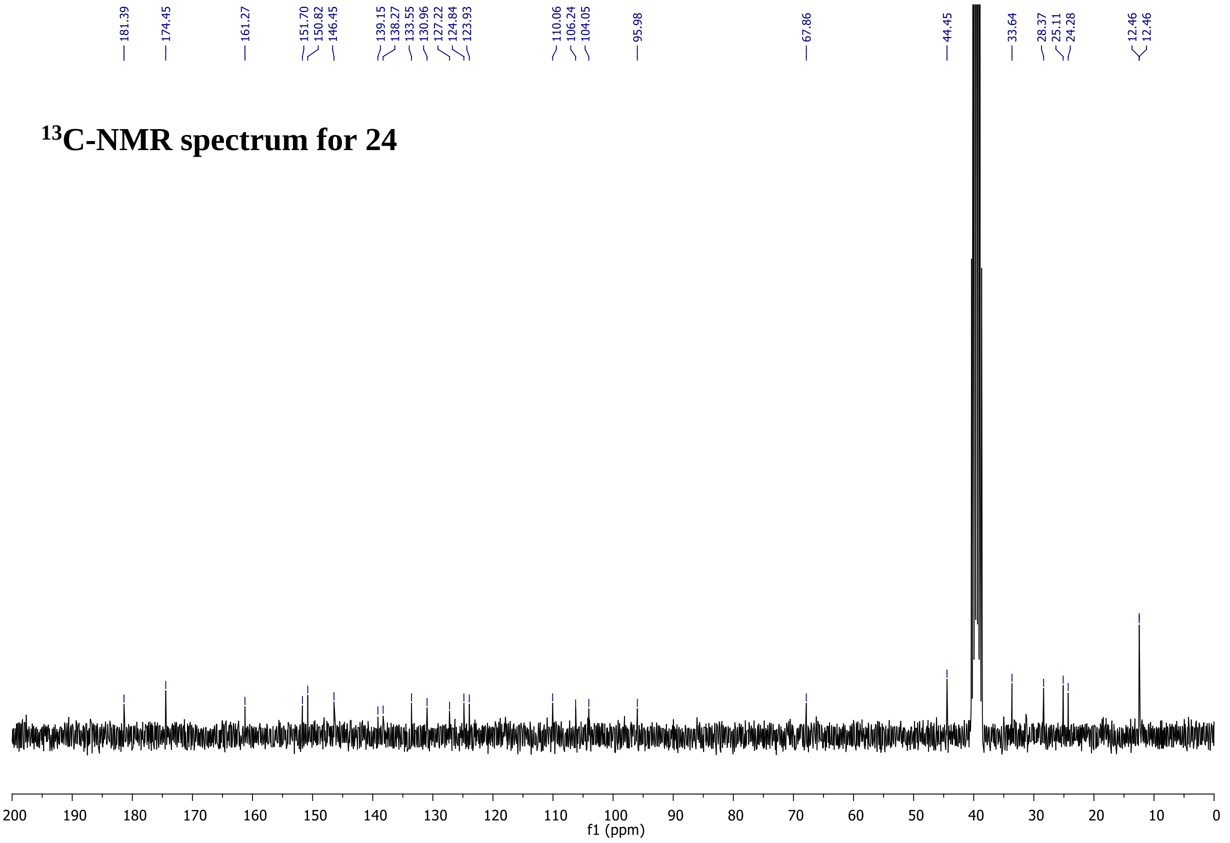

^{1}H-NMR spectrum for 25

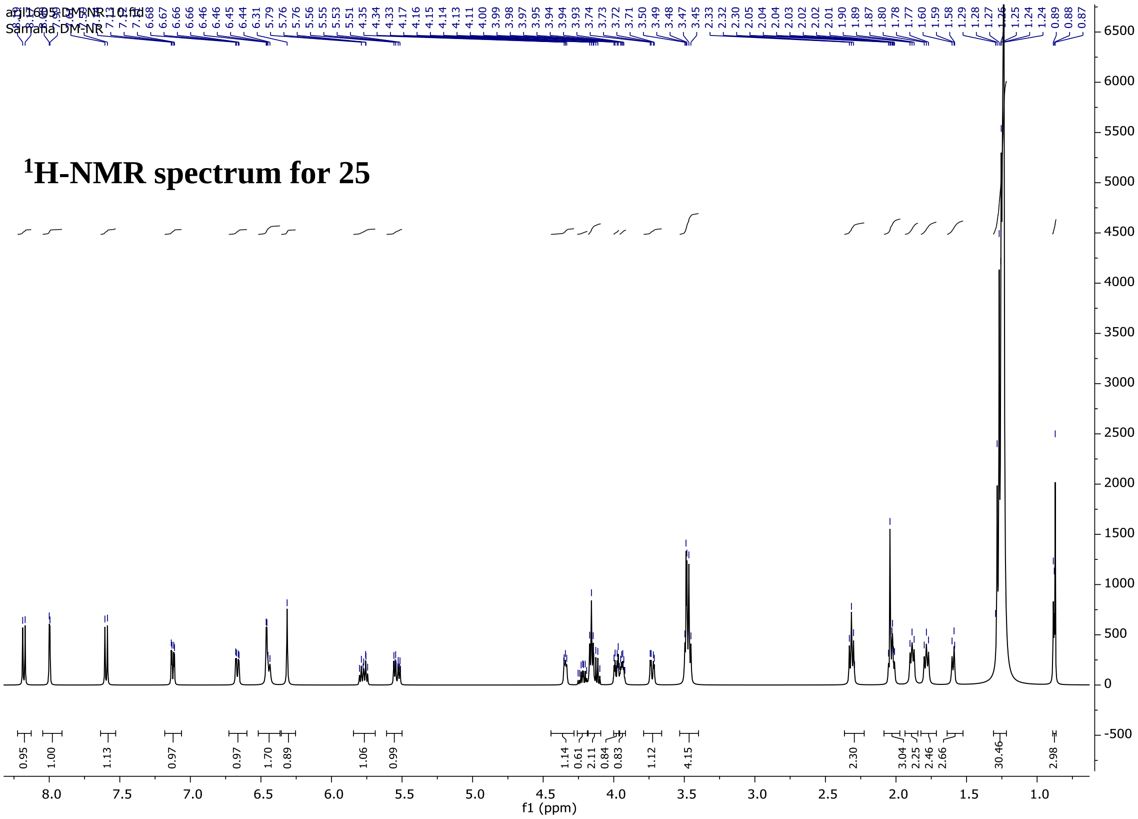

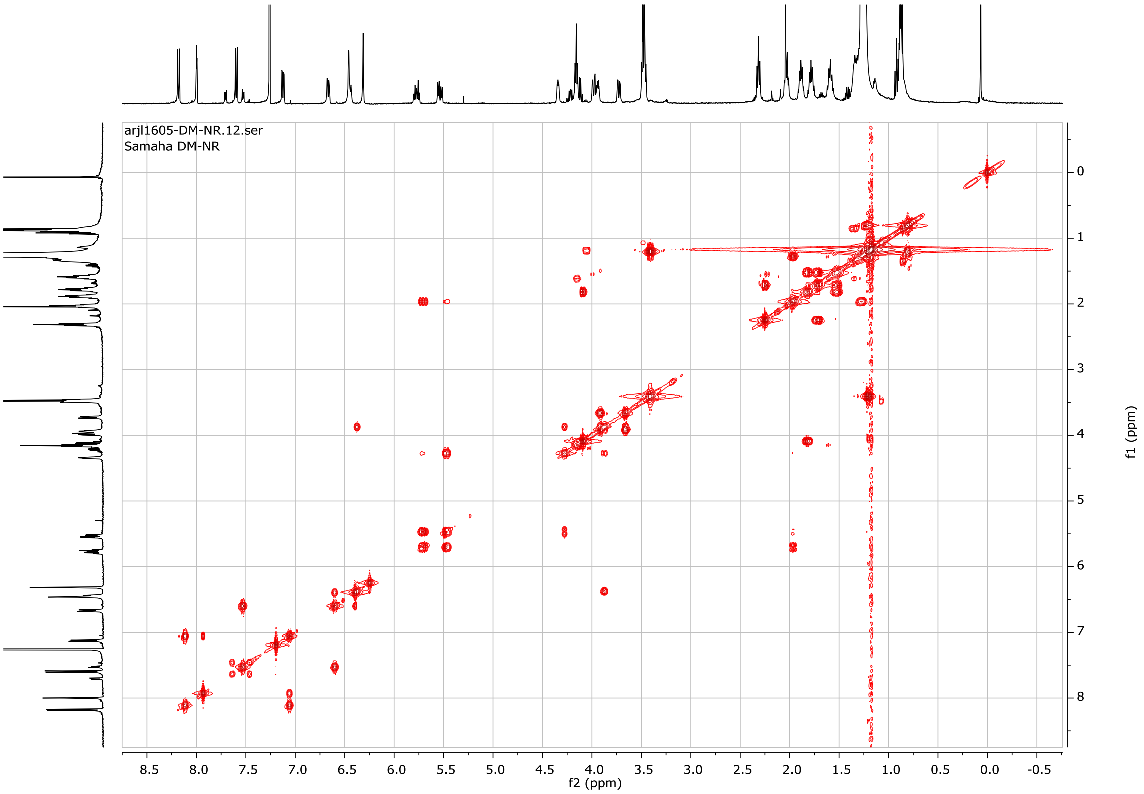

^{13}C-NMR spectrum for 25

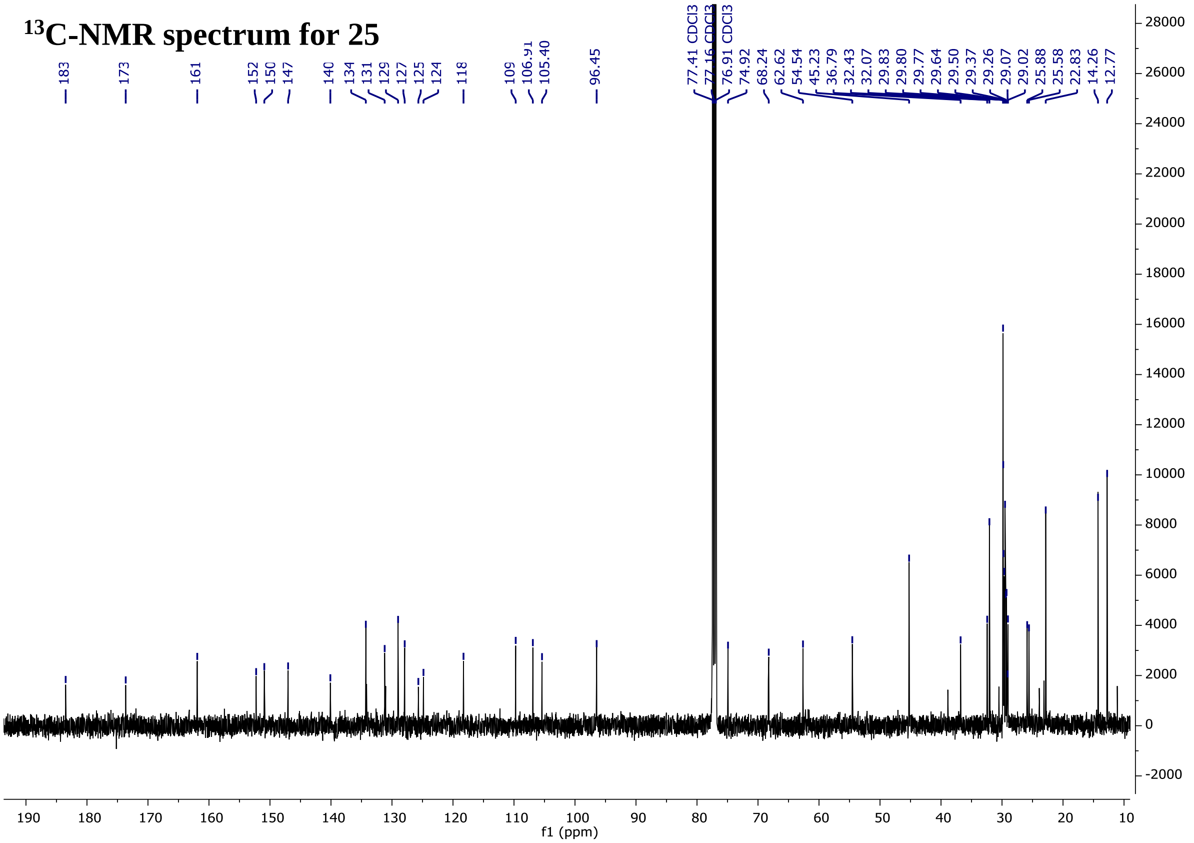

^{1}H-NMR spectrum for 5

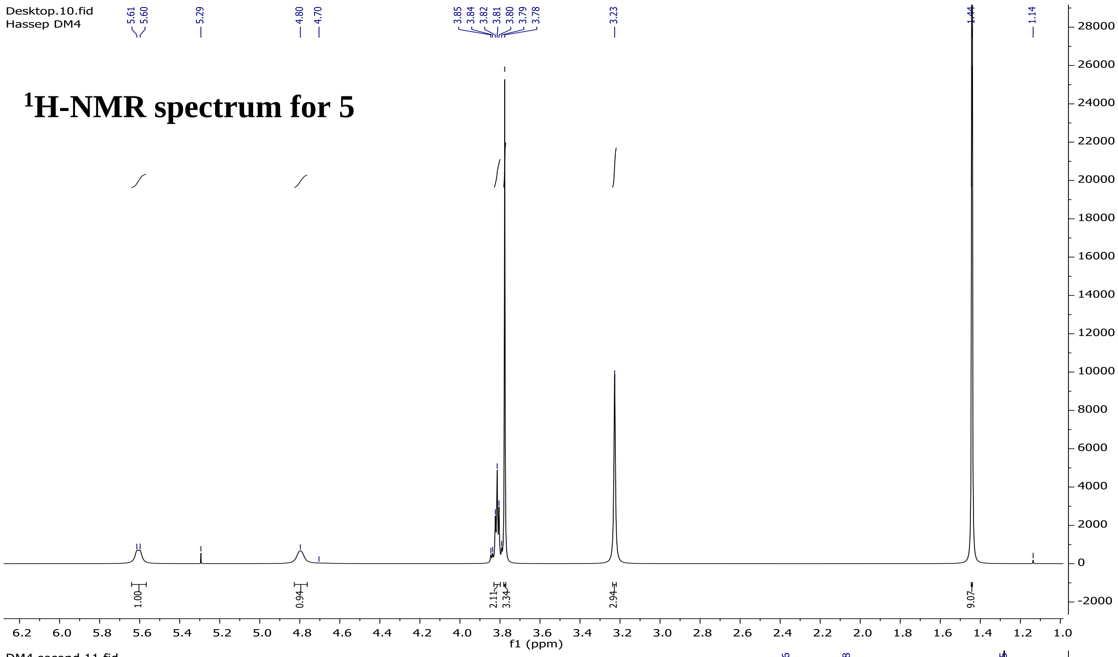

^{13}C-NMR spectrum for 5

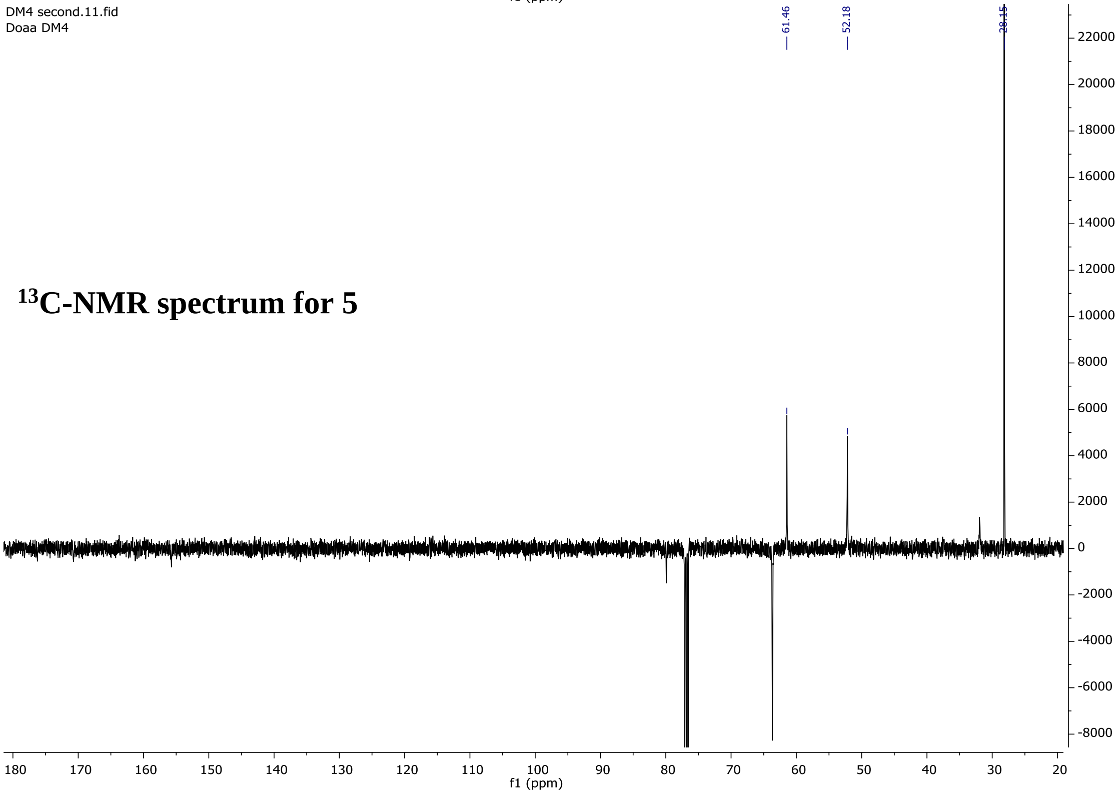

^{1}H-NMR spectrum for 6

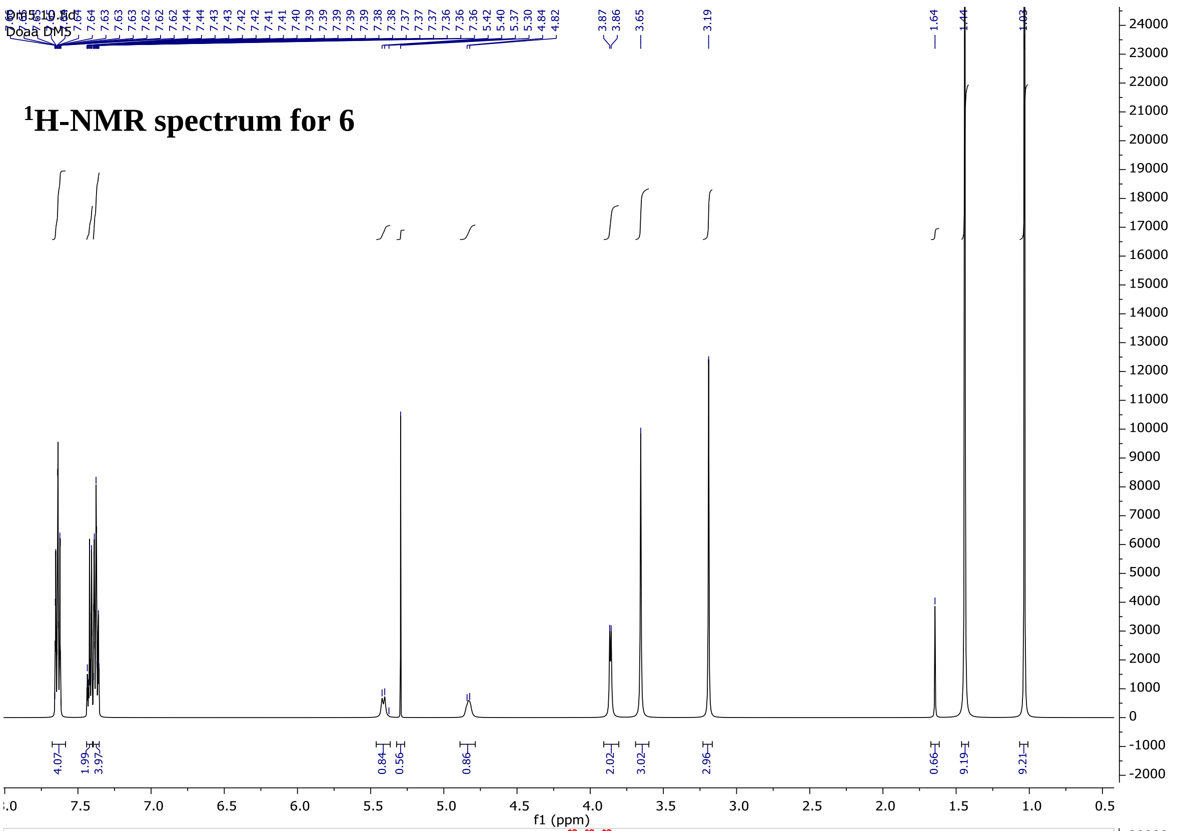

^{13}C-NMR spectrum for 6

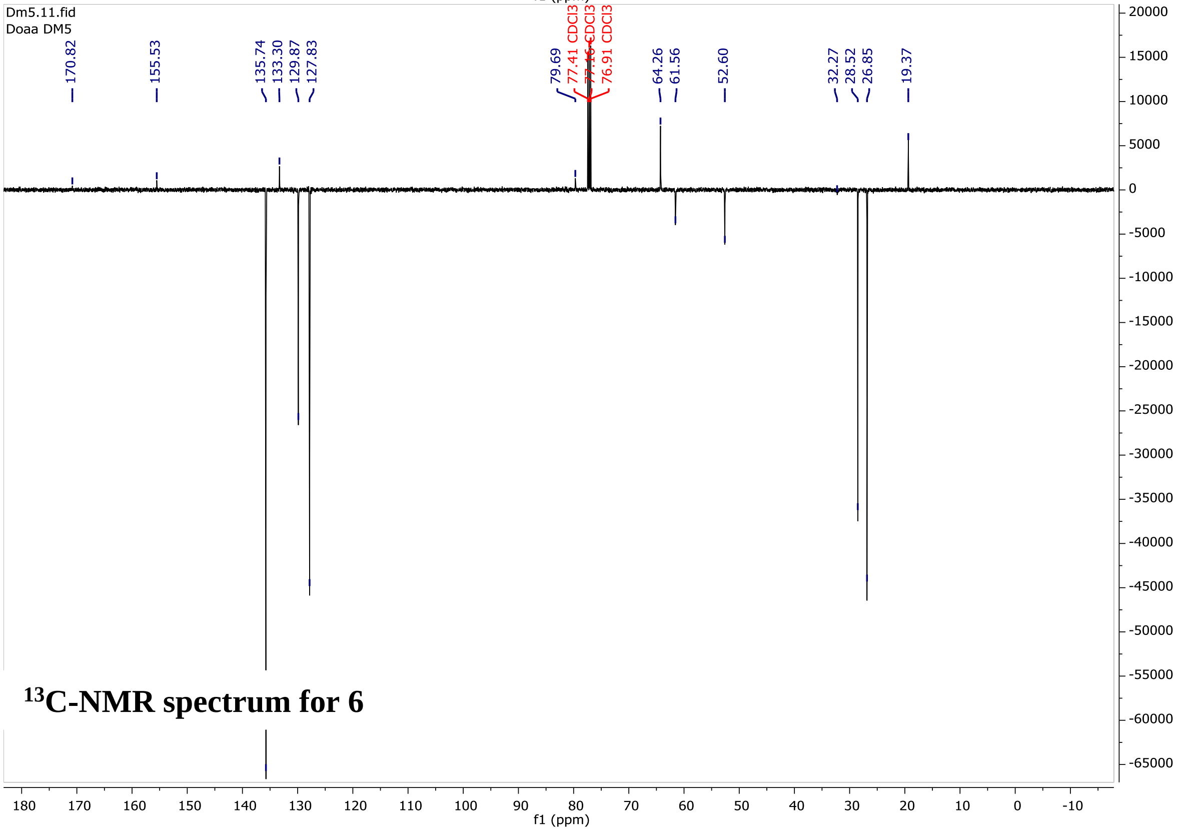

^{1}H-NMR spectrum for 7

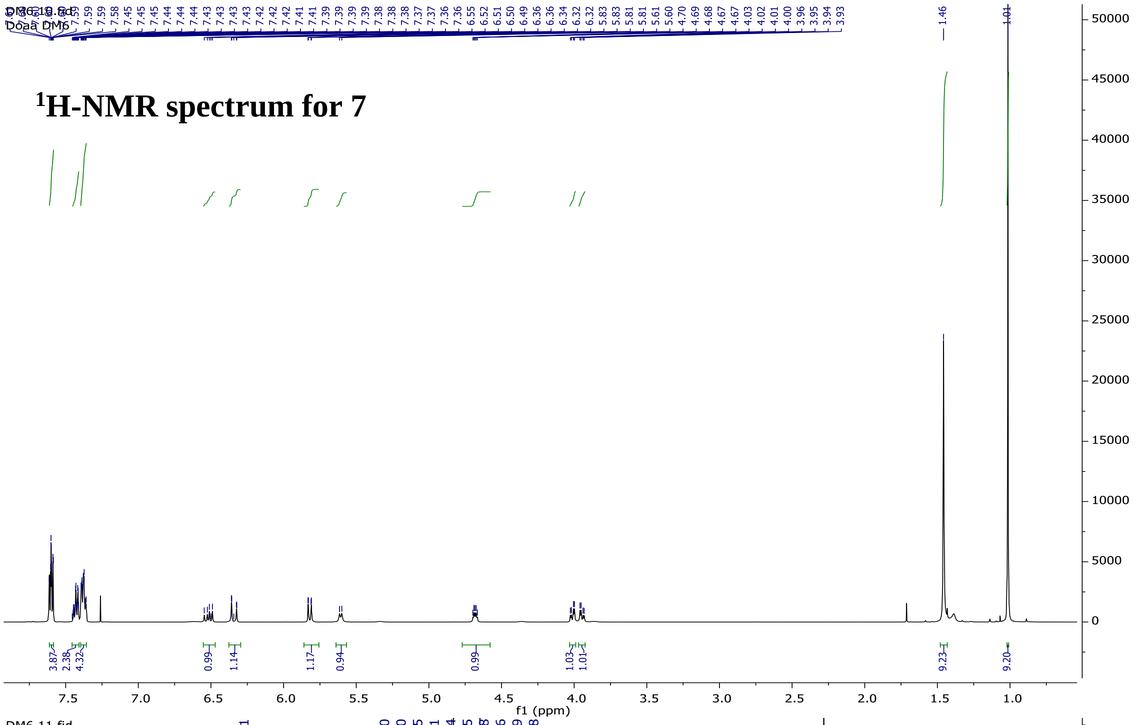

^{13}C-NMR spectrum for 7

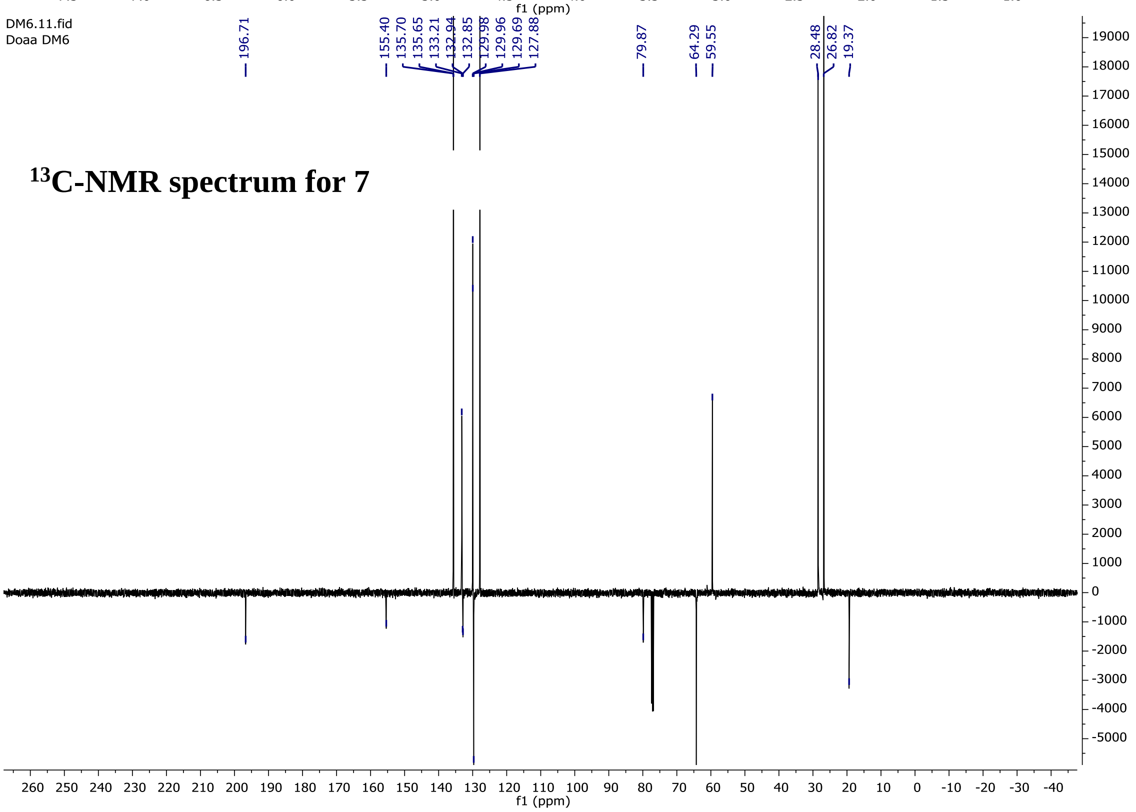

^{1}H-NMR spectrum for 8

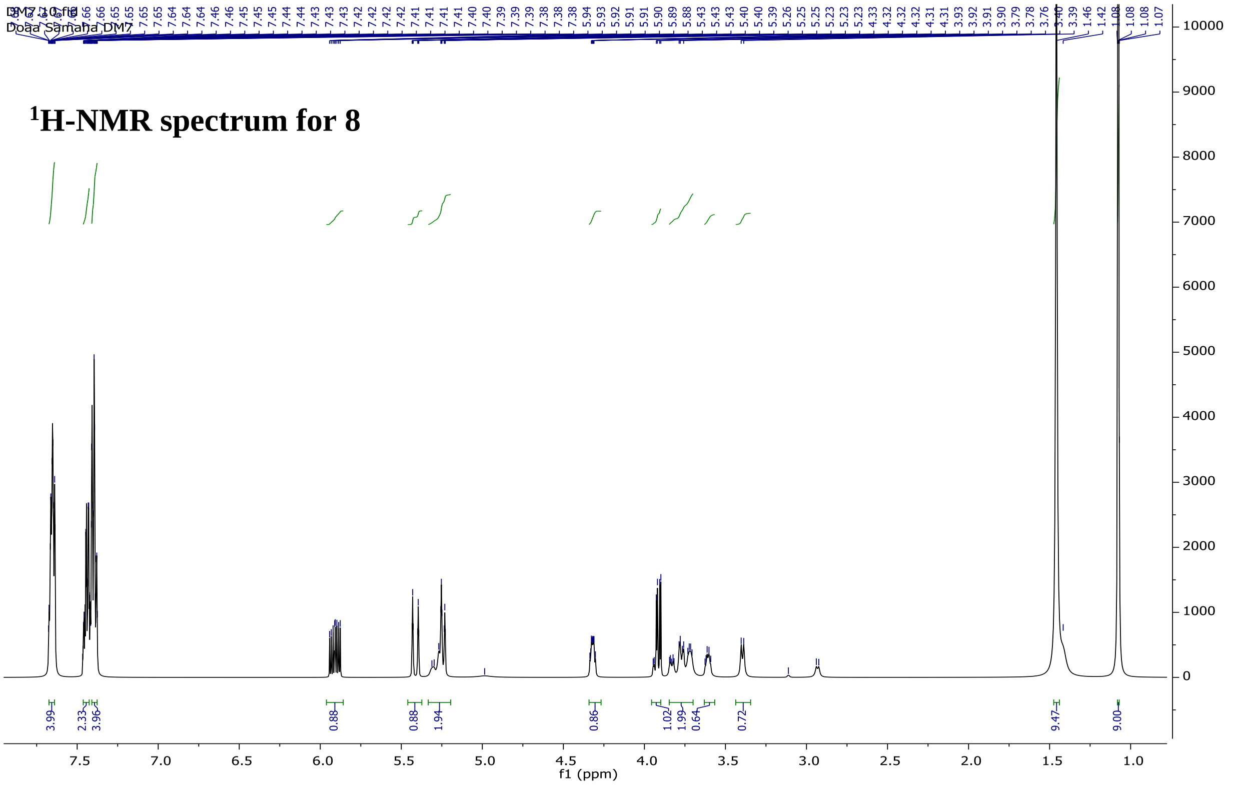

^{13}C-NMR spectrum for 8

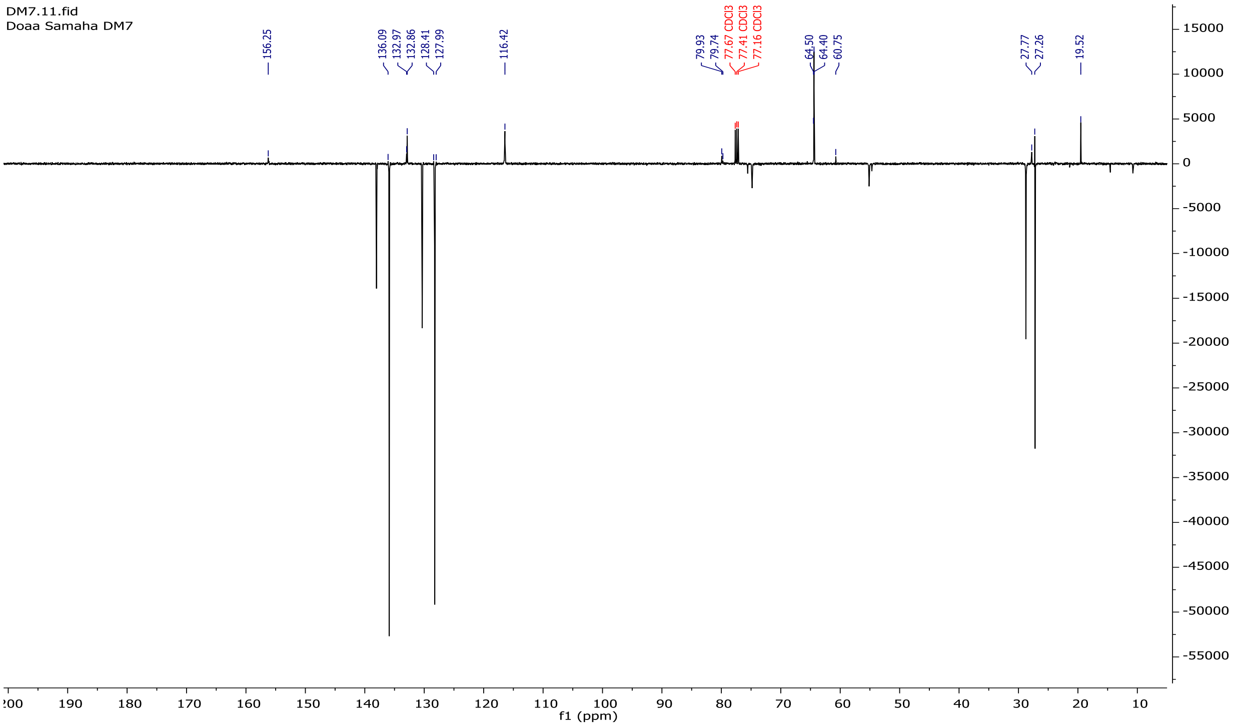

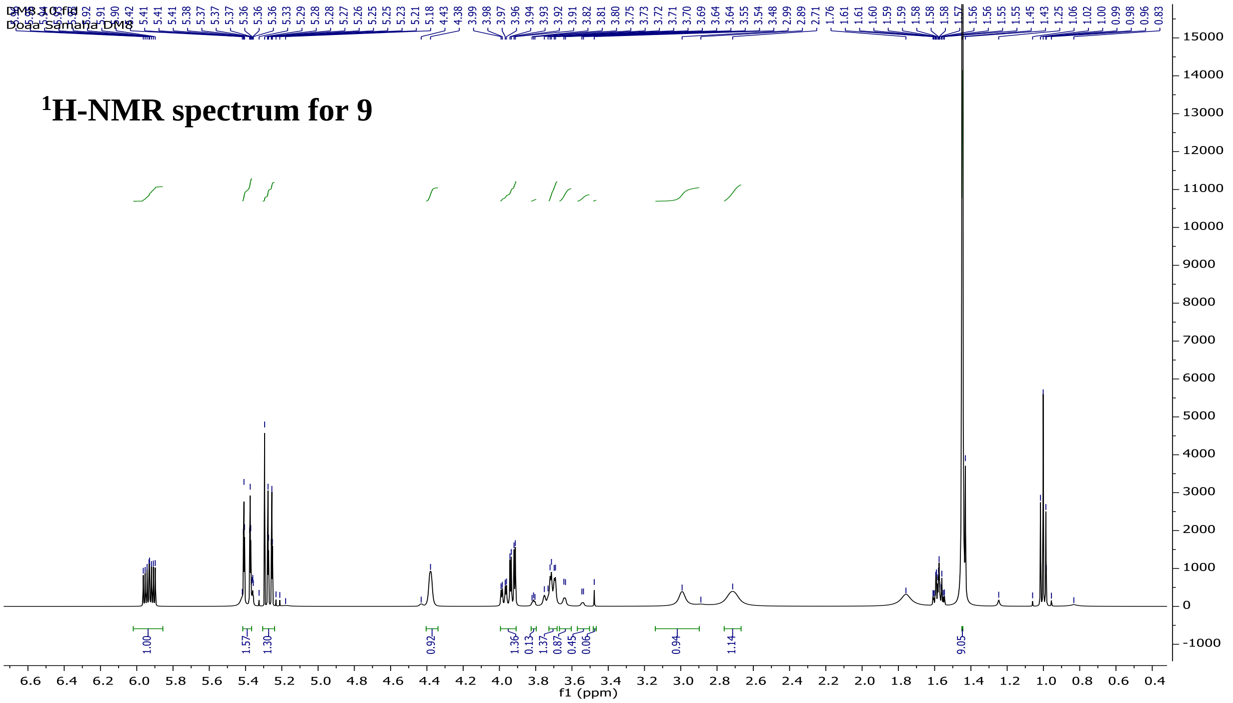

^{1}H-NMR spectrum for 9

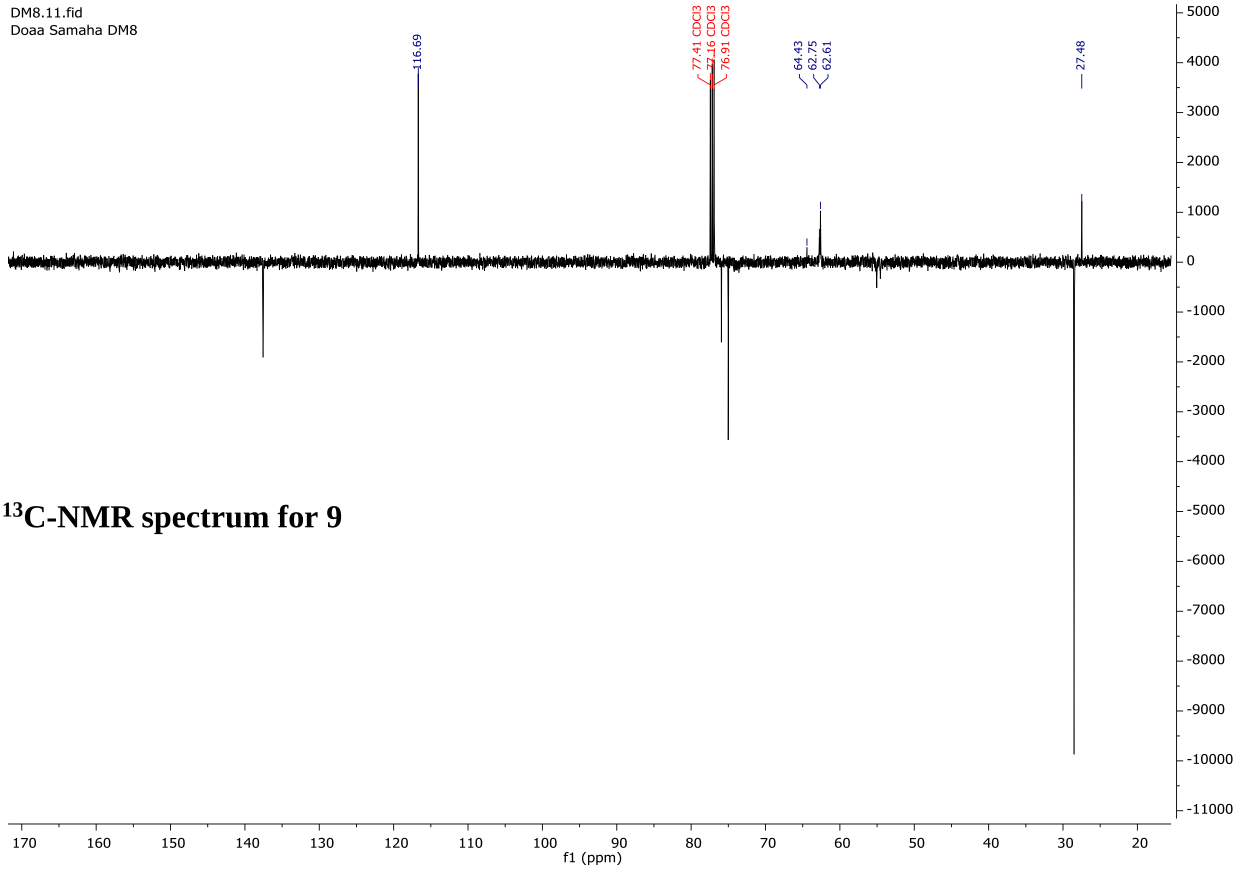

^{13}C-NMR spectrum for 9

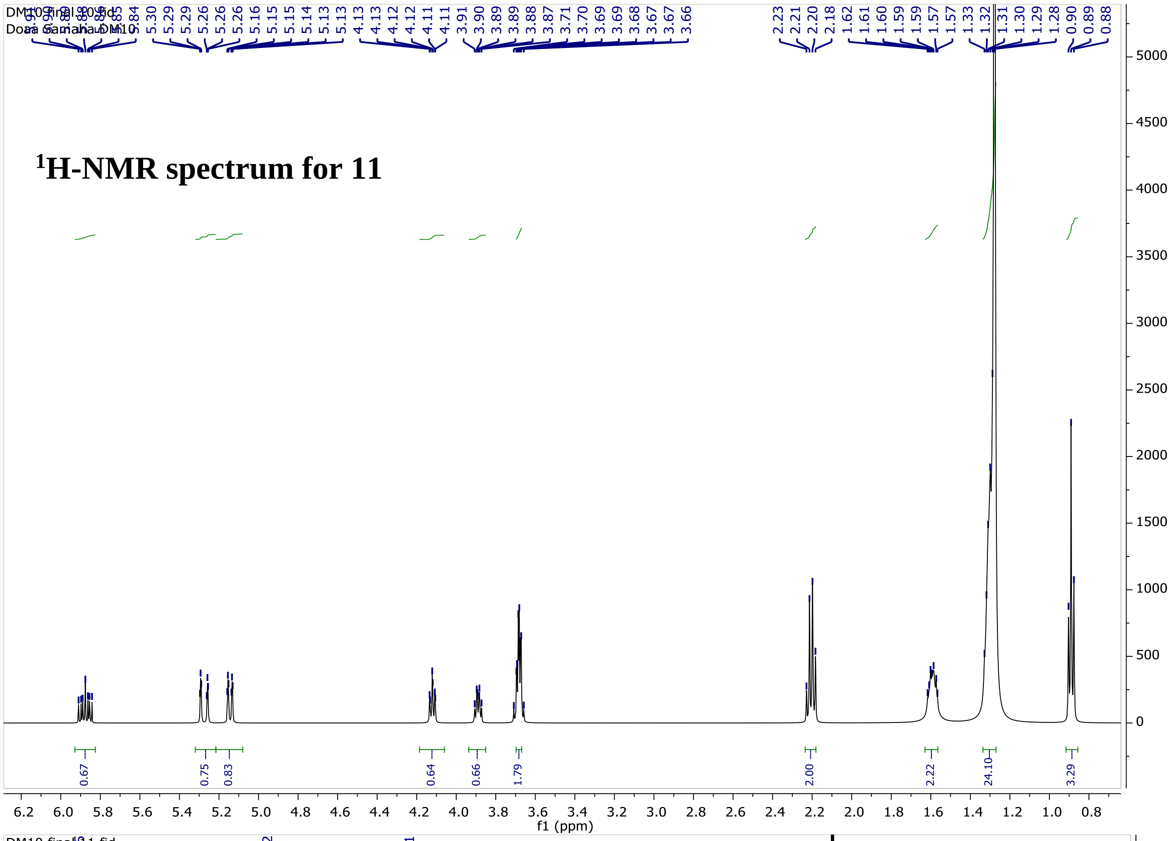

¹H-NMR spectrum for 11
DM10 final 10.fid
Doaa Samaha DM10
f1 (ppm)

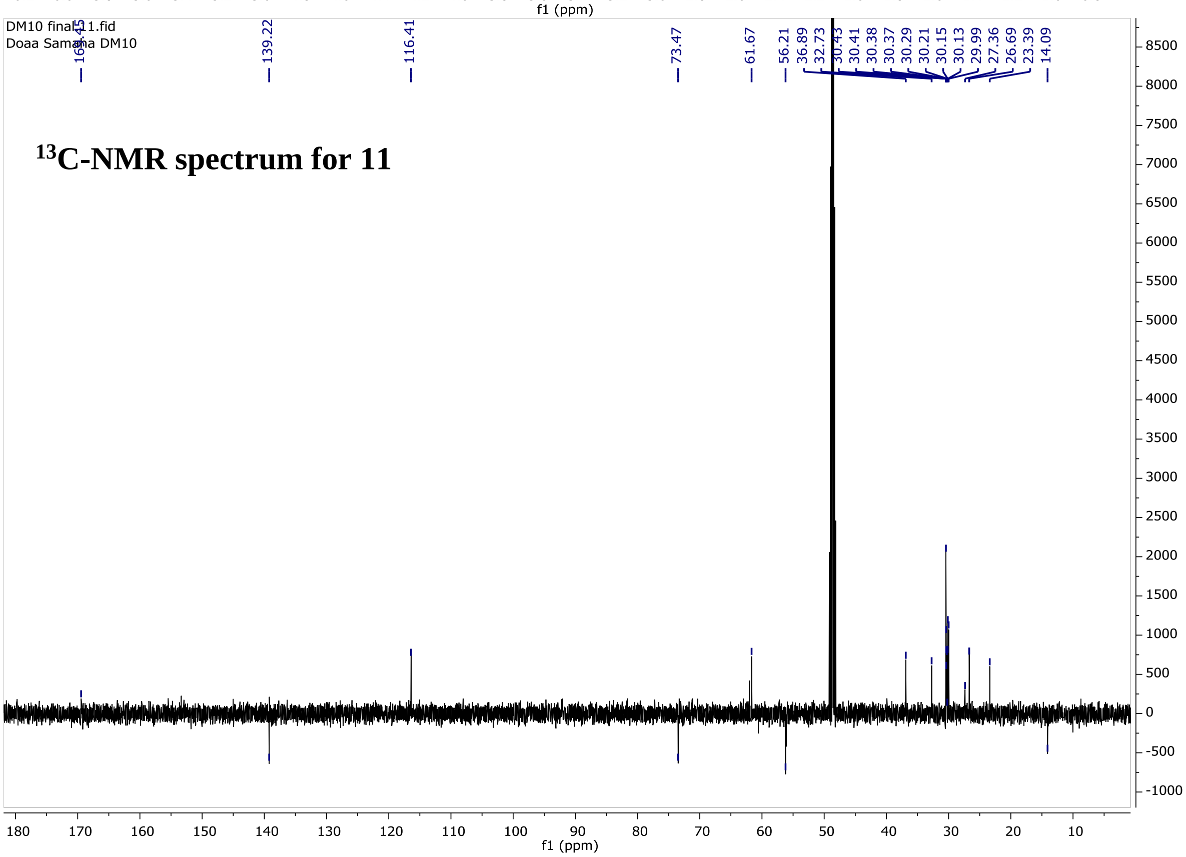

¹³C-NMR spectrum for 11
DM10 final 11.fid
Doaa Samaha DM10
139.22
116.41
73.47
61.67
56.21
36.89
32.73
30.43
30.41
30.38
30.37
30.29
30.21
30.15
30.13
29.99
27.36
26.69
23.39
14.09
f1 (ppm)

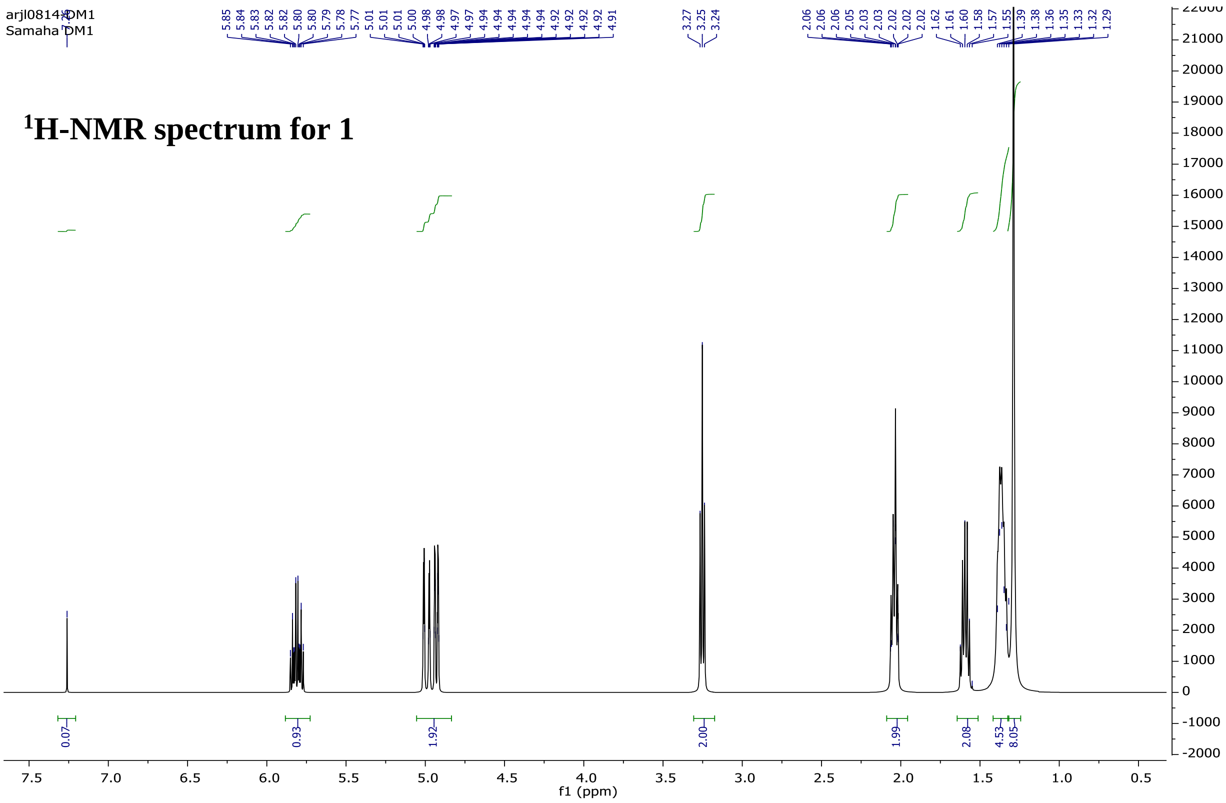
arjl0814-DM1
Samaha DM1
5.85 5.84 5.83 5.82 5.82 5.80 5.80 5.79 5.78 5.77 5.01 5.01 5.00 4.98 4.98 4.97 4.97 4.94 4.94 4.94 4.94 4.94 4.92 4.92 4.92 4.92 4.91
3.27 3.25 3.24
2.06 2.06 2.05 2.03 2.03 2.02 2.02 2.02 1.62 1.61 1.60 1.58 1.57 1.55 1.39 1.38 1.36 1.35 1.33 1.32 1.29
¹H-NMR spectrum for 1
0.07 0.93 1.92 2.00 1.99 2.08 4.53 8.05
f1 (ppm)

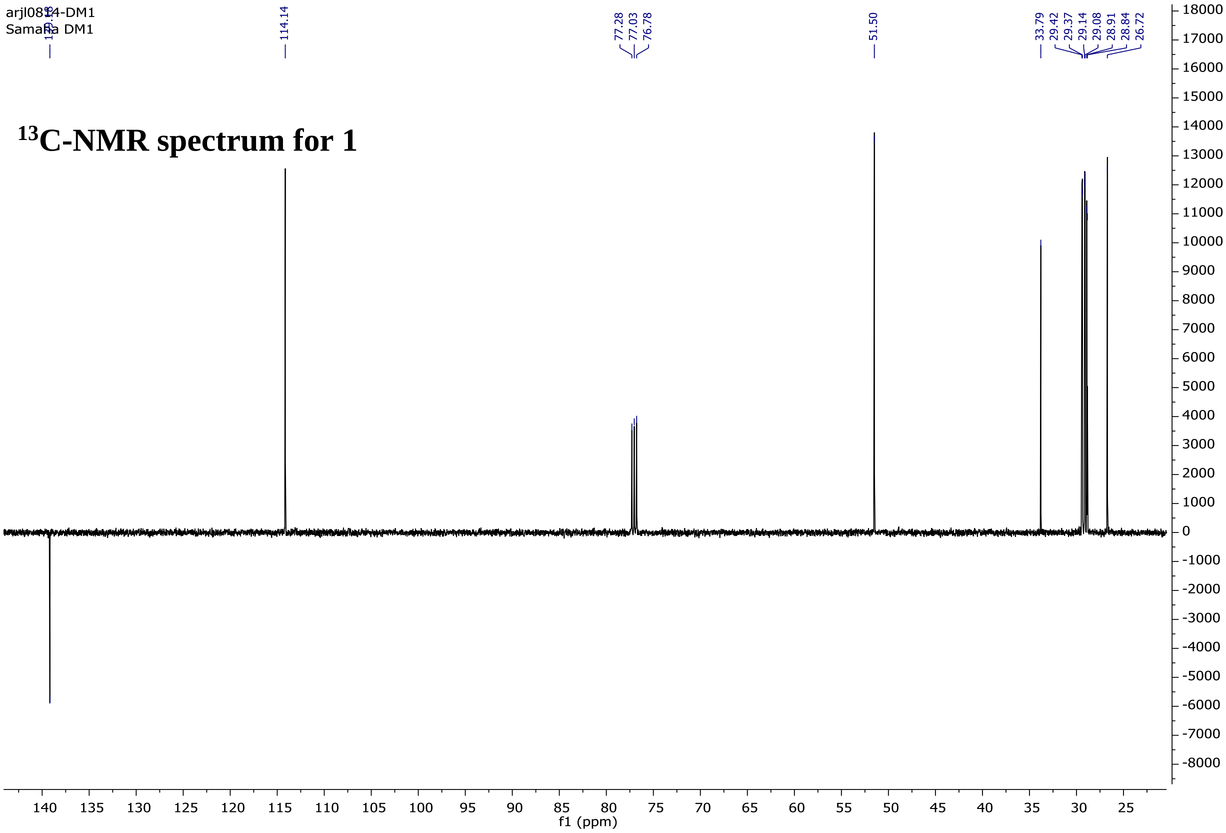
arjl0814-DM1
Samaha DM1
114.14
77.28 77.03 76.78
51.50
33.79 29.42 29.37 29.14 29.08 28.91 28.84 26.72
¹³C-NMR spectrum for 1
f1 (ppm)

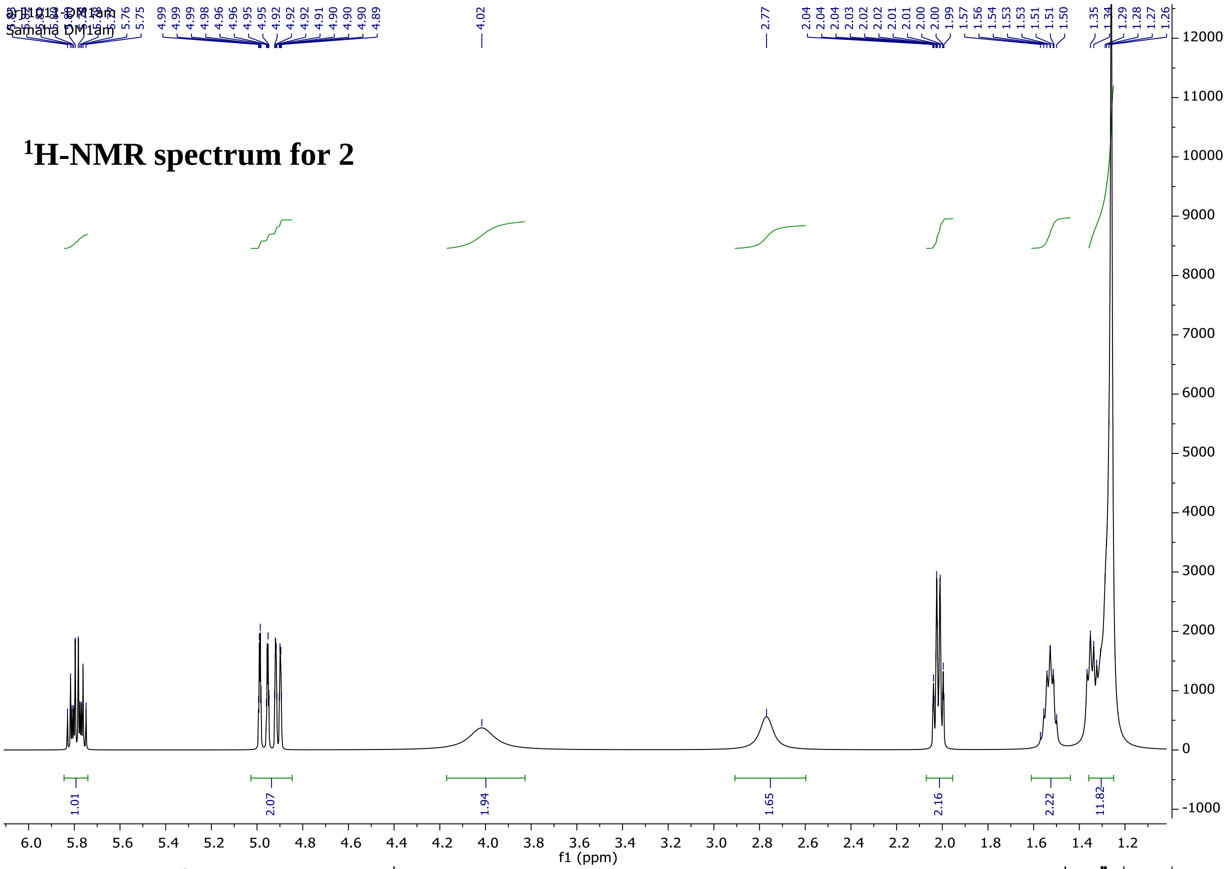
arjl1011-DM1am
Samaha DM1am
1H-NMR spectrum for 2
4.99 4.99 4.99 4.98 4.96 4.96 4.95 4.95 4.92 4.92 4.92 4.91 4.90 4.90 4.90 4.89
4.02
2.77
2.04 2.04 2.04 2.03 2.02 2.02 2.01 2.01 2.00 2.00 1.99
1.57 1.56 1.54 1.53 1.53 1.51 1.51 1.50
1.35 1.34 1.29 1.28 1.27 1.26
1.01
2.07
1.94
1.65
2.16
2.22
11.82
f1 (ppm)

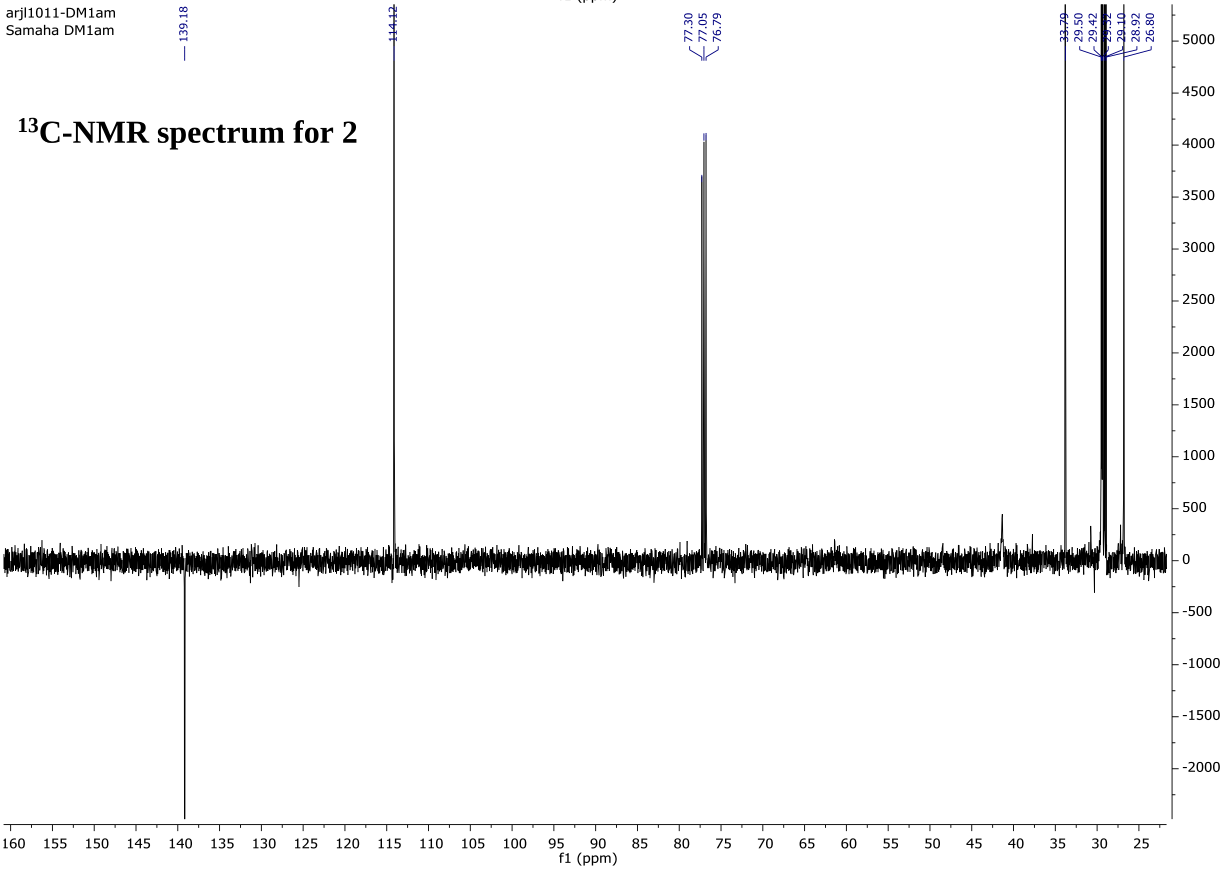
arjl1011-DM1am
Samaha DM1am
13C-NMR spectrum for 2
139.18
114.12
77.30 77.05 76.79
33.79 29.50 29.42 29.32 29.10 28.92 26.80
f1 (ppm)

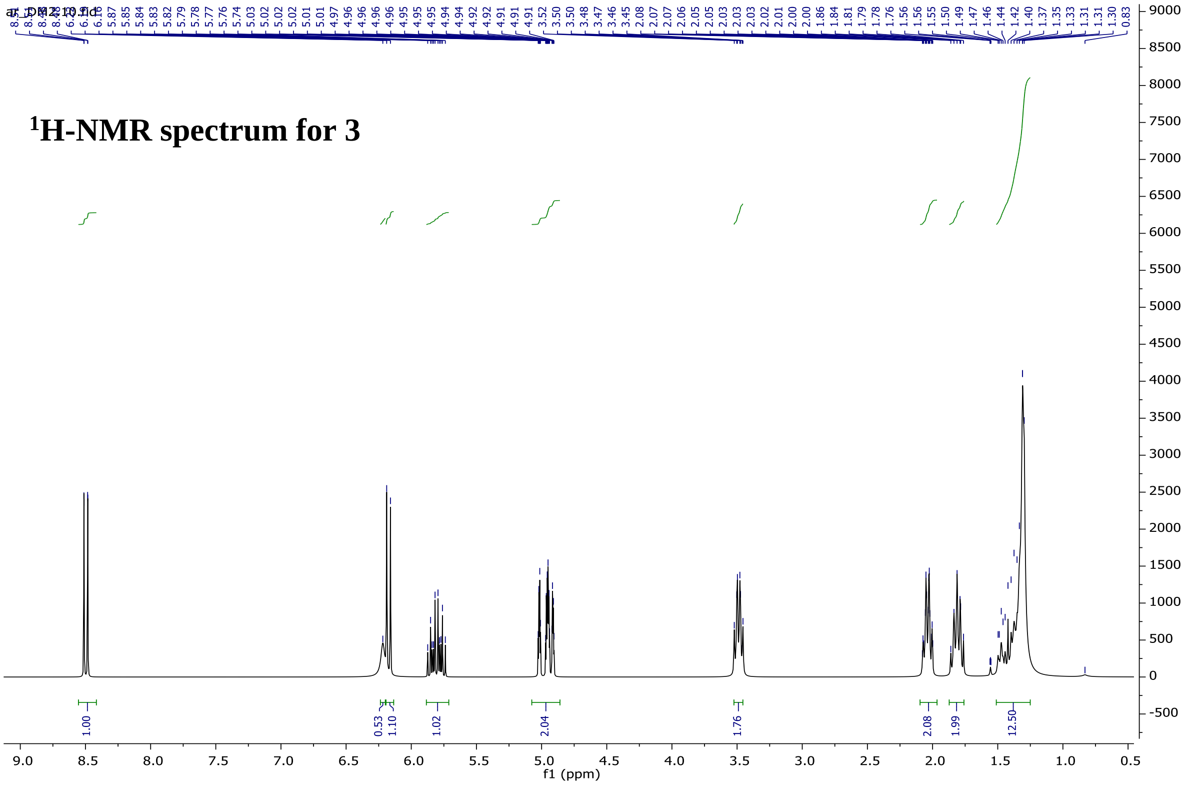
ar_DM2.10.fid
6.18
5.87 5.85 5.84 5.83 5.82 5.79 5.78 5.77 5.76 5.74
5.03 5.02 5.02 5.01 5.01 4.97 4.96 4.96 4.96 4.95 4.95 4.95 4.94 4.94 4.92 4.92 4.91 4.91 4.91
3.52 3.50 3.50 3.48 3.47 3.46 3.45
2.08 2.07 2.07 2.06 2.05 2.05 2.03 2.03 2.03 2.02 2.01 2.00 2.00
1.86 1.84 1.81 1.79 1.78 1.76
1.56 1.56 1.55 1.50 1.49 1.47 1.46 1.44 1.42 1.40 1.37 1.35 1.33 1.31 1.31 1.30 0.83
1H-NMR spectrum for 3
1.00
0.53
1.10
1.02
2.04
1.76
2.08
1.99
12.50
f1 (ppm)

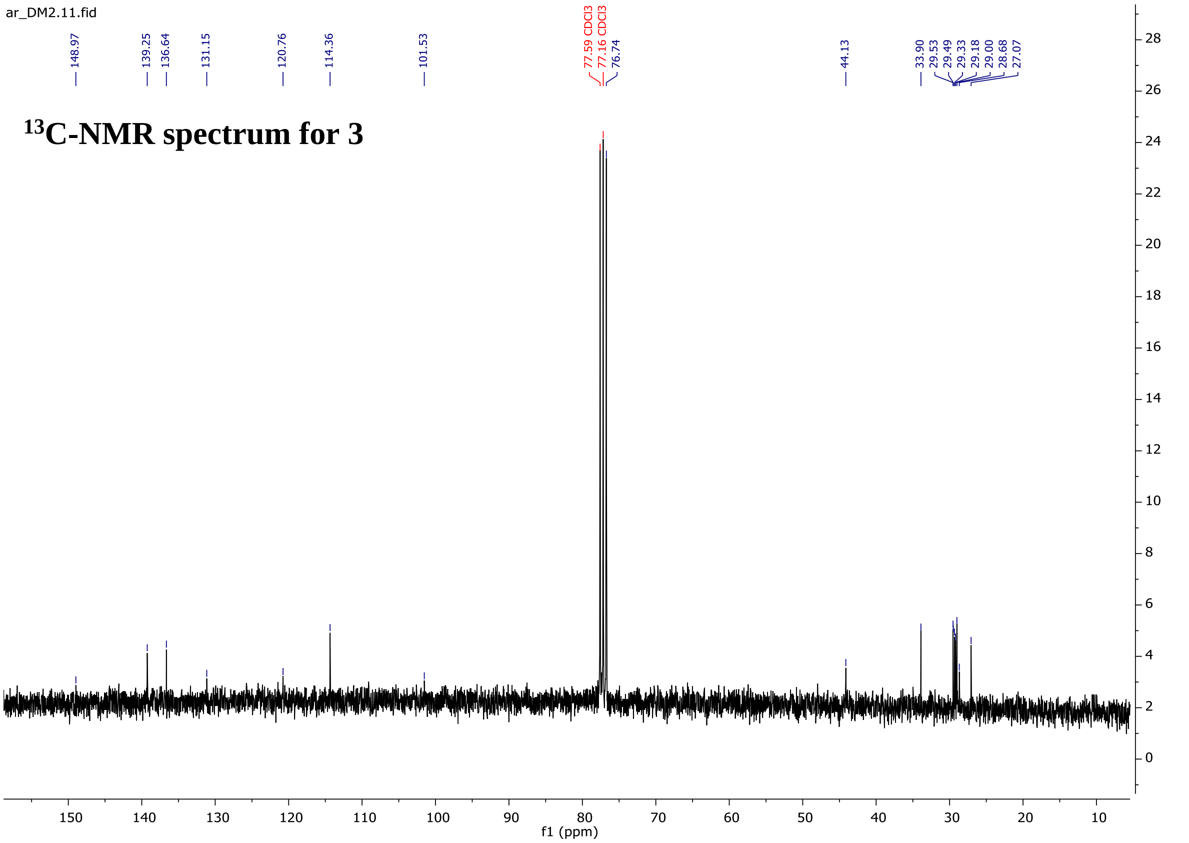
ar_DM2.11.fid
148.97
139.25
136.64
131.15
120.76
114.36
101.53
77.59 CDCl3
77.16 CDCl3
76.74
44.13
33.90
29.53
29.49
29.33
29.18
29.00
28.68
27.07
13C-NMR spectrum for 3
f1 (ppm)

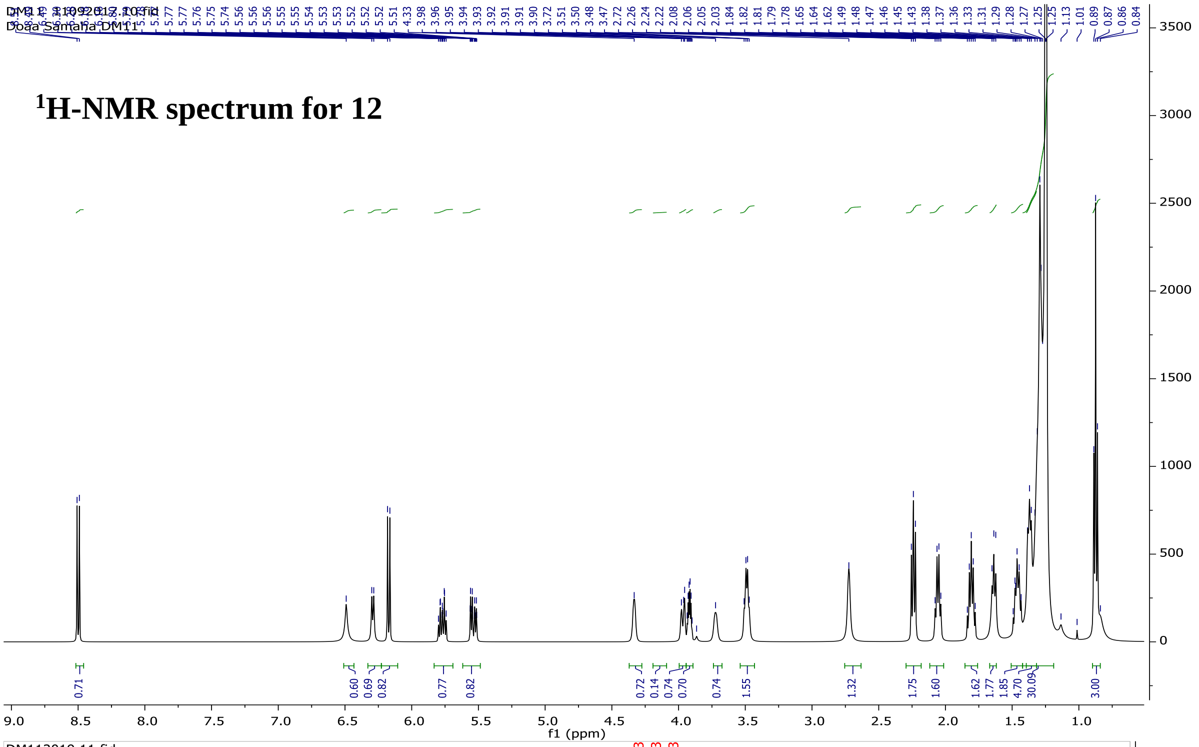
DM11 P 11092019.10.fid
Doaa Samaha DM11
5.77 5.77 5.76 5.75 5.74 5.56 5.56 5.55 5.55 5.54 5.53 5.53 5.52 5.52 5.52 5.51 4.33 3.98 3.96 3.95 3.94 3.93 3.92 3.91 3.91 3.90 3.72 3.51 3.50 3.48 3.47 2.72 2.26 2.24 2.22 2.08 2.06 2.05 2.03 1.84 1.82 1.81 1.79 1.78 1.65 1.64 1.62 1.49 1.48 1.47 1.46 1.45 1.43 1.38 1.37 1.36 1.33 1.31 1.29 1.28 1.27 1.25 1.25 1.13 1.01 0.89 0.87 0.86 0.84
¹H-NMR spectrum for 12
0.71 0.60 0.69 0.82 0.77 0.82 0.72 0.14 0.74 0.70 0.74 1.55 1.32 1.75 1.60 1.62 1.77 1.85 4.70 30.09 3.00
f1 (ppm)

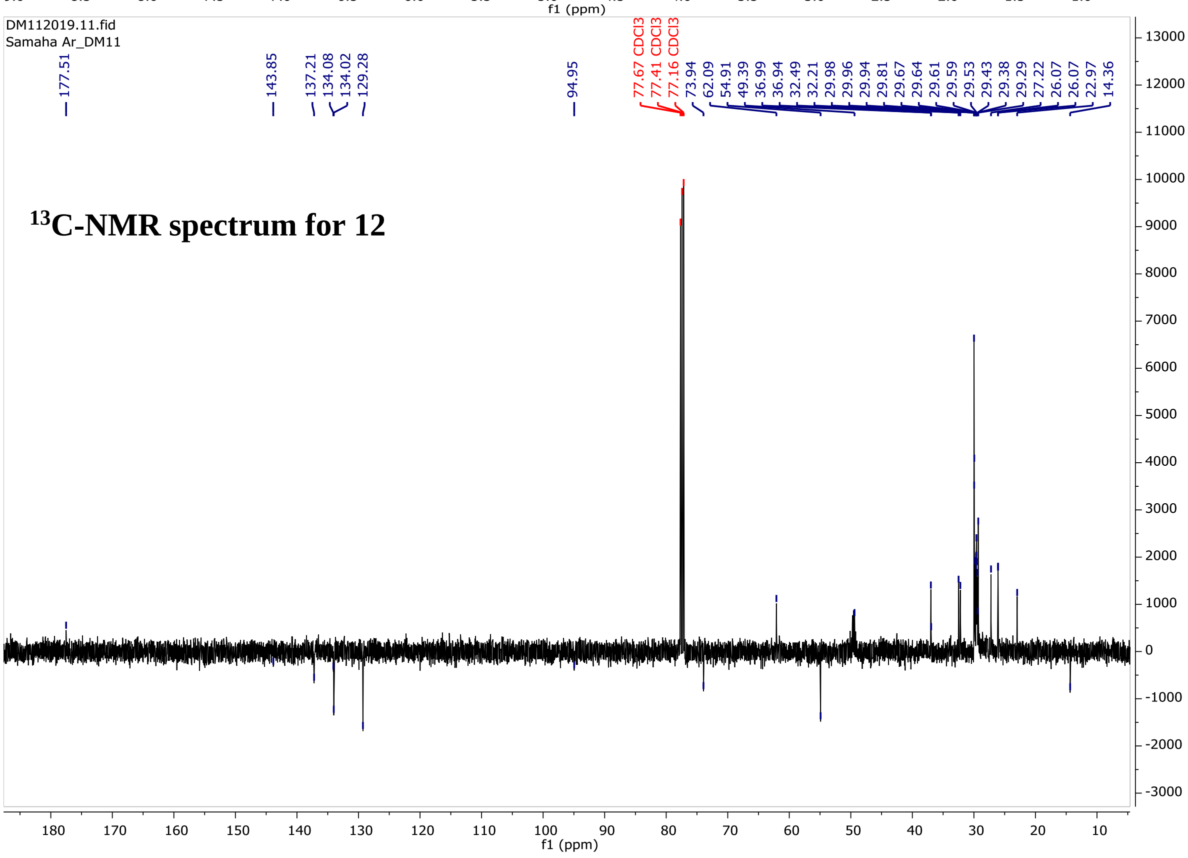
DM112019.11.fid
Samaha Ar_DM11
177.51 143.85 137.21 134.08 134.02 129.28 94.95 77.67 CDCl3 77.41 CDCl3 77.16 CDCl3 73.94 62.09 54.91 49.39 36.99 36.94 32.49 32.21 29.98 29.96 29.94 29.81 29.67 29.64 29.61 29.59 29.53 29.43 29.38 29.29 27.22 26.07 26.07 22.97 14.36
¹³C-NMR spectrum for 12
f1 (ppm)

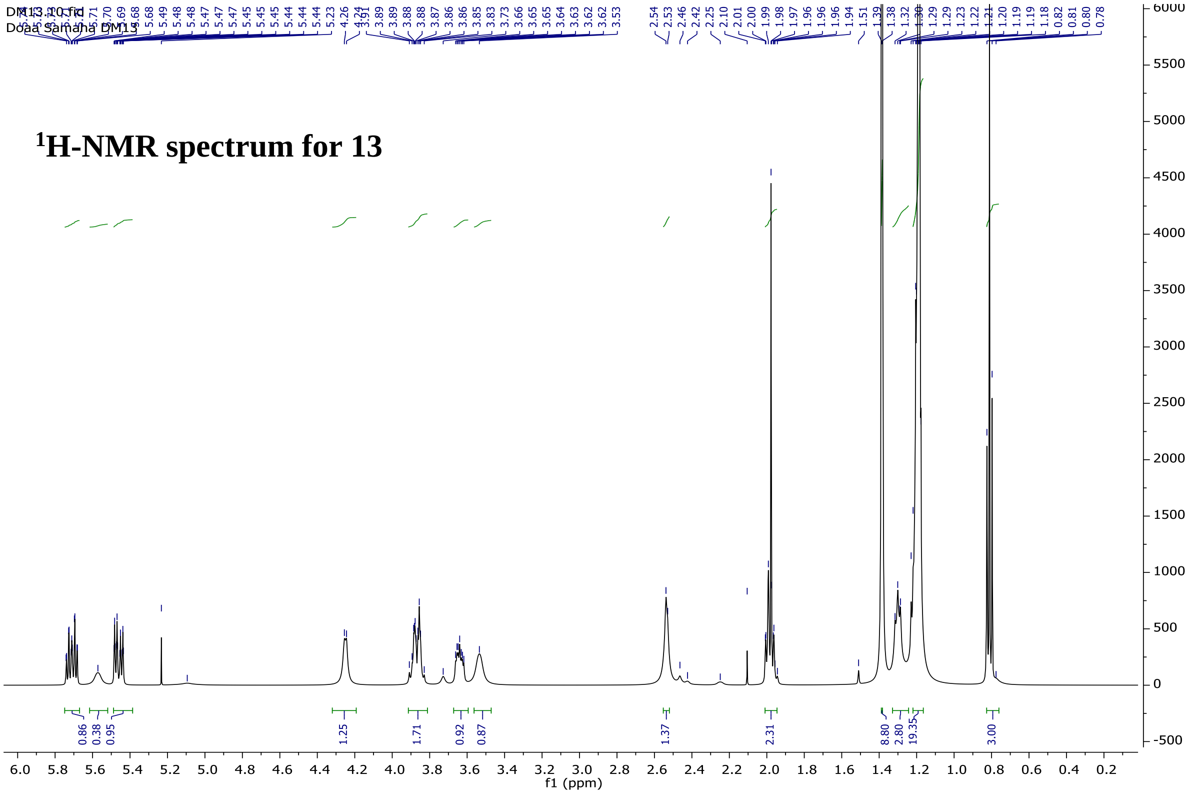

^{1}H-NMR spectrum for 13

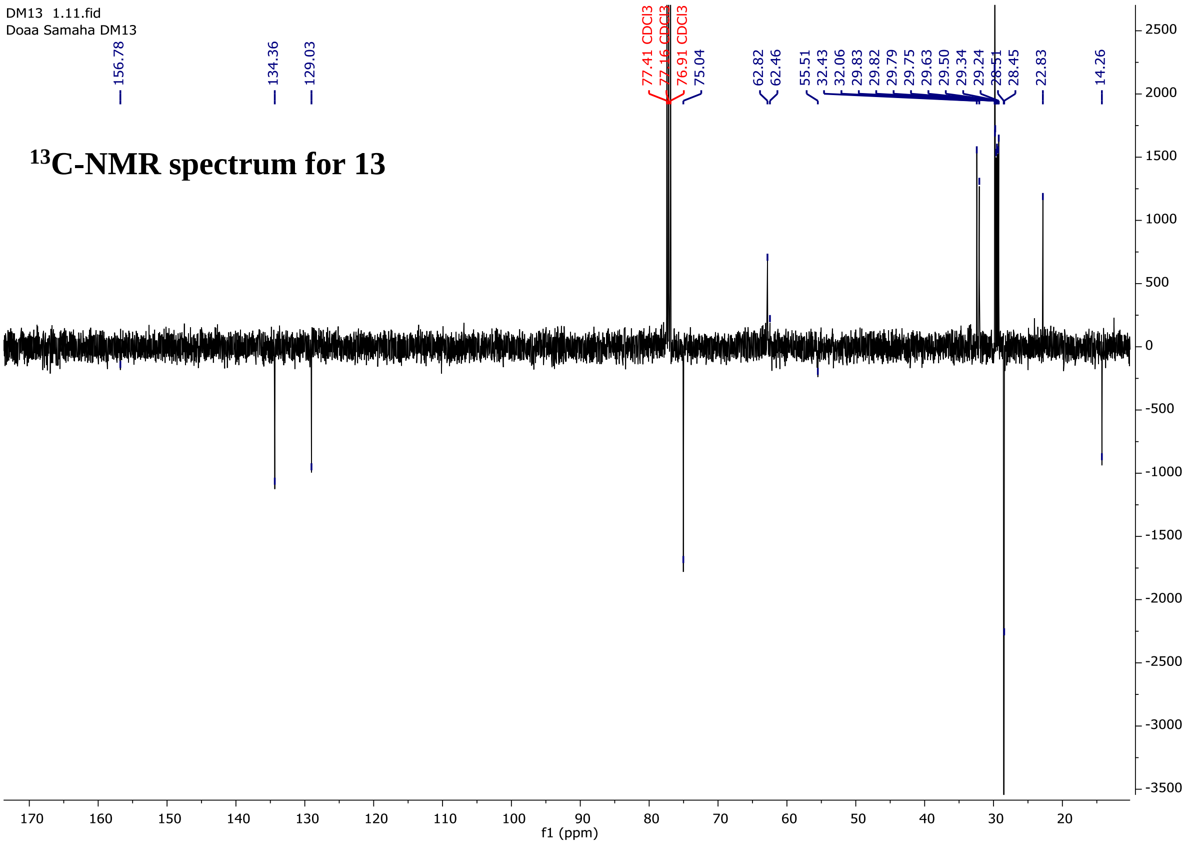

^{13}C-NMR spectrum for 13

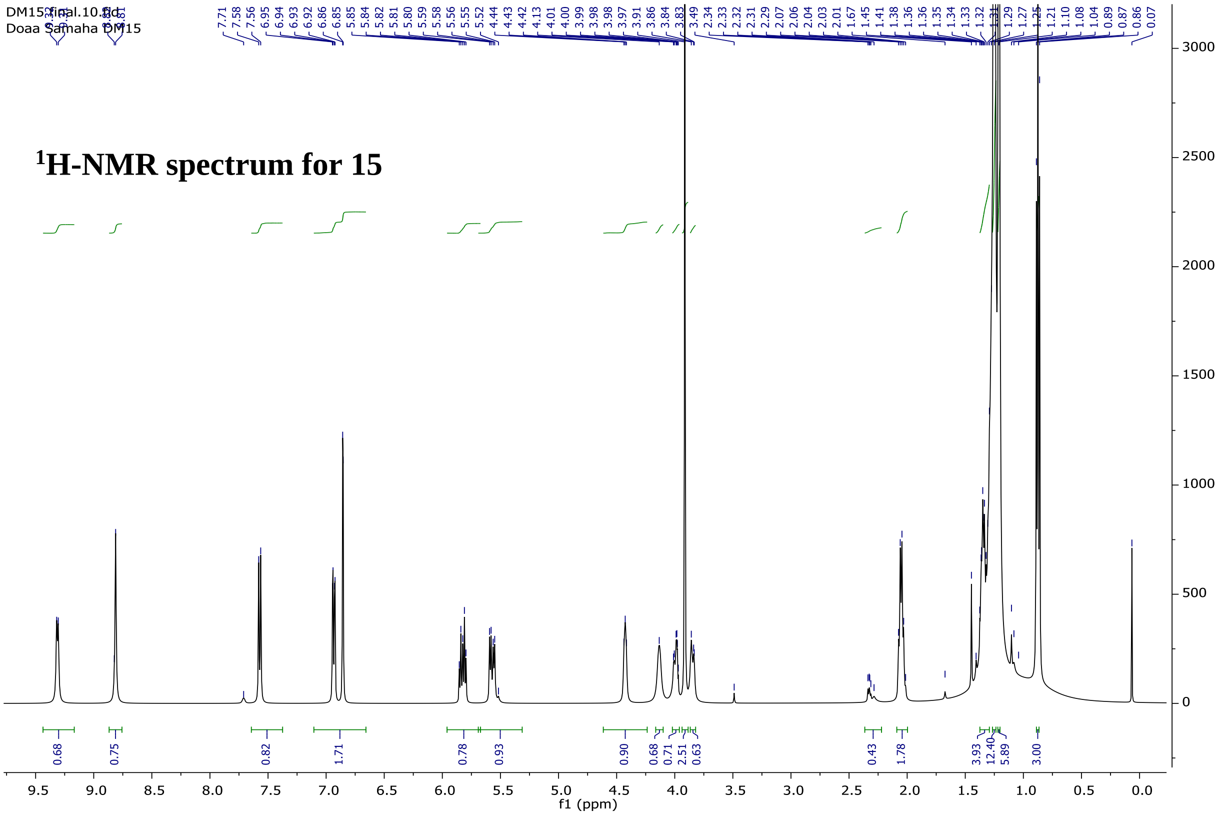

^{1}H-NMR spectrum for 15

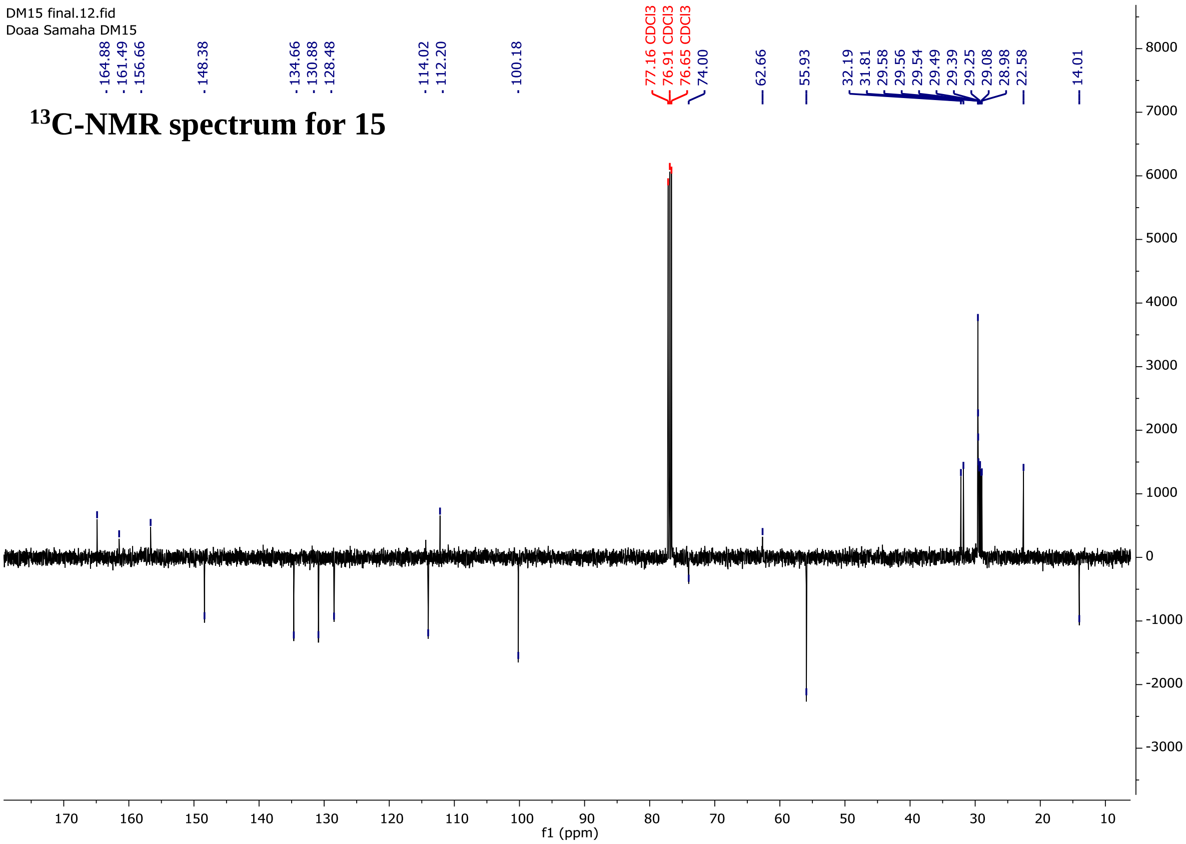

^{13}C-NMR spectrum for 15
